# Targeting SQLE-mediated cholesterol metabolism to enhance CD8+ T cell activation and immunotherapy efficacy in hepatocellular carcinoma

**DOI:** 10.1136/jitc-2025-012345

**Published:** 2025-09-26

**Authors:** Shuang Qiao, Hao Zou, Yulan Weng, Yi-fan Liu, Weihao Li, Xing-Juan Yu, Lian Li, Limin Zheng, Jing Xu

**Affiliations:** 1 State Key Laboratory of Oncology in South China, Guangdong Provincial Clinical Research Center for Cancer, Sun Yat-sen University Cancer Center, Guangzhou, People's Republic of China; 2 Ministry of Education Key Laboratory of Gene Function and Regulation, School of Life Sciences, Sun Yat-sen University, Guangzhou, People's Republic of China

**Keywords:** Hepatocellular Carcinoma, Tumor Microenvironment

## Abstract

**Background:**

The sustained effectiveness of anti-programmed cell death protein-1 (PD1) treatment is limited to a subgroup of patients with hepatocellular carcinoma (HCC) due to the tumor microenvironment heterogeneity, highlighting the need to identify targetable biomarkers that synergize with PD1 blockade. Abnormal cholesterol metabolism plays a critical role in HCC progression, along with growing evidence indicating its complex immunomodulatory effects within the tumor microenvironment. However, the interplay between cholesterol homeostasis and immune evasion remains elusive.

**Methods:**

Transcriptomic and clinical data from HCC datasets were analyzed to identify cholesterol metabolism-related targets. Multiplex immunostaining and flow cytometry were applied to examine the immune landscape association with squalene epoxidase (SQLE) in human and murine tumors. Mechanistic studies were conducted in vitro, and co-culture experiments of tumor cells and T cells were followed by metabolomics and transcriptome analyses. Therapeutic efficacy was evaluated in mouse HCC models.

**Results:**

We demonstrated that elevated SQLE expression in human HCC was associated with poor clinical outcomes and correlated with reduced CD8^+^ T cell infiltration and activation. Pharmacological inhibition or genetic knockdown of SQLE in tumor cells promoted CD8^+^ T cell proliferation and activation in co-culture experiments. Untargeted metabolomics identified 27-hydrocholesterol, an oxysterol derived from tumor cells, as a key factor impairing CD8^+^ T cell function via cholesterol dysregulation. SQLE inhibition in tumor cells suppressed oxysterols secretion, therefore overcoming cholesterol restrictions and enhancing the immune responses of CD8^+^ T cells. Moreover, SQLE targeting with terbinafine restored antitumor immunity and synergized with anti-PD1 therapy in HCC.

**Conclusion:**

Targeting tumorous SQLE restores CD8^+^ T cell function by overcoming cholesterol restrictions via oxysterol-SREBP2 signaling, highlighting SQLE as a potential therapeutic target to enhance immunotherapy efficacy in HCC.

**WHAT IS ALREADY KNOWN ON THIS TOPIC**

**WHAT THIS STUDY ADDS**

- This study demonstrates that targeting squalene epoxidase (SQLE) can enhance the immunotherapeutic response and restore the antitumor function of CD8^+^ T cells by overcoming cholesterol restrictions via oxysterol-*SREBP2* signaling.

**HOW THIS STUDY MIGHT AFFECT RESEARCH, PRACTICE OR POLICY**

- This study shows that elevated SQLE expression in human hepatocellular carcinoma (HCC) is associated with poor clinical outcomes and reduced responsiveness to anti-programmed cell death protein-1 therapy. Furthermore, these results reveal a novel mechanism by which SQLE regulates CD8^+^ T cell immune function, highlighting SQLE as both a prognostic biomarker for immunotherapy response and a potential therapeutic target to improve immunotherapy efficacy in HCC.

## Introduction

 Hepatocellular carcinoma (HCC) is the most common type of liver cancer and a leading cause of cancer-related deaths worldwide.^1 2^ Despite significant advances in immunotherapy, clinical benefits remain limited to a small proportion of patients with HCC, underscoring the urgent need for novel therapeutic strategies. Increasing evidence suggests that the metabolic plasticity of tumors plays a crucial role in shaping the local immune microenvironment, promoting tumor progression, while creating opportunities to integrate metabolic therapies with immunotherapy.^3^ Identifying druggable metabolic targets that modulate immune response and foster a therapeutic-supportive niche is essential for improving the efficacy of immunotherapy.

The liver plays a pivotal role in maintaining cholesterol homeostasis, and aberrant cholesterol metabolism is a prominent feature of HCC.^4^ Cholesterol is an essential component of cell membranes to regulate membrane fluidity and signal transduction, which is critical in supporting immune cell functionality across various disease contexts. For example, cholesterol enrichment caused by the absence of ABCG1 impairs cholesterol efflux, promoting the differentiation of regulatory T cells in atherosclerosis; while reduced cholesterol levels in macrophages induced by cholesterol efflux are associated with the generation of immunosuppressive macrophages in HCC.^5 6^ Furthermore, studies have suggested that cholesterol deficiency significantly impairs cytotoxic T lymphocytes (CTLs) proliferation and activation, hindering their antitumor functions.^7^,^9^ Given the crucial role of cholesterol metabolism in tumor progression and therapeutic efficacy, it is imperative to further investigate the precise mechanisms by which tumor cells regulate immune cell function via cholesterol metabolism.

Squalene epoxidase (SQLE), the second rate-limiting enzyme in cholesterol biosynthesis, is significantly upregulated in various tumors, including HCC, and is closely associated with poor prognosis.^10^,^15^ Functional studies have demonstrated that SQLE promotes HCC progression by enhancing tumor cell proliferation, epithelial–mesenchymal transition, and metastasis.^12 13 16^ Beyond its role in cholesterol biosynthesis, SQLE contributes to cholesteryl ester accumulation and alters the NADP^+^/NADPH ratio, leading to oxidative stress and promoting fatty acid synthesis, underscoring its broad involvement in tumor biology. ^17^ Recent evidence suggests that SQLE influences the immune landscape of tumors. For example, squalene accumulation on SQLE inhibition suppresses NF-κB signaling and reduces the recruitment of immunosuppressive cells, such as myeloid-derived suppressor cells and tumor-associated macrophages. ^18^ Moreover, SQLE activation has been shown to enhance programmed death-ligand 1 stability, thereby promoting immune evasion in gastric cancer. ^19^ Although these findings suggest SQLE may facilitate tumor immune escape, the precise mechanisms remain incompletely understood.

In this study, we demonstrate that elevated SQLE expression in human HCC is associated with poor clinical outcomes and limited response to anti-programmed cell death protein-1 (PD1) therapy, partly due to reduced infiltration of CD8^+^ T cells. Inhibition of tumorous SQLE decreases the production of 27-hydrocholesterol, restores CD8^+^ T cell function, and enhances anti-PD1 immunotherapy efficacy. These data reveal novel mechanisms by which SQLE regulates the tumor microenvironment (TME) and underscore its potential to improve the effectiveness of immunotherapy in patients with HCC.

## Methods

### Patients and samples

Tissue samples were collected from patients with HCC from Sun Yat-sen University Cancer Center (SYSUCC, Guangzhou, China). A total of 196 patients (cohort 1) who underwent curative resection during 2007 and 2012 were randomly enrolled for survival analysis. Inclusion criteria for patient enrollment were: no prior anticancer therapies, absence of hepatic steatosis confirmed by pathologists, and availability of follow-up data. 23 patients with advanced HCC treated with anti-PD1 monoclonal antibodies before surgical resection were further enrolled (cohort 2). Patient responses were evaluated using the RECIST (Response Evaluation Criteria in Solid Tumors). The clinical characteristics of these two groups are summarized in online supplemental table 1. Overall survival (OS) was defined as the interval between the date of surgery and death or the last observation of surviving patients. All the samples were coded anonymously in accordance with local ethical guidelines, as stipulated in the Declaration of Helsinki. The Institutional Review Board of SYSUCC approved this study.

### Animal models

6–8 weeks old male C57BL/6 and NCG mice were purchased from Charles River Laboratories and Jackson Laboratories. All mice were housed under specific pathogen-free conditions and handled according to the Animal Care and Use Committee guidelines of SYSUCC.

### Cell lines

The HCC cell line Huh7 (Shanghai Life Academy of Sciences cell library) and murine HCC cell line Hepa 1–6 (American Type Culture Collection (ATCC)) were cultured in Dulbecco’s Modified Eagle’s Medium supplemented with 10% fetal bovine serum (FBS), 100 units/mL penicillin, and 100 mg/mL streptomycin. The HCC cell line SNU449 (ATCC) and Jurkat cells (ATCC) were maintained in Roswell Park Memorial Institute (RPMI)-1640 medium supplemented with 10% FBS, 100 units/mL penicillin, and 100 mg/mL streptomycin. For Jurkat cells, an additional 55 mM 2-mercaptoethanol was added.

### Human CD8^+^ T cell isolation

Buffy coats from healthy donors were obtained from the Guangzhou Blood Center, with informed consent collected from all subjects. Peripheral blood mononuclear cells were isolated by density gradient centrifugation. CD8^+^ T cells were isolated with the EasySep Human CD8^+^ T Cell Isolation Kit following the manufacturer’s instructions. Freshly isolated CD8^+^ T cells were maintained in RPMI-1640 supplemented with 10% FBS, 300 mg/L L-glutamine, 100 units/mL penicillin, 100 mg/mL streptomycin, 1 mM sodium pyruvate, 100 mM NEAA, 1 mM HEPES, and 55 mM 2-mercaptoethanol. All cells were cultured at 37℃ with 5% CO_2_ and verified to be mycoplasma-free.

### Tumor–T cell co-culture

SNU449 or Huh7 cells (5×10^3^) were seeded into 96-well plates. After attachment, cells were either transfected by small interfering RNA (siRNA) oligonucleotides targeting human SQLE using Lipofectamine RNAiMAX (Invitrogen) following the manufacturer’s instructions, or treated with 10 µM terbinafine. The medium was changed after 24 hours, and human CD8^+^ T cells were co-cultured with the tumor cells at a ratio of 5:1 in the presence of 5 µg/mL anti-CD3 and 2 µg/mL anti-CD28. CD8^+^ T cells were labeled with carboxyfluorescein succinimidyl amino ester (CFSE) in proliferation assay. Jurkat cells were co-cultured with the tumor cells in the presence of 150 ng/mL phytohemagglutinin (PHA) for 72 hours. After 72 hours of incubation, the co-cultured cells were subjected to subsequent analysis.

### T cell functional assays

For the proliferation assay, isolated naïve CD8^+^ T cells were labeled with CFSE (Invitrogen) according to the manufacturer’s protocol and co-cultured with the indicated tumor cells for 72 hours. Proliferation was analyzed using flow cytometry. For activation assessment, cells were stained with anti-CD69 and anti-PD-1 antibodies (BioLegend) and analyzed by flow cytometry. For intracellular staining, 5 µg/mL Brefeldin A was added 4 hours before cells were collected. Cells were then fixed using the FOXP3/Transcription Factor Staining Buffer Set (Thermo Fisher Scientific) and stained with anti-interferon-gamma (IFNγ) (BioLegend) and anti-Ki-67 (BioLegend) for flow cytometry. IFN-γ production in culture supernatants was measured on day 3 poststimulation using the Human IFN-γ Precoated ELISA Kit (Dakewe).

### Cell proliferation assay

SNU449 or Huh7 tumor cells (5×10^3^) were seeded into 96-well plates. Gradient-diluted terbinafine was added into culture medium (CM) with an equal volume of dimethyl sulfoxide (DMSO) used as a control. After 72 hours treatment, CCK8 reagent (DOJINDO) was added to the wells and incubated according to the manufacturer’s instructions. Absorbance at 450 nm was measured using a microplate reader (Thermomax), and IC_50_ curves were plotted.

### Filipin III staining

Cells were fixed with 4% paraformaldehyde and stained with 50 mg/mL filipin III (Sigma-Aldrich) for 30 min at 37℃. Stained cells were imaged using a Zeiss LSM880 confocal microscope in Airyscan mode with a 40×1.3 NA Plan-Apochromat oil objective (Zeiss). Images were acquired and processed with ZEN software (Zeiss).

### Intracellular Ca^2+^ concentration measurement

Human CD8^+^ T cells co-cultured with tumor cells in the presence or absence of terbinafine for 72 hours were incubated with Fluo-4 AM (Beyotime) at 37°C for 30 min, following the manufacturer’s protocol. Excess Fluo-4 AM was removed by two washes with cold calcium-free Hanks' balanced salt solution (HBSS), and cells were resuspended in calcium-free HBSS and kept on ice. Before acquisition, cells were warmed to 37 °C for 5 min. Baseline reading was recorded for 1 min using Cytoflex LX flow cytometer (Beckman) followed by stimulation of intracellular calcium release with 100 ng/mL ionomycin (Merck). For quantification, Fluo-4 AM fluorescence intensity was plotted against time using the kinetic option in FlowJo (Tree Star) with Gaussian smoothing. A fixed time range was selected for both baseline and treatment conditions.

### Cholesterol measurement

The total intracellular cholesterol concentration in the medium and cell lysate was measured using a Total Cholesterol Assay Kit (Jiancheng Bioengineering Institute) according to the manufacturer’s instructions.

### LC-MS non-targeted metabolomics

Fresh medium from Jurkat cells co-cultured with SNU449 cells in the presence or absence of terbinafine was collected, freeze-dried into powder, and analyzed by Meiji Biological Medical Technology (Shanghai, China). Briefly, samples were dried using a SpeedVac, reconstituted in 50% acetonitrile, and sonicated for 30 s. The solutions were centrifuged, and the supernatant was subjected to analysis by high-performance liquid chromatography coupled with high-resolution mass spectrometry and tandem mass spectrometry (HPLC-MS/MS).

Samples were separated by liquid chromatography, and individual components were ionized in the ion source of the high-vacuum mass spectrometer. The mass-to-charge ratio (m/z) was analyzed to generate a mass spectrum. Metabolite acquisition and identification were conducted using Xcalibur V.4.1 and Tracefinder V.4.1 software, respectively. Quantitative enrichment analysis was performed using centered and scaled levels of all detectable metabolites, excluding invariant metabolites, with the SMPDB database to identify biological processes associated with differential expression. Statistical analyses were performed using the MetaboAnalyst V.5.0 web server.

### Oxysterol measurement

Oxysterol extraction and quantification were performed as previously described.^20^ Briefly, cell pellets were resuspended in 70 µL of lysis buffer (0.1% Triton X-100, 40 µL DMSO and 5 µL of butylated hydroxytoluene at 90 mg/mL) and sonicated for 20 min. To 110 µL of the lysates or standards, 190 µL of LC-MS grade methanol (MEOH) and 380 µL of dichloromethane (DCM) were added and vortexed for 20 s. Next, 120 µL of LC-MS grade water was added, vortexed for 10 s, and allowed to stand at room temperature for 10 min. Samples and standards were centrifuged at 7500×g for 10 min at 8°C. The upper and interphase layers were collected and dried for protein normalization. The lower DCM layer was collected separately, dried under nitrogen gas, and reconstituted in 500 µL MEOH and 1.5 mL LC-MS grade water containing 0.1% formic acid. Oasis hydrophilic-lipophilic balanced (HLB) solid-phase extraction (SPE) cartridges (Waters) were used to selectively isolate oxysterols. Cartridges were primed with 800 µL MEOH and 600 µL H_2_O containing 0.1% formic acid. Samples were applied to the cartridges, followed by sequential washes with 600 µL H_2_O (containing 0.1% formic acid) and 600 µL hexane to remove polar non-binding analytes and excess cholesterol. Oxysterols were eluted with 1 mL butyl acetate, collected in fresh tubes, dried under nitrogen gas, and reconstituted in 40 µL of 40% MEOH containing 0.1% formic acid. Oxysterols were quantified by LC/MS (Agilent Technologies) by calculating the ratios of their peak areas to that of the internal standard and comparing these ratios to an external calibration curve prepared in methanol.

### RNA interference and lentiviral transduction

Negative control and human SQLE siRNAs were purchased from HanYi Biosciences (Guangzhou, China). The siRNA oligonucleotides were transfected using Lipofectamine RNAiMAX (Invitrogen) according to the manufacturer’s instructions. The siRNA target sequences used in this research were: siSQLE-1: 5′- GCACCACAGTTTAAAGCAAAT-3′; siSQLE-2: 5′- GCTCAGGCTCTTTATGAATTA-3′. Transfection efficiency was confirmed by reverse-transcription quantitative PCR (RT-qPCR) and immunoblotting with the indicated antibodies.

For stable overexpression of SQLE, the SQLE coding sequence was cloned into a lentiviral vector pCDH-EF1-copGFP-T2A-Puro (Addgene #72263). For stable knockdown, short hairpin RNA (shRNA) sequences targeting SQLE designed based on the siRNA sequences, were cloned into pLKO.1-TRC (Addgene #10879), with scramble shRNA (Addgene #1864) as negative control. Lentiviruses were produced by cotransfecting HEK293T cells with the lentiviral vector, psPAX2 (Addgene #8454) and pCMV VSV-G (Addgene #8454) using Lipofectamine 3000. The supernatants containing lentivirus were collected after 48-hour transfection. Filtered supernatants were used to infect SNU449 cells in the presence of 8 µg/mL polybrene. Infected cells were selected with 3 µg/mL puromycin.

### RNA extraction and real-time PCR analysis

Total RNA was extracted by using the EZ-press RNA Purification Kit (EZBioscience). Reverse transcription was performed by 4×Reverse Transcription Master Mix (EZBioscience), and real-time PCR was conducted using the 2×SYBR Green qPCR Master Mix (EZBioscience) according to manufacturer’s instructions. Data were analyzed using the 2−ΔΔCt method. The primer sequences used in this study are listed in online supplemental table 2.

### RNA-seq and data analysis

Total RNA was extracted from human CD8^+^ T cells co-cultured with DMSO or 10 µM terbinafine-treated SNU449 cells using TRIzol (Invitrogen) and assessed with an Agilent 2100 Bioanalyzer (Agilent Technologies) and Qubit Fluorometer (Invitrogen). RNA samples meeting the following criteria were used in subsequent experiments: RNA integrity number >7.0 and 28S:18S ratio >1.8. RNA-seq libraries were generated and sequenced by Beijing Genomics Institute (Shenzhen, China). RNA-seq reads were aligned to the human genome (hg19) using TopHat2, and the number of reads mapped to each gene was calculated by HTseq. Differentially expressed genes (DEGs) were identified using edgeR based on a negative binomial distribution. Gene Set Enrichment Analysis (GSEA) was conducted to determine whether DEGs were related to one phenotype or signaling pathway.

### Luciferase reporter assay

The promoter fragment of CYP27A1 was cloned upstream of the firefly luciferase reporter in a pGL3-Basic vector. Indicated cells were co-transfected with the pGL3-promoter plasmid and a pRL-SV40 Renilla luciferase reporter plasmid in the presence or absence of 27-hydroxycholesterol (27HC) or cholesterol. Firefly and Renilla luciferase activities were measured using the Dual-Luciferase Reporter Assay System (Promega). Firefly luciferase activity was normalized to Renilla luciferase activity and is presented as relative luciferase activity.

### Immunofluorescent staining

Jurkat cells were sedimented onto poly-L-lysine-coated confocal dishes and fixed with 4% polyformaldehyde for 15 min. Fixed cells were permeabilized and blocked with phosphate-buffered saline (PBS) containing 1% Triton X-100 and 5% bovine serum albumin (BSA) for 30 min. Cells were then incubated overnight at 4°C with antibodies to *SREBP2* (Novus) and *LXRα* (Abcam). After three washes with PBST, specimens were incubated with Alexa Fluor-conjugated secondary antibodies and counterstained with 2-(4-Amidinophenyl)-6-indolecarbamidine dihydrochloride (DAPI). Images were captured by laser-scanning confocal microscopy (LSM 880, Zeiss) at ×400 magnification, with at least five fields per sample. Immunofluorescence images were analyzed using ZEN software (Zeiss).

### Western blot

Cells were washed twice with PBS and lysed with RIPA buffer (Beyotime) supplemented with phenylmethylsulfonyl fluoride (Beyotime), and a phosphatase inhibitor cocktail (Beyotime). Total proteins were resolved by sodium dodecyl sulfate - polyacrylamide gel electrophoresis (SDS-PAGE) and transferred onto polyvinylidene difluoride membranes (Millipore). Membranes were blocked with 5% BSA and incubated overnight at 4°C with primary antibodies against SQLE, ERK, p-ERK and HSP70 or β-ACTIN. Following incubation with horseradish peroxidase (HRP)-conjugated secondary antibodies, protein bands were visualized using an ECL Plus Western Blotting Substrate (Thermo Fisher Scientific) on a Bio-Rad ChemiDoc XRS+Imaging System.

### Immunostaining and image analysis

Paraffin-embedded blocks containing the advancing edges of tumor HCC tissue were used for tissue microarray (TMA) construction. TMAs were then cut into 4 µm sections and subjected to immunohistochemistry (IHC) and multiplex IHC as previously described.^21^ Details of antibodies used are provided in online supplemental table 3. Slides were scanned using Leica Aperio Versa 200 or KFBIO Digital Pathology Scanner (KF-PRO-020). The digital images were analyzed using HALO image analysis platform or an open-source software QuPath.^22^

### Dataset processing

Gene expression data for HCC were obtained from publicly available datasets. Expression matrices and clinical information for The Cancer Genome Atlas (TCGA)-liver hepatocellular carcinoma (LIHC) cohort were obtained from TCGA via the UCSC Xena browser. Gene expression data for GSE57957 and GSE62232 were downloaded from the Gene Expression Omnibus. Gene sets associated with cholesterol metabolism were retrieved from the Molecular Signature Database (MSigDB) with the keyword “cholesterol” (online supplemental table 4). Differential expression analysis was performed using the *limma* R package. For prognostic analysis, each gene was categorized based on the median expression value. Patients in the TCGA-LIHC cohort were stratified into high and low expression groups according to the median cut-off, and Kaplan-Meier survival analysis was performed to assess the impact of gene expression on patient survival (online supplemental tables 5,6). Tumor immune infiltration in HCC samples was estimated using the TIMER database (https://cistrome.shinyapps.io/timer/), and the IOBR R package was used for the analysis of immune infiltration in the TCGA-LIHC cohort.

Single-cell RNA sequencing data of HCC samples were obtained from the Genome Sequence Archive (accession number: PRJCA007744, https://ngdc.cncb.ac.cn). Samples annotated as HCC tumors were selected for analysis based on clinical metadata. Raw expression matrices were processed using the Seurat package (V.4.0) in R. After filtering low-quality cells, normalization and scaling were performed. Principal component analysis followed by uniform manifold approximation and projection was applied for dimensionality reduction. Cell clustering was conducted using the Louvain algorithm. Cell type identities were assigned based on canonical lineage markers. SQLE expression was visualized using density maps across identified clusters.

### Tumor inoculation and treatments

For in vivo tumor growth experiments, 1×10^6^ Hepa 1–6 cells were subcutaneously inoculated into the posterior flanks of mice. An HCC somatic mouse model was generated using hydrodynamic tail vein injections (HDTVi). Briefly, 10 µg PT3-EF1α-Myc, 10 µg PX330-sgTrp53, and 5 µg SB-13 were suspended in sterile Ringer’s solution in a volume equal to 10% of the body weight and injected via the tail within 5–7 s. After tumor cell inoculation, mice were randomly assigned to different groups. Terbinafine was given by oral gavage at a dose of 50 mg/kg per mouse, starting on day 5 when tumors became palpable, and continued daily for the duration of the experiment. Anti-mouse PD-1 or isotype control antibodies were administered intraperitoneally at a dose of 50 mg per mouse, starting on day 5 after tumor inoculation and continuing every 3 days for the duration of the experiment. For CD8^+^ T cell depletion, anti-mouse CD8 or control IgG antibodies were administered intraperitoneally at a dose of 200 mg per mouse on days 7, 10, 13, and 16 after tumor inoculation. Animals were euthanized at specific time points or when symptoms of tumorigenesis such as abdominal enlargement were evident. Tumor volumes were measured along the maximum axis (L) and the right-angle diameter to that axis (W) and calculated as follows: tumor volume = (L^3^ × W^2^)/2. Mice were sacrificed according to institutional ethical guidelines. Mouse tumors were washed with ice-cold PBS and cut into small pieces, followed by enzymatic digestion with collagenase D and DNase I (Roche) using a gentle MACS Tissue Dissociator (Miltenyi). Cells were filtered through a 70 mm filter and suspended in PBS. The cell suspension was loaded onto 40% Percoll (Cytiva) and centrifuged at 900× g for 30 min at 4°C. Cells were collected from the sediment, washed with PBS, treated with RBC Lysis Buffer (BioLegend) to remove red blood cells, and then subjected to flow cytometry analysis.

### Statistical analysis

Statistical analyses were performed using R software, GraphPad Prism software, and SPSS Statistics. Cumulative survival time was calculated by the Kaplan-Meier method, followed by statistical evaluation with the log-rank test. Univariate and multivariate analyses for OS were performed using the Cox proportional hazards model. A two-tailed Student’s t-test was applied for comparison between the two groups. Multiple comparisons were analyzed by one-way analysis of variance. Associations between stratified variables were evaluated using χ² tests (or Fisher’s exact test, when appropriate). Data are expressed as mean±SEM. P values less than 0.05 were considered statistically significant.

## Results

### High tumorous SQLE expression correlates with poor prognosis and reduced CD8^+^ T cell infiltration in patients with HCC

**Figure 1:**
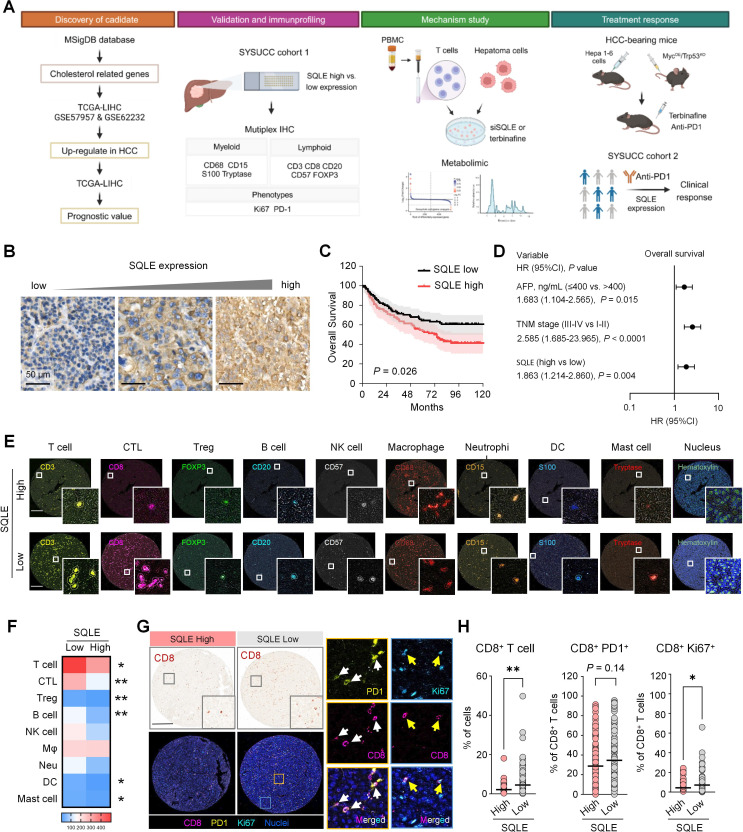
Association of SQLE expression with prognosis and immune infiltration in HCC. (**A**) Study design schematic illustrating the workflow of this study. (**B**) Representative images of SQLE immunostaining in HCC tissue sections. Scale bar: 50 µm. (**C**) Kaplan-Meier survival curve for OS of patients with HCC in the SYSUCC cohort 1. Patients were divided into two groups based on the median H-score of SQLE expression, as determined by IHC. (**D**) Forest plot depicting the association between OS and the prognostic variables. A multivariate Cox proportional hazard regression model was applied, incorporating variables that exhibited a significant association with OS in univariate analysis. (**E**) Representative multiplex IHC staining and positive cell segmentation of immune cells in SQLE low-expressing and high-expressing HCC tissue. Paraffin-embedded HCC tumor sections were subjected to multiplex IHC using antibodies against CD3, CD8, FOXP3, CD20, CD57, CD68, CD15, S100, and Tryptase. Insets show detailed images of the indicated regions. Scale bar, 200 µm. (**F**) Quantitative assessment of immune cell densities in HCC tissue with low versus high SQLE expression. Mean values of immune cell densities were compared using Student’s t-test and presented as a heatmap. (**G**) Representative multiplex IHC staining in HCC samples with low and high SQLE expression. Paraffin-embedded HCC tumor sections were subjected to multiplex IHC using anti-CD8, PD-1 and Ki-67. Micrographs of the indicated region are shown. White arrow indicates CD8^+^ PD1^+^ cell, yellow arrow indicates CD8^+^ Ki67^+^ cell. Scale bar, 250 µm. (**H**) Proportion of the immune cells in HCC tissues, segregated by SQLE expression levels. *p < 0.05, **p < 0.01. AFP, alpha fetoprotein; CTL, cytotoxic T lymphocyte; DC, dendritic cell; HCC, hepatocellular carcinoma; IHC, immunohistochemistry; LIHC, liver hepatocellular carcinoma; Mφ, macrophage; MSigDB, Molecular Signature Database; Neu, neutrophils; NK, natural killer; OS, overall survival; PBMC, peripheral blood mononuclear cell; PD-1, programmed cell death protein-1; SQLE, squalene epoxidase; SYSUCC, Sun Yat-sen University Cancer Center; TCGA, The Cancer Genome Atlas; TNM, tumor, node, metastases; Treg, regulatory T cell.

To identify potential therapeutic targets based on the aberrant cholesterol metabolism in HCC, we focused on gene sets associated with cholesterol metabolism, which were retrieved from MsigDB (online supplemental table 4). We compared the expression levels of these genes between tumor and matched non-tumor tissues using three public datasets of patients with HCC (GSE57957, GSE62232, and TCGA-LIHC), and further assessed their prognostic significance in the TCGA-LIHC dataset (figure 1A). Among the candidate genes, SQLE emerged as a key hub gene, exhibiting significantly higher expression in tumor tissues and serving as a potential prognostic biomarker for patient survival (online supplemental figure 1A,B and tables 5,6). Analysis of the single-cell RNA-seq dataset (PRJCA007744) further revealed that SQLE expression was predominantly restricted to epithelial tumor cells, reinforcing its tumor cell-intrinsic expression pattern (online supplemental figure 1C)**.**

To validate this finding, we performed IHC staining for SQLE in an independent cohort of 196 patients with HCC from SYSUCC (figure 1B). Tumorous SQLE expression was quantified using the H-score, and the results showed that high SQLE expression was associated with short OS of patients (figure 1C). Multivariate analysis revealed that SQLE expression was an independent prognostic factor for OS in these patients (figure 1D and online supplemental table 7**)**.

Next, we characterized the immune landscape in HCC tumors with different levels of SQLE expression by multiplex IHC staining, which allowed the spatially resolved identification of nine immune cell types within the TME (figure 1E). Correlation analysis revealed that SQLE expression was associated with the infiltrations of multiple immune cell types (figure 1F), indicating that aberrant upregulation of SQLE may alter the TME. Notably, CD8^+^ T cells, which are crucial for immunotherapy, were significantly reduced in tumors with high SQLE expression in both the SYSUCC and TCGA-LIHC cohorts (figure 1G and H, online supplemental figure 1D). Furthermore, lower proliferation and less PD-1 expression were found in CD8^+^ T cells of SQLE-high tumors, indicating impaired T cell activation.

Collectively, these findings suggest that upregulated tumorous SQLE expression correlates with poor clinical outcome, and reduced CD8^+^ T cell infiltration and activation, highlighting SQLE as a potential modulator of the TME in HCC.

### Suppression of tumorous SQLE enhances the effector phenotype of CD8^+^ T **cells**

**Figure 2:**
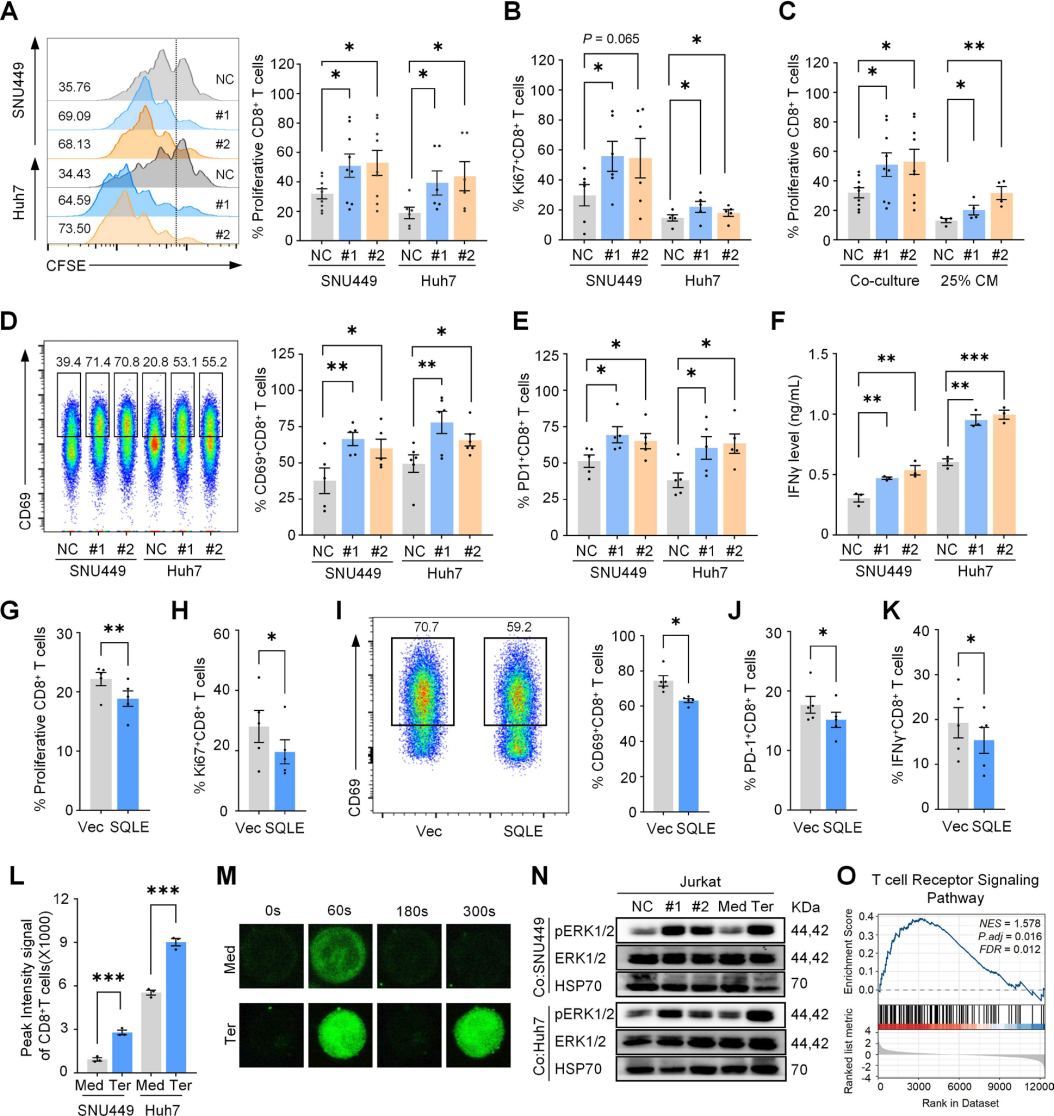
Knockdown of tumorous SQLE enhances the activation of human CD8^+^ T cells. (**A**) The proportion of CFSE-labeled human CD8^+^ T cells was measured after 24 hours of stimulation with anti-CD3/28 and subsequent co-culture with scramble (NC) or si-SQLE HCC cells for 72 hours. (**B**) The proportion of Ki67^+^ CD8^+^ T cells was assessed by flow cytometry in each co-culture group. (**C**) Flow cytometry assessed the proportion of CFSE-labeled human CD8^+^ T cells co-cultured with genetically edited HCC cells or with conditioned medium (CM). (**D**) The proportion of CD8^+^ T cells expressing CD69 was measured by flow cytometry in the co-culture system. (**E**) Flow cytometric analysis determined the proportion of PD-1^+^ CD8^+^ T cells in the co-culture system. (**F**) The IFNγ levels in the CM collected from the co-culture system were determined by ELISA. (**G–K**) CD8^+^ T cells were cultured with CM from vector (Vec) or SQLE-overexpressing SNU449 cells. Flow cytometry was used to determine the proportions of CFSE^+^ (**G**) and Ki67^+^ (**H**) CD8^+^ T cells. Representative plots and quantification are shown for CD69^+^ CD8^+^ T cells (**I**), while PD1^+^ (**J**) and IFNγ^+^ (**K**) CD8^+^ T cell proportions are also indicated. (**L**) Flow cytometry determined the peak fluorescence intensities in human CD8^+^ T co-cultured with SNU449 cells treated with either terbinafine or control medium. (**M**) Confocal microscopy captured Fluo-4AM fluorescence in CD8^+^ T cells. The peak fluorescence intensities were recorded after stimulation with ionomycin (100 ng/mL) for 60 s, followed by treatment with 2 mM CaCl_2_ for 300 s. (**N**) Western blot of phosphorylated and total ERK1/2 in Jurkat cells co-cultured with SNU449 cells in the presence or absence of terbinafine. (**O**) Enrichment of T cell receptor signaling pathway identified from the bulk transcriptomic comparison of human CD8^+^ T cells co-cultured with SNU449 cells in the presence or absence of terbinafine. *p < 0.05, **p < 0.01, ***p < 0.001. CFSE, carboxyfluorescein succinimidyl amino ester. IFNγ, interferon-gamma; Med, control medium; PD-1, programmed cell death protein-1; SQLE, squalene epoxidase; Ter, terbinafine.

To investigate the impact of tumorous SQLE on CD8^+^ T cells, we first constructed SQLE HCC cell lines (online supplemental figure 2A,B), and co-cultured them with human peripheral blood-derived CD8^+^ T cells, which were simultaneously stimulated with anti-CD3/CD28. Silencing SQLE in HCC cells significantly enhanced the proliferation of co-cultured CD8^+^ T cells compared with those with control HCC cells (figure 2A and B). To determine whether this enhanced proliferation was dependent on direct contact between tumor cells and CD8^+^ T cells, we collected CM from SQLE-inhibited HCC cells and transferred it to the CD8^+^ T cell culture system. Enhanced CD8^+^ T cell proliferation was also observed, indicating that the effect was mainly mediated by soluble factor(s) in the CM (figure 2C).

The functional status of CD8^+^ T cells co-cultured with HCC cells was further determined, and the results showed that CD8^+^ T cells co-cultured with SQLE-inhibited HCC cells exhibited stronger activation compared with their counterpart, as examined by CD69, PD1 and IFNγ expression (figure 2D–F). Aforementioned co-culture experiments were repeated where cells were treated by SQLE inhibitor terbinafine, in a dose that did not affect the proliferation of HCC or CD8^+^ T cells (online supplemental figure 2C,D). Higher levels of CD8^+^ T cell proliferation and activation were also observed in the terbinafine-treated group (online supplemental figure 2E–I).

To further confirm the role of SQLE in regulating T cell function, SQLE-overexpressing cells were constructed (online supplemental figure 2J), and their culture supernatants were used to treat CD8^+^ T cells. In contrast to the knockdown model, SQLE overexpression significantly suppressed both CD8^+^ T cell proliferation and activation (figure 2G–K). To validate the specificity of this effect, SQLE-targeting shRNA was introduced into the SQLE-overexpressing cells (online supplemental figure 2J). In this model, CD8^+^ T cell proliferation and activation were significantly restored compared with the overexpression-only condition (online supplemental figure 2K,L).

On T cell receptor (TCR) engagement with the peptide-major histocompatibility complex, T cell activation is initiated by proximal signaling and downstream signaling events such as calcium influx and MAPK signaling.^23^ As expected, SQLE inhibition in HCC cells enhanced the amount and speed of calcium influx into CD8^+^ T cells (figure 2L and M), as well as increased phosphorylation of ERK (figure 2N). Treatment with a specific ERK inhibitor (SCH772984) attenuated the SQLE-mediated enhancement of T cell proliferation (online supplemental figure 2M)**.** Moreover, the transcriptomic data of CD8^+^ T cells co-cultured with either si-SQLE or si-NC HCC cells was analyzed, and GSEA revealed that the TCR signaling pathway was altered in CD8^+^ T cells when co-cultured with SQLE-silenced HCC cells (figure 2O).

Taken together, these results suggest suppression of SQLE in HCC cells rejuvenates CD8^+^ T cells and skews the local immune response toward an antitumor phenotype.

### SQLE orchestrates oxysterols production in HCC cells by modulating cholesterol metabolism

**Figure 3:**
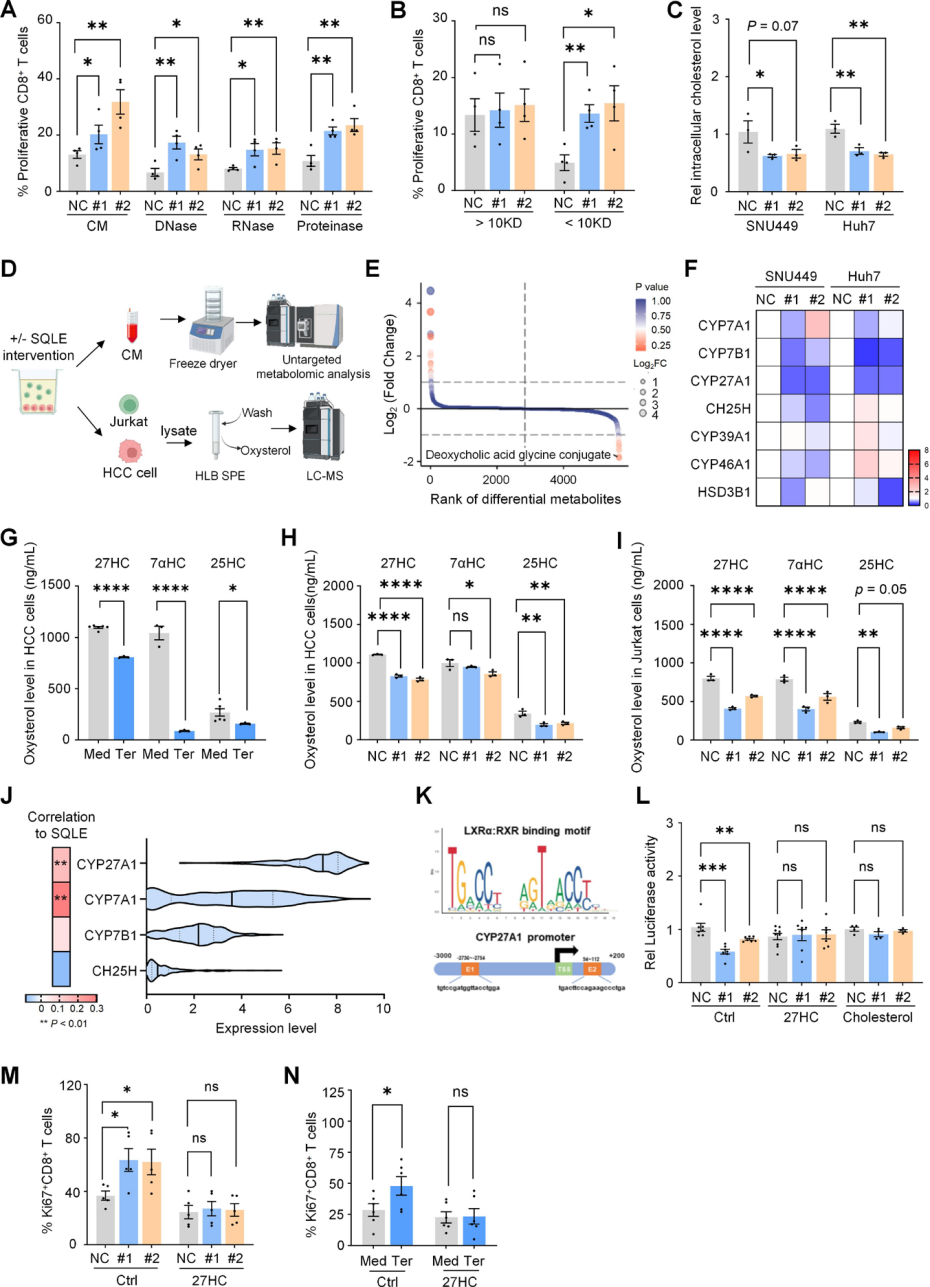
SQLE regulates tumor-derived oxysterols by modulating cholesterol metabolism. (**A**) Human CD8^+^ T cells labeled with CFSE were cultured for 3 days with CM collected from SNU449 cells treated with scramble (NC) or si-SQLE, which was pretreated with DNase, RNase, or proteinase. (**B**) CFSE-labeled human CD8^+^ T cells were cultured for 3 days with low (<10 kDa) and high (>10 kDa) molecular weight fractions of CM from SNU449 cells treated with scramble or si-SQLE. (**C**) Intracellular cholesterol levels were measured in cell lysates from HCC cells treated with si-NC or si-SQLE. (**D**) Schematics illustrate the LC/MS analysis of HCC cells (terbinafine-treated or control medium-treated) and Jurkat cells in a co-culture system, with CM analyzed. (**E**) Untargeted metabolomics data show different metabolites in CM from the co-culture system with terbinafine treatment compared with the control medium. (**F**) Quantitative PCR analysis measured cholesterol hydroxylase expression in SNU449 cells treated with scramble or si-SQLE. (**G**) The levels of indicated oxysterols in SNU449 cells treated with terbinafine (Ter) or untreated (Med) were quantified by LC/MS. (**H**) The levels of indicated oxysterols in scramble or SQLE-silenced SNU449 cells were quantified. (**I**) Jurkat cells were co-cultured with scramble or si-SQLE SNU449 cells for 3 days, and intracellular oxysterols were quantified. (**J**) The correlation between cholesterol hydroxylase and SQLE expression was shown in the heatmap, and the mRNA expression levels of cholesterol hydroxylase in the TCGA-LIHC dataset were shown in the violin plot. (**K**) Prediction of LXR*α* binding site on the CYP27A1 promoter region using the JASPAR website. (**L**) Relative luciferase activity of the CYP27A1 promoter in si-SQLE SNU449 cells, in the presence or absence of 27HC or cholesterol. (**M**) Flow cytometry analysis of Ki67^+^ CD8^+^ T cells co-cultured with scramble or si-SQLE HCC cells, in the presence or absence of 27HC (2 µM). (**N**) Flow cytometry analysis of Ki67^+^ CD8^+^ T cells co-cultured with HCC cells treated by terbinafine (Ter) or untreated (Med), in the presence or absence of 27HC (2 µM). *p < 0.05, **p < 0.01,***p < 0.001, ****p < 0.0001. CFSE, carboxyfluorescein succinimidyl amino ester; CM, culture medium; FC, fold change; HCC, hepatocellular carcinoma; HLB, hydrophilic-lipophilic balanced; LC-MS, liquid chromatography coupled with high-resolution mass spectrometry; LIHC, liver hepatocellular carcinoma; mRNA, messenger RNA; ns, not significant; SPE, solid-phase extraction; SQLE, squalene epoxidase; TCGA, The Cancer Genome Atlas.

To identify the soluble factor(s) secreted by HCC cells with proficient SQLE that may influence CD8^+^ T cell activation, we first examined the general species and molecular weight of potential factor(s) using specific digestion enzymes and molecular filters. We found that only fractions with a molecular weight of less than 10 kDa, which were neither proteins nor nucleotides, could contribute to SQLE-mediated CD8^+^ T cell activation (figure 3A and B).

Given that SQLE functions as a key enzyme in cholesterol biosynthesis, and exogenous cholesterol has been demonstrated to play a critical role in modulating T cell function, we investigated the impact of SQLE deficiency on cholesterol metabolism within the TME and its potential modulation of CD8^+^ T cell function. However, despite a reduction of intracellular cholesterol in HCC cells treated with si-SQLE or terbinafine, no obvious change in cholesterol level was detected in the CM on SQLE suppression, but increased cholesterol level in CD8^+^ T cells was observed (figure 3C and online supplemental figure 3A–C).

To identify the immunomodulatory substances and regulatory mechanisms of SQLE, we performed untargeted metabolomic analysis on the supernatants of HCC cells treated with or without terbinafine (figure 3D). Among the differential metabolites identified in the terbinafine-treated group, we observed a significant downregulation of the deoxycholic acid glycine conjugate, an intermediate in bile acid metabolism, suggesting that the metabolic flux from cholesterol to bile acids may be restricted by SQLE inhibition. This finding aligns with qPCR data showing reduced expression of hydroxylase enzymes involved in converting cholesterol to oxysterols and subsequently to bile acids (figure 3E and F).

We then measured the levels of major oxysterols, including 7α-hydroxycholesterol (7αHC), 25-hydroxycholesterol (25HC), and 27HC, excluding 24-hydroxycholesterol, which mainly exists in the brain.^9 24^ These oxysterols were enriched and purified from cell lysates in the co-culture system through HLB SPE, followed by quantitative analysis using LC-MS. As expected, SQLE inhibition significantly downregulated the levels of all three oxysterols in HCC cells, with 27HC showing the most prominent reduction in both SQLE-deficient HCC cells and co-cultured T cells (figure 3G–I and online supplemental figure 3D). Moreover, 27HC was catalytically produced by CYP27A1, whose expression was more abundant than other catalytic enzymes in HCC tissue and positively correlated to SQLE levels (figure 3J). Consistently, we also observed a downregulated expression of CYP27A1 in SQLE-silenced HCC cells (online supplemental figure 3E,F).

Although few studies have addressed the transcriptional regulation of CYP27A1, previous research has shown that the expression of another similar hydroxylase, CYP7A1, is stimulated by cholesterol through the activation of the transcription factor *LXRα*.^25^ Intriguingly, in silico predictions using JASPER also indicated that *LXRα* may bind to the promoter region of CYP27A1 (figure 3K). To validate this hypothesis, we performed a luciferase assay and found that SQLE inhibition in HCC cells suppressed *LXRα* activity, which could be rescued by the addition of cholesterol or 27HC (figure 3L).

To further confirm the impact of tumor-derived 27HC on CD8^+^ T cell function, we exogenously added 27HC in conditioned medium derived from SQLE-silenced HCC cells. Supplementation with 27HC attenuated the increased CD8^+^ T cell proliferation induced by SQLE inhibition in HCC cells **(**figure 3M and N**)**. Taken together, these findings implicate that SQLE orchestrates the regulation of 27HC production in HCC cells by modulating cholesterol metabolism.

### SQLE modulates *SREBP2*-dependent cholesterol metabolism in CD8^+^ T cells via 27-hydroxycholesterol

**Figure 4:**
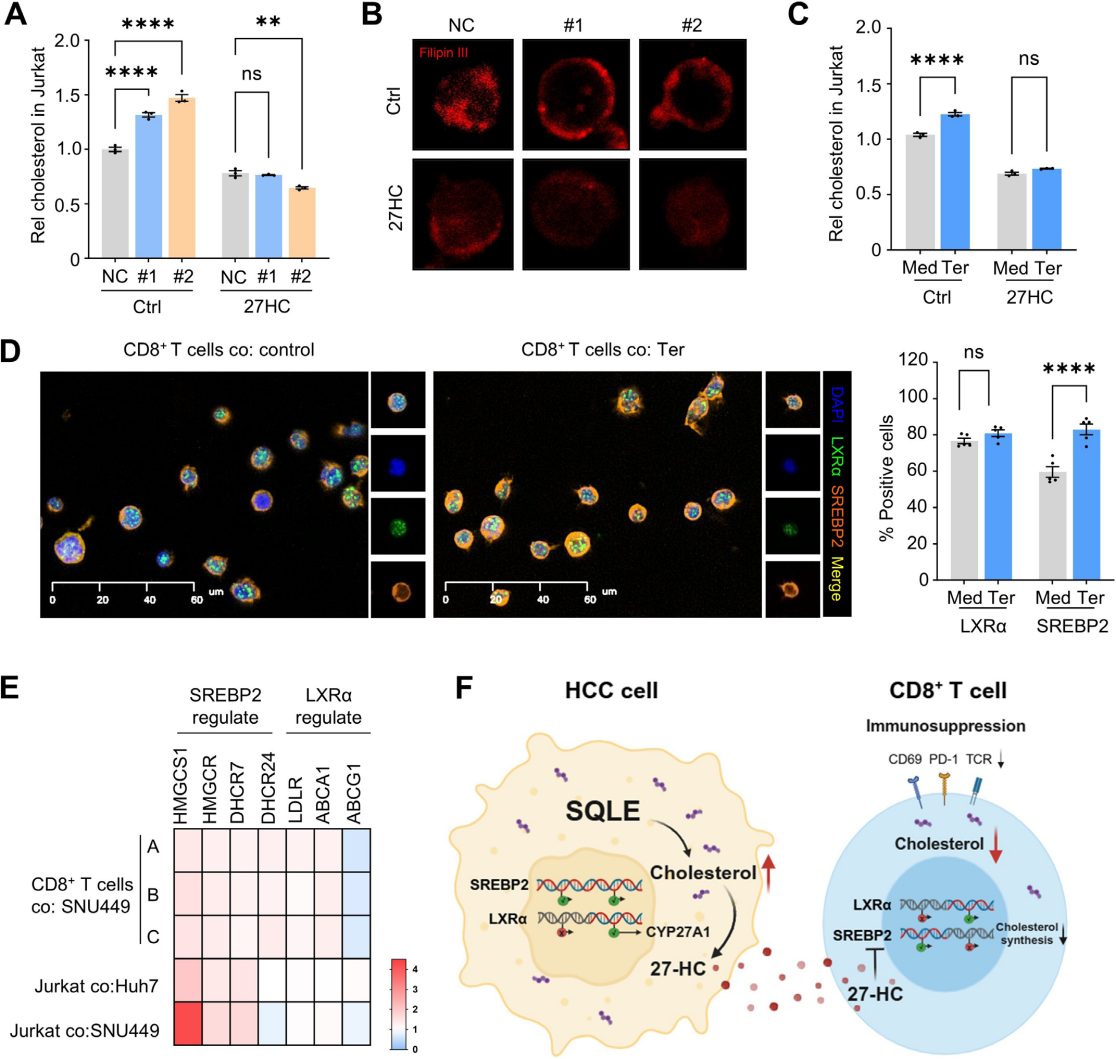
Inhibition of tumorous SQLE upregulates cholesterol levels in CD8^+^ T cells. (**A**) Cholesterol levels in Jurkat cells were determined in scramble or si-SQLE group, in the presence or absence of 27HC. (**B**) Confocal images of Filipin III staining free cholesterol of cell membranes in Jurkat cells are shown. (**C**) Cholesterol levels were determined in Jurkat cells co-cultured with SNU449 cells treated by terbinafine (Ter) or vehicle control (Med). (**D**) Human CD8^+^ T cells were co-cultured with SNU449 cells in the presence or absence of terbinafine. Expression of *LXRα* and *SREBP2* in CD8^+^ cells was visualized and quantified by immunofluorescence. (**E**) Human CD8^+^ T cells or Jurkat cells were co-cultured with scramble or si-SQLE-treated SNU449 cells. Gene expression was assessed by qPCR analysis, and fold changes in expression are shown in a heatmap. (**F**) Schematic representation of the hypothesized mechanism by which tumor-derived SQLE regulates cholesterol metabolism in both HCC cells and CD8^+^ T cells. **p < 0.01; ****p < 0.0001. HCC, hepatocellular carcinoma; ns, not significant; PD-1, programmed cell death protein-1; qPCR, quantitative PCR; SQLE, squalene epoxidase; TCR, T cell receptor.

Studies have established the essential role of cholesterol in T cell immunocompetence, yet within the TME, T cells exhibit reduced cholesterol levels relative to other immune cells, reflecting a distinct metabolic profile. ^7^ To explore whether SQLE influences this process, we performed RNA-seq of CD8^+^ T cells from the co-culture system. GSEA analysis revealed significant enrichment of the cholesterol biosynthesis pathway when SQLE was inhibited in HCC cells (online supplemental figure 4A and 4B). Consistently, both total and free cholesterol levels in CD8^+^ T cells were elevated in the SQLE-deficient group, whereas 27HC treatment hindered this increase (figure 4A–C).

To further test whether SQLE regulates immune function via modulating cholesterol metabolism in CD8^+^ T cells, we supplemented exogenous cholesterol in CD8^+^ T cells cultured with CM from sh-SQLE or control HCC cells. This supplementation abolished the enhanced proliferation observed in CD8^+^ T cells exposed to the SQLE-silenced group (online supplemental figure 4C), indicating that SQLE influences CD8^+^ T cell function through modulating intracellular cholesterol levels.

As intracellular cholesterol homeostasis is tightly regulated by the balance of transcriptional factors *SREBP2* and *LXRα*, with 27HC serving as a co-factor for enhancing the transcriptional activity of *LXRα*, as well as preventing *SREBP2*-mediated cholesterol accumulation.^26^ Indeed, we observed elevated nuclear *SREBP2* signals and its downstream genes expression in T cells co-cultured with SQLE-deficient HCC cells (figure 4D and E). These findings suggest that reduced 27HC levels in SQLE-deficient HCC cells promote *SREBP2*-dependent cholesterol biosynthesis signaling in CD8^+^ T cells, restoring T cell cholesterol level and supporting activation.

Taken together, these findings suggest that suppression of tumorous SQLE reprograms cholesterol metabolism in both CD8^+^ T cells and HCC cells, contributing to the establishment of an immunosuppressive TME in HCC (figure 4F).

### SQLE inhibitor elicits CD8^+^ T cell-mediated antitumor response

**Figure 5:**
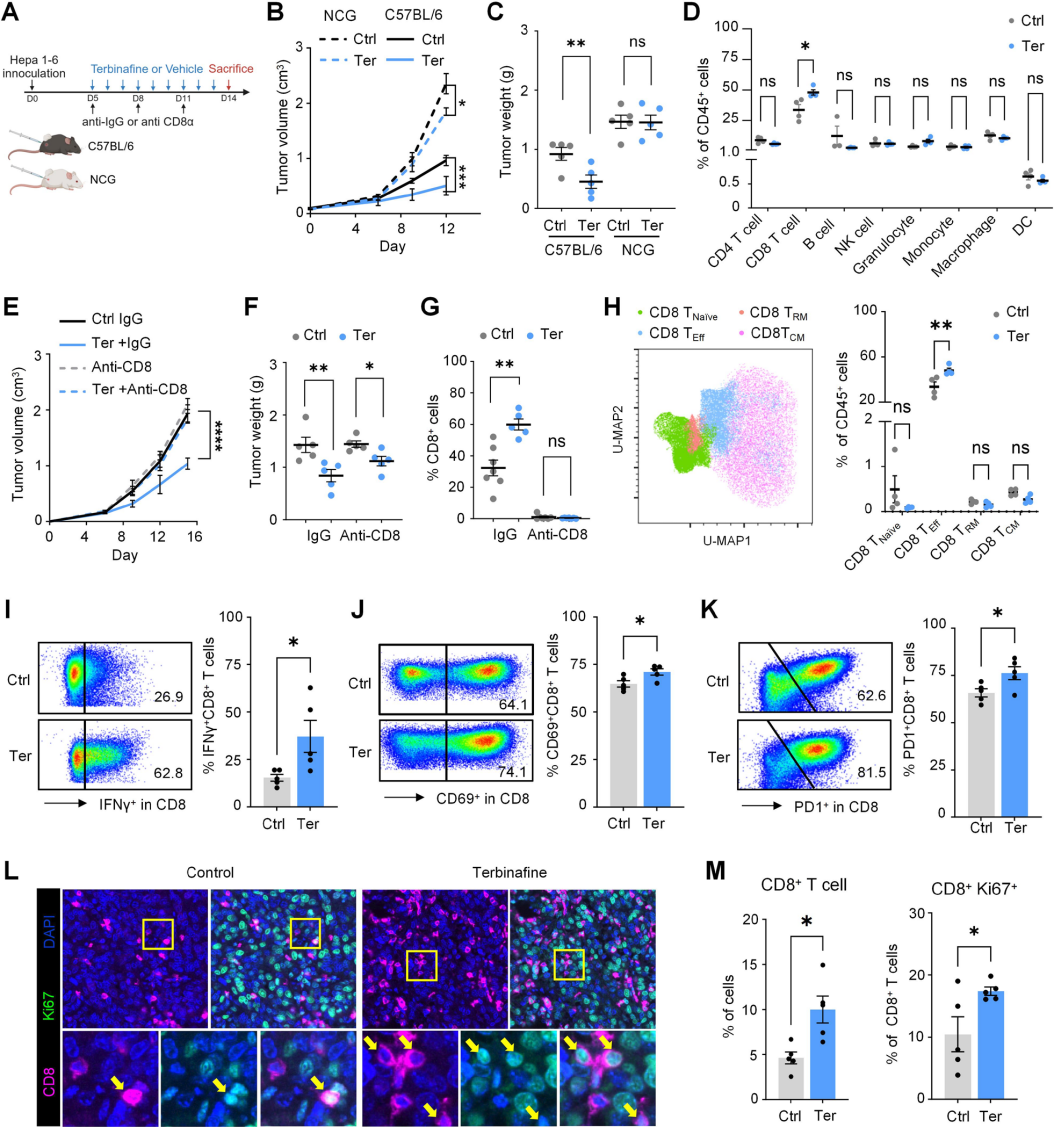
Terbinafine boosts CD8^+^ T cell-mediated antitumor response in HCC mouse models. (**A**) Schematic showing the experimental design of terbinafine treatment in mice bearing subcutaneous murine HCC tumors (n=5). (**B**) Growth curve of C57BL/6 mice or NCG mice treated with or without terbinafine. (**C**) Tumor weight at endpoint of C57BL/6 mice or NCG mice treated with or without terbinafine. (**D**) Flow cytometric analysis of major immune subsets in mice bearing Hepa 1–6 subcutaneous tumors, administered with terbinafine or vehicle. (**E**) Growth curve in C57BL/6 mice bearing subcutaneous HCC treated with terbinafine or vehicle, with or without CD8 depletion. (**F**) Tumor weight at the end point in C57BL/6 mice. (**G**) Flow cytometric analysis showing CD8^+^ T cell depletion in Hepa 1–6 tumors. (**H**) Uniform Manifold Approximation and Projection (UMAP) analysis showing subclustering of CD8^+^ T cells from control and terbinafine-treated groups. The percentages of different CD8^+^ T cell subsets are shown. (**I**) Representative flow cytometry plots and quantification of IFNγ^+^ CD8^+^ T cells from control and terbinafine-treated groups. (**J**) Representative flow cytometry plots and quantification of CD69^+^ CD8^+^ T cells from control and terbinafine-treated groups. (**K**) Representative flow cytometry plots and quantification of PD-1^+^ CD8^+^ T cells from control and terbinafine-treated groups. (**L**) Representative images of multiplex immunofluorescent staining of CD8 and Ki67 in Hepa 1–6 tumor sections. Yellow arrow indicates CD8^+^ Ki67^+^ T cells. (**M**) Quantification of multiplex immunofluorescent staining of CD8 and Ki67 in Hepa 1–6 tumor sections. *p < 0.05, **p < 0.01, ***p < 0.001. DAPI, 2-(4-Amidinophenyl)-6-indolecarbamidine dihydrochloride. DC, dendritic cell; HCC, hepatocellular carcinoma; IFNγ, interferon-gamma; NK, natural killer; ns, not significant; PD-1, programmed cell death protein-1; T_CM_, central memory T cells; T_EFF_, effector T cells; Ter, terbinafine; T_RM_, resident memory T cells.

In light of the above results, we investigated whether SQLE inhibition could be an effective therapeutic strategy in HCC mouse models (figure 5A). SQLE inhibitor, terbinafine, delayed tumor growth in both immunocompetent C57BL/6 mice and immunodeficient NCG mice transplanted with Hepa 1–6 tumor cells; however, the effect was weaker in the NCG mice, underscoring the importance of intact adaptive immunity in the efficacy of terbinafine treatment (figure 5B and C, online supplemental figure 5A). Immune landscape in the tumor was determined by flow cytometry, and the results showed that the proportion of CD8^+^ T cells was increased in terbinafine-treated mice (figure 5D and online supplemental figure 5B), coinciding with our previous in vitro findings. The necessity of CD8^+^ T cells in mediating the therapeutic response of terbinafine was further confirmed, as indicated that retarded tumor growth by terbinafine was not observed in mice with CD8^+^ T cell depletion (figure 5E–G and online supplemental figure 5A).

Subsequently, we performed spectral cytometry to investigate the functional state of CD8^+^ T cells, and found that the proportion of effector CD8^+^ T cells was significantly increased in terbinafine-treated mice (figure 5H and online supplemental figure 5C). Increased IFNγ, CD69 and PD1 expression on CD8^+^ T cells was observed in terbinafine-treated tumors (figure 5I–K). Immunofluorescent staining confirmed the increase of CD8^+^ T cell infiltration, as well as their enhanced proliferation (figure 5L and M). These findings indicate that terbinafine effectively enhanced the antitumor immunity mediated by CD8^+^ T cells.

### Synergistic antitumor effects of SQLE inhibition and anti-PD1 therapy in HCC models and its clinical implications

**Figure 6:**
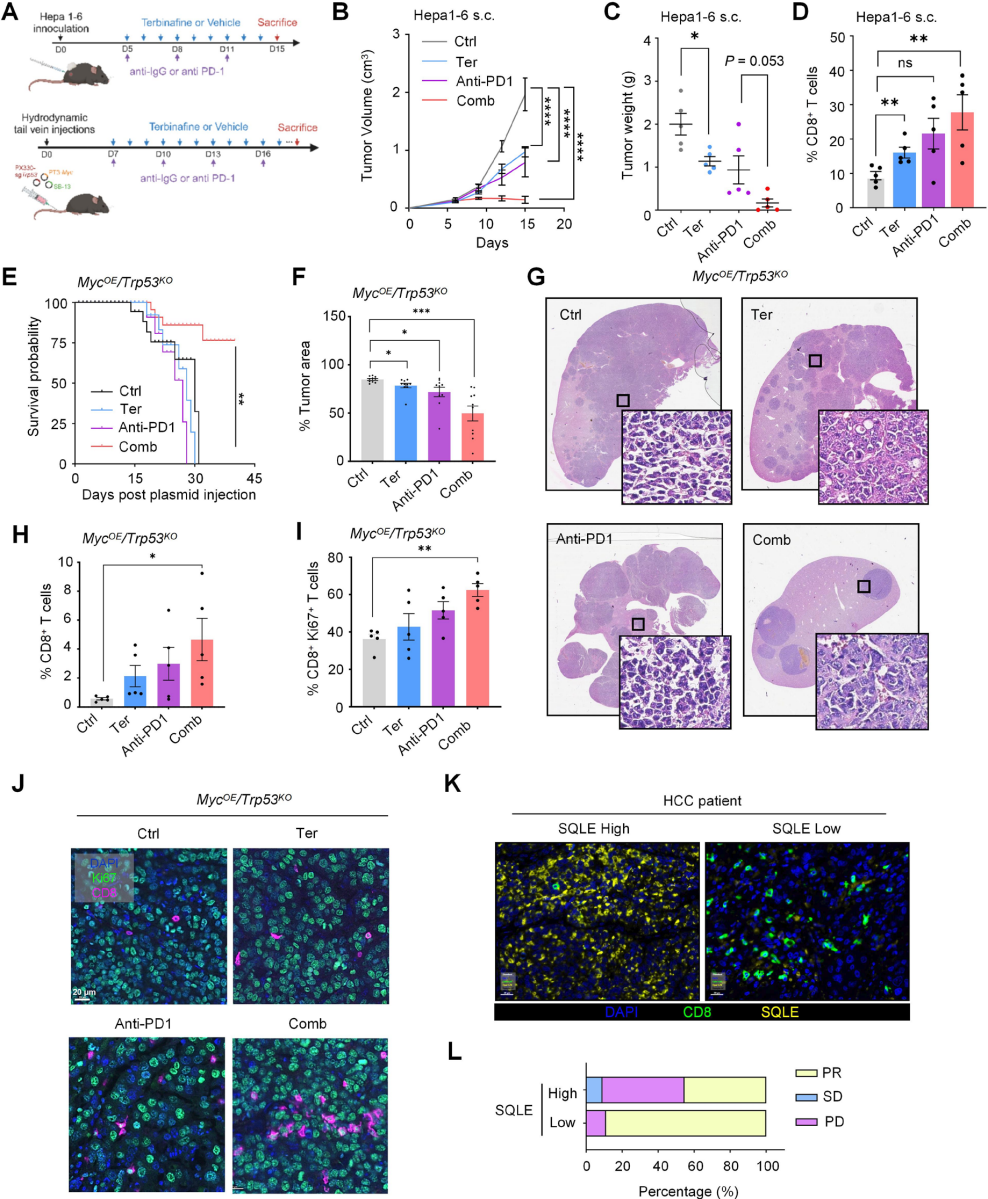
Terbinafine synergizes with anti-PD-1 therapy in HCC mouse models. (**A**) Experimental design showing the combined therapy of terbinafine and anti-PD-1, as well as the monotherapies (terbinafine or anti-PD-1) in two HCC mouse models (n=5). (**B**) Mice were subcutaneously injected with Hepa 1–6 cells to induce HCC tumors, and tumor growth kinetics of mice in each treatment group was compared. (**C**) Endpoint tumor weight in each treatment group. (**D**) Flow cytometric analysis showing the percentage of CD8^+^ T cells among viable CD45^+^ cells in each treatment group. (**E–J**) Mice were HDTV-injected to induce *Myc*^OE^/*Trp53^KO^* HCC tumors, and enrolled in treatments with anti-IgG, terbinafine, anti-PD1, or the combination of terbinafine and anti-PD1. (**E**) Survival analysis of *Myc*^OE^/*Trp53^KO^* mice in each treatment group is shown. (**F and G**) Representative H&E staining of tumor lesion from *Myc*^OE^/*Trp53^KO^* mice was displayed and quantified. (**H**) Quantification of the proportion of tumorous CD8^+^ T cells in each treatment group by flow cytometry. (**I and J**) Percentage of CD8^+^ Ki67^+^ T cells in tumor regions, with representative images shown. (**K**) Multiplex staining of CD8^+^ T cells and SQLE expression in tumor sections of patients with HCC receiving anti-PD-1 therapy. (**L**) Objective response rates of the patients grouped by SQLE expression levels are shown. *p < 0.05, **p < 0.01, ***p < 0.001, ****p < 0.0001. DAPI, 2-(4-Amidinophenyl)-6-indolecarbamidine dihydrochloride; HCC, hepatocellular carcinoma; HDTV, hydrodynamic tail vein; ns, not significant; PD, progressive disease; PR, partial response; PD-1, programmed cell death protein-1; s.c., subcutaneously; SD, stable disease; SQLE, squalene epoxidase; Ter, terbinafine.

We further evaluated the synergistic effect of terbinafine and anti-PD1 therapy. Hepa 1–6 flank tumor-bearing mice were treated with terbinafine and/or anti-PD1 for 2 weeks (figure 6A). Mice treated with anti-PD1 or terbinafine alone exhibited a moderate antitumor response, whereas the combinatory group showed significantly enhanced tumor regression, accompanied by a remarkable increase in tumor-infiltrating CD8^+^ T cells (figure 6B–D).

Furthermore, we recapitulated these findings in an HCC somatic mouse model (*Myc^OE^/Trp53^KO^*), which is characterized by low T cell infiltrations and resistance to anti-PD1 therapy. Mice receiving a combination treatment of terbinafine and anti-PD1 antibody exhibited prolonged survival and a reduction in tumor area (figure 6E–G). Consistently, we observed the upregulation of Ki67^+^ CD8^+^ T cells in the combination group (figure 6H–J). Collectively, these results suggest that terbinafine synergized with immunotherapy to enhance antitumor immunity in HCC mouse models.

To assess the clinical relevance of these findings, we analyzed a cohort of patients with HCC who received immune checkpoint blockade (ICB) therapies (anti-PD1). SQLE expression and CD8^+^ cell infiltrations were examined by multiplex immunostaining (figure 6K and L). The results showed that higher SQLE expression tended to be associated with a poorer response to ICB therapy in these patients with HCC (figure 6K and L). Taken together, these data suggest SQLE inhibition by terbinafine can improve the efficacy of ICB therapy, and that SQLE expression may serve as a novel biomarker for predicting response to immunotherapy in patients with HCC.

## Discussion

Cholesterol metabolism is crucial for tumor growth and progression, supplying essential lipids for membrane synthesis, energy production, and signaling of cancer cells.^27^ The reprogrammed cholesterol metabolism also profoundly impacts the TME, which plays a key role in driving tumor progression and resistance to therapy.^28^ In this study, we evaluated the expression of cholesterol metabolism-related genes in HCC and assessed their impact on clinical outcomes. We identified the cholesterol biosynthesis catalase SQLE as a potential therapeutic target. Functional studies demonstrated that SQLE-mediated alterations in cholesterol metabolism impair CD8^+^ T cell function, primarily through the secretion of the oxysterol 27HC. Moreover, inhibition of SQLE with the clinically available drug terbinafine enhanced the efficacy of anti-PD1 therapy, and SQLE expression was associated with better responses to anti-PD1 treatment in patients with HCC.

Although several cholesterol metabolic enzymes have been proposed as potential cancer targets and have led to the development of specific inhibitors, the clinical outcomes of these strategies have often failed to meet expectations.^26^ SQLE has previously been identified as a metabolic oncogene that enhances cancer proliferation and invasion across various cancer types. Interestingly, the correlation of tumorous SQLE expression with immune cell infiltration has also been reported in cancers such as pancreatic adenocarcinoma and breast cancer, suggesting a potential immune modulatory role of this molecule.^11 29 30^ In this study, we investigated the role of SQLE in shaping the immune landscape of human HCC. Using multiplex immunostaining, we characterized tumorous immune infiltration based on SQLE levels and found a correlation between SQLE expression and several immune cell types, including T cell, B cell, and dendritic cells. The most robust association was observed with CD8^+^ T cells, where SQLE expression showed a negative correlation and was also confirmed in the TCGA-LIHC dataset. Furthermore, we found that high SQLE expression was associated with reduced CD8^+^ T cell density and poor treatment responses in patients with HCC receiving anti-PD1 therapy. These data suggest that SQLE might serve as a valuable predictive biomarker for treatment efficacy, underscoring the need for further validation in larger patient cohorts undergoing immunotherapy.

Given that steatosis in hepatocytes may upregulate SQLE expression, we excluded patients with pathologically confirmed steatosis, and focused on HCC primarily related to HBV infection, which is predominant in China. ^16 31 32^ While previous studies have shown that SQLE promotes tumor progression and suppresses antitumor immunity in steatosis-driven HCC models, our study demonstrates that SQLE also impairs CD8^+^ T cell function in non-steatotic HCC.^16 33^ Specifically, SQLE inhibition enhanced CD8^+^ T cell activation without altering cholesterol levels in the tumor-conditioned medium, suggesting an alternative mechanism involving oxysterol-mediated cholesterol restriction. High SQLE expression in HCC cells not only supports their own cholesterol requirements but also generates oxidized sterol derivatives that indirectly impair T cell function. Thus, inhibition of SQLE may increase T cell cholesterol levels and restore their immune activity. These findings collectively support a broader role for SQLE in immune modulation beyond steatosis-associated HCC and reveal potential context-dependent differences in its immunoregulatory mechanisms.

The activation and functionality of tumor-infiltrating CD8^+^ T cells, influenced by tumor cells, are crucial for shaping the TME and determining responses to immunotherapy.^34^,^36^ To mimic the interplay in the TME, we constructed an in vitro co-culture system consisting of human CD8^+^ T cells and HCC cells, which were either genetically silenced or treated with an SQLE inhibitor. The results demonstrated that targeting SQLE effectively promoted CD8^+^ T cell proliferation and activation, likely due to enhanced cholesterol biosynthesis, which supports member fluidity and calcium uptake, facilitating TCR signaling and AMPK pathway activation. Consistently, CD8^+^ T cells in human HCC tumors with low SQLE expression exhibited higher levels of Ki67 and PD1. Importantly, inhibiting SQLE with terbinafine in HCC mouse models restored the antitumor activity of CD8^+^ cells.

Metabolic reprogramming in immune cells is often shaped by extracellular metabolites, which not only provide energy but also serve as signaling molecules facilitating communication between different cellular compartments. For example, lactate, succinate, and oxysterols can modulate immune cell activation, differentiation, and function, influencing the outcome of immune responses in the TME and other pathological settings.^37^,^39^ To investigate the causal mechanism by which SQLE affects CD8^+^ T cells, we conducted a conditional screen combined with metabolomic analysis. This revealed that SQLE inhibition in HCC cells markedly reduced multiple oxysterols, with 27HC showing the most pronounced decrease. Notably, previous studies indicate other oxysterols, such as 25HC and 7αHC may exert diverse immunomodulatory effects on T cells. For example, IL-27-induced 25HC selectively inhibits the proliferation of activated T cells in experimental autoimmune dermatitis ^40^ ; whereas in certain tumor contexts, 25HC helps maintain CD8^+^ T cell functionality by limiting effector trogocytosis and preventing CTL exhaustion.^41^ In addition, 7αHC has been identified as a potent inhibitor of TCR signaling and an enhancer of the long-term efficacy of TCR-T cell therapy.^42^ Together, these findings underscore the complex and highly context-dependent roles of oxysterols in immune regulation.

Mechanistically, SQLE depletion in HCC cells not only leads to intracellular cholesterol deficiency, but also downregulates the expression of CYP27A1, the catalytic enzyme for 27HC, by disrupting the activity of the cholesterol-sensing transcription factor *LXRα*. These findings suggested that 27HC may function as a signaling molecule that alters CD8^+^ T cell cholesterol metabolism and activation in the context of HCC. A similar finding has recently been reported in a mouse model of metabolic dysfunction-associated steatohepatitis-induced MASH-HCC, that SQLE drives CD8^+^ T cells immunosuppression mainly via cholesterol accumulation.^43^ Given the complexity of cholesterol metabolism in T cells, both excessive environmental cholesterol and insufficient endogenous cholesterol during TCR activation can impair their immune functions.^9^ Interestingly, these results consistently suggest that SQLE is a promising target for activating T cell functions across different models.

Terbinafine is a Food and Drug Administration-approved antifungal agent widely used in clinical practice for the treatment of dermatophytic infections, due to its ability to inhibit SQLE and disrupt fungal cell membrane integrity.^44^ Recently, the potential antitumor properties of terbinafine have garnered attention, particularly in the context of SQLE-overexpressing tumors.^45^ While generally well-tolerated, its known side effects, such as gastrointestinal discomfort, skin rash, and rare hepatotoxicity,^46^ underscore the need for further investigation to evaluate its efficacy and safety profile in oncological settings.

In conclusion, we identified the role of SQLE-mediated cholesterol metabolism in immune dysfunction within the TME. SQLE inhibition restored CD8^+^ T cell activation and promoted antitumor efficacy. Our findings suggest that SQLE may serve as a prognostic biomarker for immunotherapy response and a potential therapeutic target for combination therapies in HCC.

## Supplementary material

10.1136/jitc-2025-012345 online supplemental file 1

## Data Availability

Data are available upon reasonable request.
