# Supplementary material for: Targeting SQLE-mediated cholesterol metabolism to enhance CD8+ T cell activation and immunotherapy efficacy in hepatocellular carcinoma
Source: J Immunother Cancer. 2025 Sep 26;13(9):e012345. doi: 10.1136/jitc-2025-012345 (PMC12481379; doi:10.1136/jitc-2025-012345)
Supplement: online supplemental file 1 [file jitc-13-9-s001.pdf]

Supplemental information

Targeting SQLE-Mediated Cholesterol Metabolism to Enhance CD8$^+$ T Cell Activation and Immunotherapy Efficacy in Hepatocellular Carcinoma

Shuang Qiao[1†], Hao Zou[1†], Yulan Weng[2], Yifan Liu[1], Weihao Lia[1], Xingjuan Yu[1], Lian Li[2], Limin Zheng[1,2]*, Jing Xu[1]*

*Corresponding author. † These authors contributed equally to this work.

Supplementary Figure S1. Expression of SQLE in public datasets of HCC patients.

Supplementary Figure S2. Suppression of tumoral SQLE enhances CD8+ T cell effector function.

Supplementary Figure S3. SQLE regulates tumor-associated 27HC and CYP27A1 expression through the modulation of cholesterol metabolism.

Supplementary Figure S4. Cholesterol rewiring in CD8+ T cells co-cultured with SQLE-silenced HCC cells.

Supplementary Figure S5. Terbinafine retard tumor growth in HCC mouse models.

Supplementary Table S1. Clinical characteristics of patients.

Supplementary Table S2. Primer list in this study.

Supplementary Table S3. Reagents and sources.

Supplementary Table S4. List of cholesterol related genes.

Supplementary Table S5. DEG of cholesterol related genes in public datasets.

Supplementary Table S6. Survival analysis in TCGA dataset.

Supplementary Table S7. Univariate and multivariate analyses of factors associated with overall survival in SYSUCC cohort 1.

Supplementary Figure S1

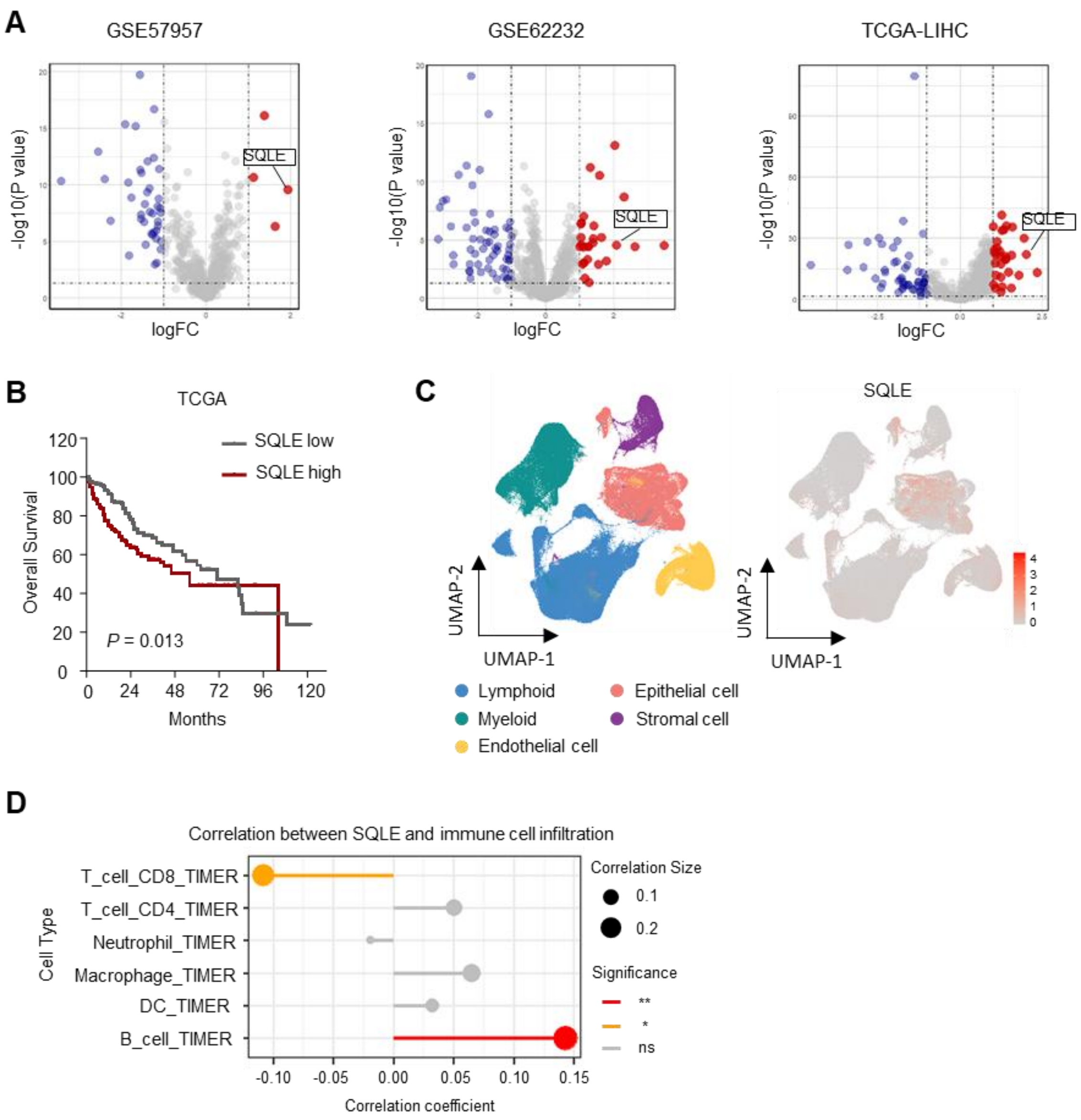

Figure S1. Expression of SQLE in public datasets of HCC patients. (A) Volcano plots showing differential expression genes between non-tumor and tumor regions in HCC patients. (**B)** Kaplan-Meier plot of OS in HCC patients from the TCGA-LICH dataset. Patients were divided into two groups according to the median expression level of SQLE. (**C)** Single cell RNA sequencing analysis of SQLE expression in HCC samples from dataset PRJCA007744. UMAP plot showing clustering of cells into five major subpopulations from human HCC samples. SQLE expression is visualized by density mapping, indicating its predominant expression in the epithelial (tumor) cell compartment. (**D)** Immune cell infiltration levels in HCC tumor samples from the TCGA-LIHC database, analyzed using the “TIMER” algorithm, and then compared between samples with high and low SQLE expression.

Supplementary Figure S2

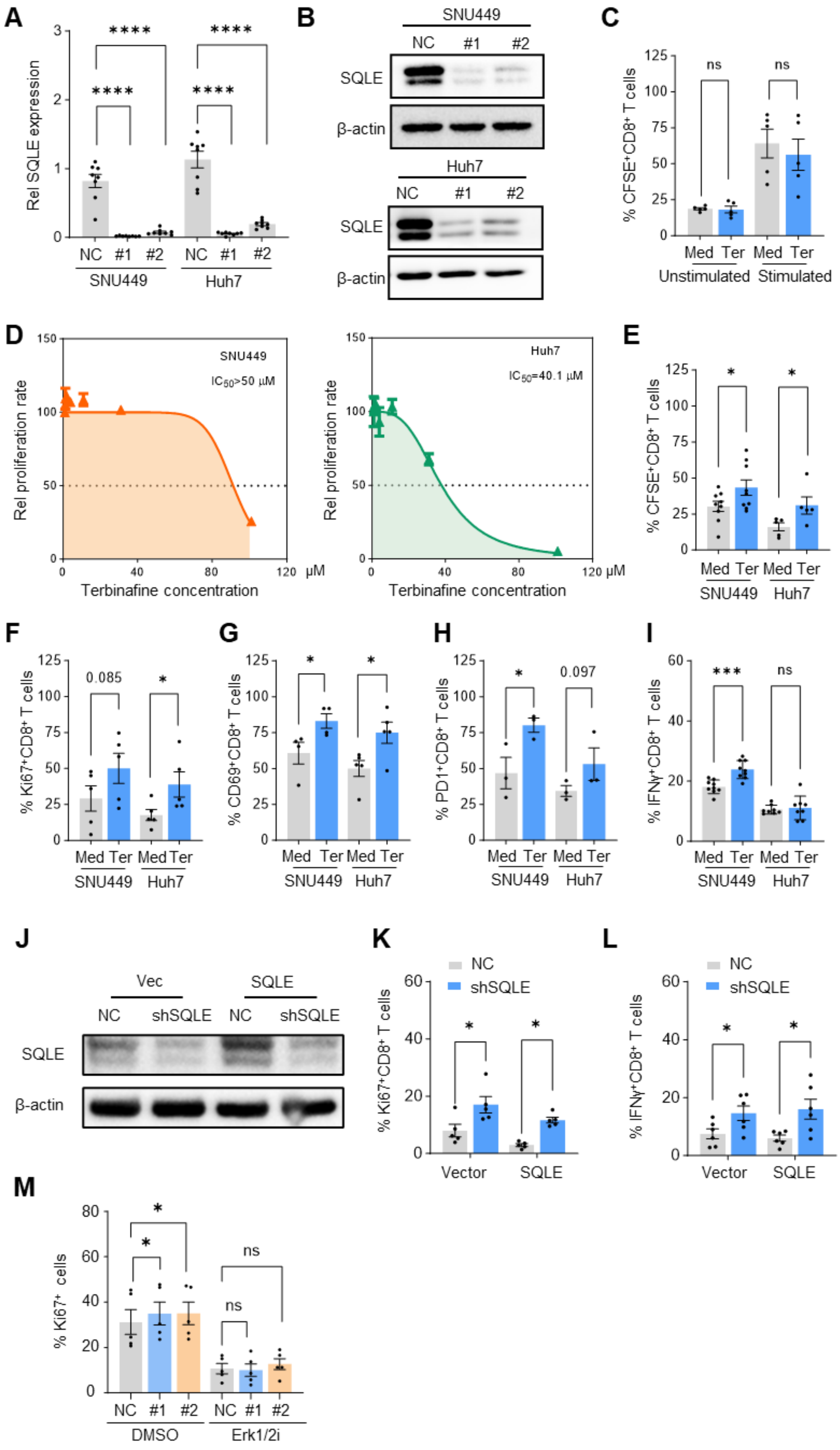

Figure S2. Suppression of tumoral SQLE enhances CD8$^+$ T cell function. (A) Quantitative PCR analysis demonstrated the efficiency of SQLE silencing in HCC cell lines. (**B)** Western blot analysis detected SQLE protein level for confirming silencing efficiency in HCC cell lines. (**C)** Peripheral blood-derived human CD8$^+$ T cells were stimulated with anti-CD3/28 and treated with or without terbinafine. (**D)** IC_{50} curve of terbinafine in the HCC cell lines. **(E)** The proportion of CFSE-labeled human CD8$^+$ T cells were cocultured with HCC cells in the presence or absence of terbinafine for 72 hours (**F)** The proportion of Ki67$^+$CD8$^+$ T cells was analyzed using flow cytometry. (**G)** The proportion of CD69$^+$CD8$^+$ T cells were assessed by flow cytometry. **(H)** The proportions of PD1$^+$CD8$^+$ T cells were determined by flow cytometry. **(I)** The percentages of IFNγ$^+$ CD8$^+$ T cells in the two groups were analyzed by flow cytometry. (**J)** Western blot showing SQLE protein levels in vector or SQLE-overexpressing SNU449 cells subsequently transfected with sh-SQLE or scramble control (NC). **(K)** Flow cytometry analysis of Ki67$^+$ CD8$^+$ T cell proportions in the four indicated groups. **(L)** Flow cytometry analysis of IFNγ$^+$ CD8$^+$ T cell proportions in the four indicated groups. **(M)** The effect of the ERK inhibitor (SCH772984, 1 μM) on CD8$^+$ cells cultured with conditional medium from sh-SQLE or control SNU449 cells.

Supplementary Figure S3

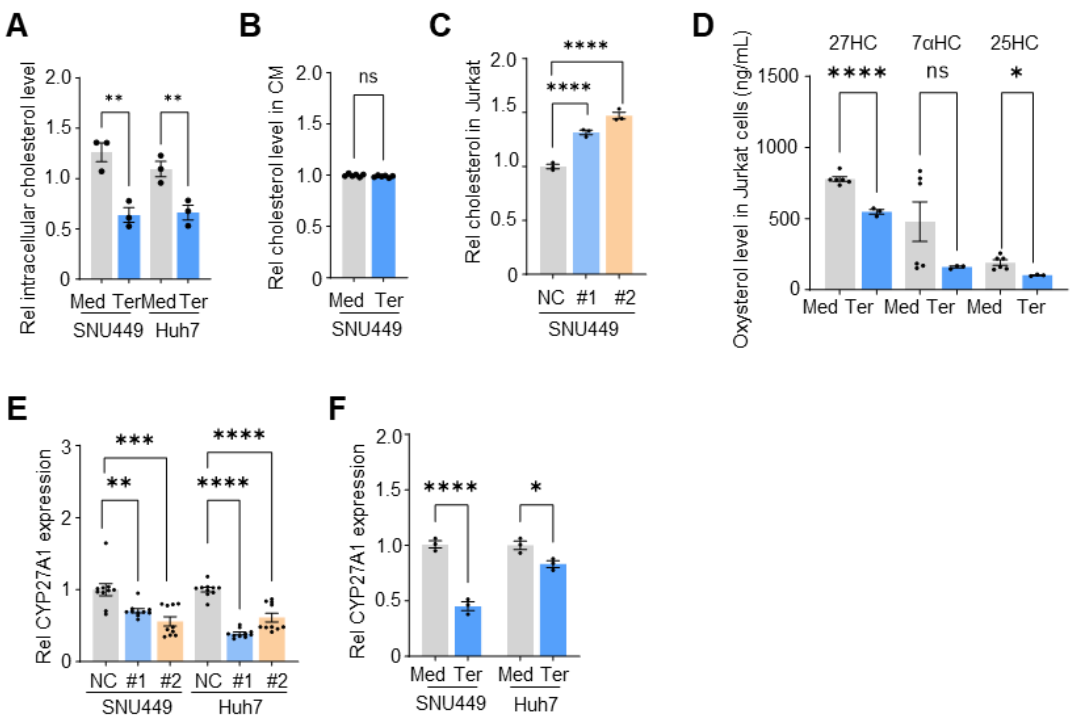

Figure S3. SQLE regulates tumor-associated 27HC and CYP27A1 expression through the modulation of cholesterol metabolism. (A) Intercellular cholesterol level was measured in cell lysates from HCC cells treated with or without terbinafine. (**B)** The cholesterol levels of culture media were determined. (**C)** Cholesterol levels in Jurkat cells were determined in scramble or si-SQLE treated group. **(D)** Jurkat cells were cococultured with SNU449 cells treated with or without terbinafine for 3 days. Levels of indicated oxysterol were quantified by LC/MS. **(E)** Quantitative PCR analysis of CYP27A1 in si-SQLE treated tumor cells, normalized to scramble. **(F)** Quantitative PCR detected the mRNA level of CYP27A1 in terbinafine treated tumor cells, normalized to untreated.

Supplementary Figure S4

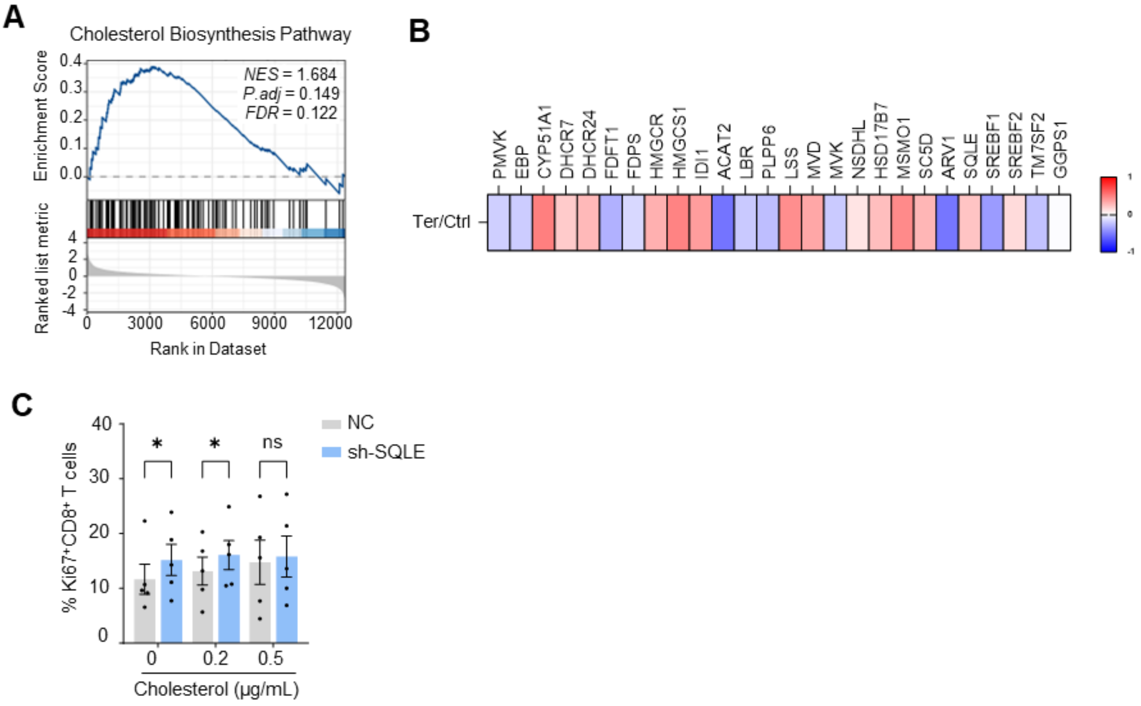

Figure S4. Cholesterol rewiring in CD8$^+$ T cells cocultured with SQLE-silenced HCC cells. (**A**) Enriched cholesterol biosynthesis KEGG pathway from the bulk transcriptomic. **(B)** Transcriptome data of cholesterol metabolism related gene expression in CD8$^+$ T cells co-cultured with si-SQLE tumor cells normalizing to their counterpart with scramble. (**C**) Flow cytometry analysis of KI67$^+$CD8$^+$ T cell proportions cultured with conditioned medium from sh-SQLE or control (NC) SNU449 cells, following increased cholesterol treatment.

Supplementary Figure S5

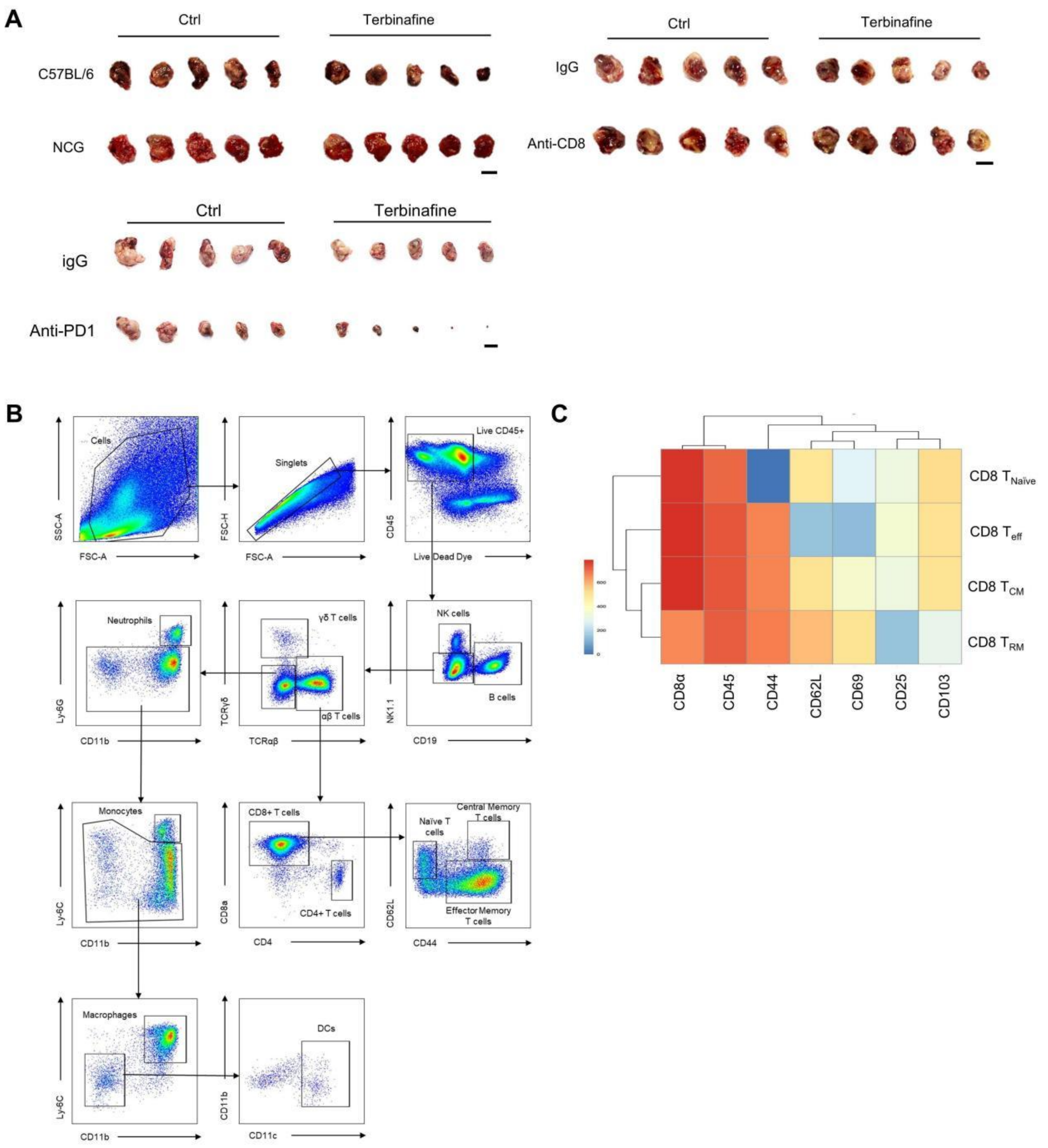

Figure S5. Terbinafine retard tumor growth in HCC mouse models.

(A) Macroscopic images of subcutaneous tumors in Figure 5A and Figure 6A. Scar bar: 1 cm. **(B)** Gating strategy for analyzing immune profile of Hepa1-6 tumors. (**C**) Heatmap of marker genes of $CD8^+$ T cell subpopulations in Figure 5H.

Supplementary Table S1. Clinical characteristics of patients.

Variables	Cohort 1	Cohort 2
Cases (n)	196	23
Age, years (median, range)	50, 13-78	56, 38-70
Gender (female/male)	20/176	1/22
HBsAg (negative/positive)	22/174	1/22
AFP, ng/mL (≤ /> 400)	120/76	8/15
ALT, U/L (≤ /> 40)	109/87	11/12
TBIL, µmol/L (≤ /> 20.5)	127/60*	18/5
Tumor size, cm (≤ /> 5)	69/127	1/22
Tumor number (single/multiple)	154/42	19/4
TNM stage (I-II /III-IV)	139/57	19/4
Differentiation (I-II /III-IV)	129/67	10/13

Abbreviation: AFP, α-fetoprotein; ALT, alanine aminotransferase.

* Value is missing for some patients.

Supplementary Table S2. Primer list.

Gene	Forward Primer (5'-3')	Reverse Primer (5'-3')
HMGCR	TCTTGTGATTGGAGTTGGTA	AGTTACAGGATTCGGCTTAT
HMGCS1	AAGTCACACAAGATGCTACACG	TCAGCGAAGACATCTGGTGCCA
FDFT1	TGTGACCTCTGAACAGGAGTGG	GCCCATAGAGTTGGCACGTTCT
SQLE	TGACAATTCTCATCTGAGGTCCA	CAGGGATACCCTTTAGCAGTTTT
MSMO1	GCTGCCTTTGATTTGTGGAACCT	CTGCACAACCAAAGCATCTTGC
DHCR7	TCCACAGCCATGTGACCAATGC	CGAAGTGGTCATGGCAGATGTC
DHCR24	GCCGCTCTCGCTTATCTTCG	GTCTTGCTACCCTGCTCCTT
LDLR	ACGGCGTCTCTTCCTATGACA	CCCTTGGTATCCGCAACAGA
CD36	CTTTGGCTTAATGAGACTGGGAC	GCAACAAACATCACCACACCA
ABCA1	ACCCACCCTATGAACAACATA	GAGTCGGGTAACGGAAACAGG
ABCG1	ATTCAGGGACCTTTCCTATTCGG	CTCACCACTATTGAACTTCCCG
ACAT1	CCAGCCACTAAGCTTGGTTCCA	GTAGGAGCTTGTCCTTCACCTC
ACAT2	TGGTGCCTTAGCTGCTGTTCCT	GTAGGAGCTTGTCCTTCACCTC
CYP7A1	CAAGCAAACACCATTCCAGCGC	ATAGGATTGCCTTCCAAGCTGAC
CYP7B1	CACCAGAGAACAATTGGACAGC	GCTACCAAGTCTCCCTTTCGCA
CYP39A	GAGGATGACCTGGAGAATCTCC	GCAGCCAAAATGGAGACAACAC
CYP27A1	GTGCTGCCTTTCTGGAAGCGAT	TAGCCAGACACCTGGATGCCAT
CYP46A1	ACGAGACCTCTGCCAACCACTT	AGGTCCTCGAAATCCAGGTACC
CH25H	CAGACCCTCTACCAGCATGTGA	AGGCAGAACAGGATGTGGTGCA
HSD3B1	GTCTTCGGTGTCACTCACAGAG	CTGGTGTAGATGAAGACTGGCC
LSS	GACGACCGATTCACCAAGAGCA	AGACATGCTCCTGGAAGGCAGT
FDPS	CTTTCTTCCTGGTGGCAGATGAC	AGAGCTTCAGCAGGCGGTAGAT
MVD	AAGCGCGATGAAGAGCTGGTTC	TCCTCGGTGAAGTCCTTGCTGA
MVK	GGAAAGTGGACCTCAGCTTACC	GCTTCTCCACTTGCTCTGAGGT
PMVK	GCCTTTCGGAAGGACATGATCC	ACTCTCCGTGTGTCACTCACCA
GGPS1	TGAGAACCAAACTTTCACAGGCAT	CGATGAGTAAACTGGCATTATGCA
CYP51A1	CTCTTACCAGGTTGGCTGCCTT	CTTGAGACTGTCTGCGTTTCTGG
TM7SF2	GGTCAATGGCTTCCAGTTGCTC	AACGCCAGCATGAAGCCAAACC
NSDHL	CAGTTTTCCACTGTGCGTCACC	ACGCCCTCAAAGATGACACTGG
HSD17B7	GCTGATGGACTTCAGGAGGTGT	GCACTGCGAGATGATGTCCAGA
EBP	GCATGGAAACCATCACAGCTTGC	CTGTCAGGAAGTAGAGCACATCC
NR1H2	CTTCGCTAAGCAAGTGCCTGGT	CACTCTGTCTCGTGGTTGTAGC
MYLIP	ACGGTCACCAAGGAATCTGGGA	CCTTCAAGTCACGGCTATACTGC
IDI1	GCCGCAGACTGTGCTCAAAGC	CCTGTTGCTTGTCGAGGTGGTT
LBR	CTATGTGGTGGATGCTCTCTGG	CCACACCAAGTCTCCAAAAGCC
PLPP6	GGTGCTGATGAACCTGCTCTTC	ACTTGTCCACCGAGAGAGTGAC
SC5D	TCTTCTGTGCAACACTGAGCTATT	CCATGGCAATGCCTGGACAGTA
ARV1	CTACACATCTGTGTGCCTCAAAC	CCAGCAGTAAGCCACTCAACAC
SREBF1	ACTTCTGGAGGCATCGCAAGCA	AGGTTCCAGAGGAGGCTACAAG
SREBF2	CTCCATTGACTCTGAGCCAGGA	GAATCCGTGAGCGGTCTACCAT

Supplementary Table S3. Reagent and sources

REAGENT or RESOURCE	SOURCE	IDENTIFIER
Antibodies		
BV605 anti-mouse CD11c	Biolegend	Cat# 117334
BV711 anti-mouse CD8a	Biolegend	Cat# 100759
BV785 anti-mouse CD103	Biolegend	Cat# 121439
FITC anti-mouse CD11b	Biolegend	Cat# 101206
PerCP-Cy5.5 anti-mouse CD45.2	Biolegend	Cat# 103132
PE/Dazzle 594 anti-mouse CD69	Biolegend	Cat# 104536
PE-Cy7 anti-mouse TCRγδ	Biolegend	Cat# 118124
AlexaFluor 647 anti-mouse/human CD44	Biolegend	Cat# 103018
APC/Fire 750 anti-mouse Ly-6G	Biolegend	Cat# 127652
AlexaFluor 700 anti-mouse I-A/I-E	Biolegend	Cat# 107622
PE anti-mouse F4/80	Biolegend	Cat# 111603
BUV395 anti-mouse NK1.1	BD Bioscience	Cat# 564144
BUV563 anti-mouse CD4	BD Bioscience	Cat# 741255
BUV661 anti-mouse CD19	BD Bioscience	Cat# 612971
BUV737 anti-mouse CD62L	BD Bioscience	Cat# 612833
BUV805 anti-mouse TCRb	BD Bioscience	Cat# 748405
BV480 anti-mouse CD25	BD Bioscience	Cat# 566120
APC anti-mouse Ly-6C	BD Bioscience	Cat# 560595
BV421 anti-mouse IFN-γ	BioLegend	Cat# 505829
Brilliant Violet 510 anti-mouse CD279 (PD-1)	Biolegend	Cat# 135241
APC anti-human CD69	Biolegend	Cat# 310910
PE anti-human Ki-67	BD Bioscience	Cat# 556027
PerCP/Cyanine5.5 anti-human CD279 (PD-1)	Biolegend	Cat# 367409
BV421 anti-human IFN-γ	Biolegend	Cat# 502532
LXRα (anti-human/mouse, mouse)	Abcam	Cat# ab41902
SREBP2(anti-human/mouse, rabbit)	Novus	Cat# NB100-74543
SQLE (anti-human/mouse, rabbit mAb)	Proteintech	Cat# 67206-1-Ig
p-Erk1/2 (Thr202/Tyr204) (anti-human/mouse, rabbit mAb)	Cell Signaling Technology	Cat# 4370
Erk1/2 (anti-human/mouse, rabbit mAb)	Cell Signaling Technology	Cat# 4695
β-Actin (anti-human/mouse, rabbit mAb)	Cell Signaling Technology	Cat# 4970
HSP70 (anti-human/mouse, rabbit mAb)	Cell Signaling Technology	Cat# 4872
Goat anti-mouse IgG (H+L) (AlexaFluor488)	Thermo Fisher	Cat# A-28175
Goat anti-rabbit IgG (H+L) (AlexaFluor555)	Thermo Fisher	Cat# A-21429
Biological samples		
Human blood	Guangzhou Blood Center	N/A
Paraffin liver cancer sections	Sun Yat-sen University Cancer Center	Table S1
Chemicals, peptides, and recombinantproteins		
Human IL-2	Peprotech	Cat# 200-02-50UG
Collagenase D	Roche	Cat# 11088858001
DNase I	Roche	Cat# 10104159001
TRIzol	Invitrogen	Cat# 15596018

BrefeldinA	Biolegend	Cat# 420601
Protease Inhibitor Cocktail	Roche	Cat# 11697498001
Phosphatase Inhibitor Cocktail	Roche	Cat# 4906845001
RIPA buffer	Beyotime	Cat# P0013B
RBC Lysis Buffer 10X	Biolegend	Cat# 420301
Lipofectamine™ RNAiMAX Transfection Reagent	Invitrogen	Cat# 13778150
Cell Stimulation Cocktail	Thermo Fisher Scientific	Cat# 00-4970-93
Percoll	Cytiva	Cat# 17089109
Lymphoprep density gradient medium	Stemcell Technologies	Cat# 07851
Critical commercial assays		
Filipin III	Sigma-Aldrich	Cat# SAE0087
Fluo-4 AM	Beyotime	Cat# S1060
CellTrace™ CFSE Cell Proliferation Kit	Invitrogen	Cat# 4C34554
Foxp3/Transcription Factor Fixation/ Permeabilization Kit	Invitrogen	Cat# 00-5521-00
LIVE/DEAD™ Fixable Blue Dead Cell Stain Kit, for UV excitation	Invitrogen	Cat# L23105
Zombie NIR™ Fixable Viability KiT	Biolegend	Cat# 423105
Color Reverse Transcription Kit	EZBioscience	Cat# A0010CGQ
2×SYBR Green qPCR Master Mix	EZBioscience	Cat# A0012-R2-L
EasySep Human CD8+ T Cell Isolation Kit	Stemcell Technologies	Cat# 17953
Ultra-LEAF™ Purified anti-mouse CD3ε Antibody	Biolegend	Cat# 100340
Ultra-LEAF™ Purified anti-mouse CD28 Antibody	Biolegend	Cat# 102116
Total Cholesterol Assay Kit	Jiancheng Bioengineering Institute	Cat# A111-1-1
Experimental models: Cell lines		
Human: SNU-449	ATCC	Cat# CRL-2234
Human: Jurkat	ATCC	Cat# TIB-152
Human: Huh7	Our lab	N/A
Mouse: Hepa 1-6	ATCC	Cat# CRL-1830
Mouse: Hepa 1-6 OVA	Our lab	N/A
Experimental models: Organisms/strains		
Mouse: WT C57BL/6J mouse	Charles River Laboratories (USA)	N/A
Mouse: NOD.Cg-*Prkdc*^{scid} *Il2rg*^{em1Smoc} (NSG)	Sun Yat-sen University Cancer Center	N/A
Oligonucleotides		
qPCR primers	This study	Table S2
Human SQLE siRNA-1: GCACCACAGTTTAAAGCAAAT	GenePharma	N/A
Human SQLE siRNA-2: GCTCAGGCTCTTTATGAATTA	GenePharma	N/A
Recombinant DNA		
pGL3-CYP27A1 promoter	This study	N/A
Software and algorithms		
GraphPad Prism v10.1	GraphPad Software	www.graphpad.com
SPSS Statistics v26.0	IBM	https://www.spss.cm.cn
R v4.0.0	R Project	https://www.r-project.org
SnapGene v6.0.1	Dotmatics	https://www.snapgene.com
FlowJo	FlowJo Software	www.flowjo.com
Image Lab Software v6.0.1	BioRad	https://www.bio-rad.com/

Supplementary Table S4. List of cholesterol related genes

set_name	url	genes
HALLMARK_CHOLESTEROL_HOMEOSTASIS	https://www.gsea-msigdb.org/gsea/msigdb/human/geneset/HALLMARK_CHOLESTEROL_HOMEOSTASIS	ABCA2;ACAT2;ACSS2;ACTG1;ADH4;ALCAM;ALDOC;ANTXR2;ANXA13;ANXA5;ATF3;ATF5;ATXN2;AVPR1A;CBS;CD9;CHKA;CLU;CPEB2;CTNNB1;CXCL16;CYP51A1;DHCR7;EBP;ECH1;ERRFI1;ETHE1;FABP5;FADS2;FASN;FBXO6;FDFT1;FDPS;GLDC;GNAI1;GPX8;GSTM2;GUSB;HMGCR;HMGCS1;HSD17B7;IDI1;JAG1;LDLR;LGALS3;LGMN;LPL;LSS;MAL2;MVD;MVK;NFIL3;NIBAN1;NSDHL;PCYT2;PDK3;PLAUR;PLSCR1;PMVK;PNRC1;PPARG;S100A11;SC5D;SCD;SEMA3B;SQLE;SREBF2;STARD4;STX5;TM7SF2;TMEM97;TNFRSF12A;TP53INP1;TRIB3
GOBP_CELLULAR_RESPONSE_TO_CHOLESTEROL	https://www.gsea-msigdb.org/gsea/msigdb/human/geneset/GOBP_CELLULAR_RESPONSE_TO_CHOLESTEROL	ABCA1;CES1;CYP7A1;DAG1;GPLD1;GPR155;GRAMD1A;GRAMD1B;GRAMD1C;INHBA;INHBB;LRP6;LRP8;MIR182;MIR185;MIR96;MLC1;OSBPL7;PTCH1;SMO
GOBP_CELLULAR_RESPONSE_TO_GLUCOCORTICOID_STIMULUS	https://www.gsea-msigdb.org/gsea/msigdb/human/geneset/GOBP_CELLULAR_RESPONSE_TO_GLUCOCORTICOID_STIMULUS	ANXA1;AQP1;ASS1;ATP5F1A;AXIN2;BCL2L11;CASP9;CFLAR;CRH;CYP1B1;DDIT4;EDN1;EGFR;EIF4E;FAM107A;FBXO32;FECH;FLT3;FOXO3;GJB2;GSK3A;HMGCS2;HNRNPU;IGF1R;ISL1;JAK2;KLF9;METTL21C;MSTN;MT-ND3;MYOD1;NPAS4;NR3C1;PCK1;PCK2;REST;RPS6KB1;SERPINF1;SMYD3;SRD5A1;SSTR2;SSTR3;SSTR4;SSTR5;STC1;TBX2;TFAP4;TGFB1;UBE2L3;UGT1A1;USP8;ZFP36;ZFP36L1;ZFP36L2;ZNF764
GOBP_CELLULAR_RESPONSE_TO_MINERALOCORTICOID_STIMULUS	https://www.gsea-msigdb.org/gsea/msigdb/human/geneset/GOBP_CELLULAR_RESPONSE_TO_MINERALOCORTICOID_STIMULUS	ACE;AIFM1;EDN1;FOXO3;GPER1;IGF1R;NPAS4;RAN;SCNN1A;SCNN1B;SCNN1D;SCNN1G;SGK1
GOBP_CHOLESTEROL_IMPORT	https://www.gsea-msigdb.org/gsea/msigdb/human/geneset/GOBP_CHOLESTEROL_IMPORT	APOA1;APOA2;APOC3;CD36;LAMTOR1;LDLR;SCARB1;STARD4;STARD5
GOBP_CORTISOL_BIOSYNTHETIC_PROCESS	https://www.gsea-msigdb.org/gsea/msigdb/human/geneset/GOBP_CORTISOL_BIOSYNTHETIC_PROCESS	BMP2;BMP5;CACNA1H;CYP11B1;CYP11B2;DGKQ;DKK3;H6PD;REST;WNT4
GOBP_CORTISOL_METABOLIC_PROCESS	https://www.gsea-msigdb.org/gsea/msigdb/human/geneset/GOBP_CORTISOL_METABOLIC_PROCESS	BMP2;BMP5;CACNA1H;CYP11A1;CYP11B1;CYP11B2;DGKQ;DKK3;H6PD;HSD11B2;REST;WNT4
GOBP_HIGH_DENSITY_LIPOPROTEIN_PARTICLE_REMODELING	https://www.gsea-msigdb.org/gsea/msigdb/human/geneset/GOBP_HIGH_DENSITY_LIPOPROTEIN_PARTICLE_REMODELING	ABCA5;ABCG1;APOA1;APOA2;APOA4;APOC1;APOC3;APOE;APOM;CETP;LCAT;LIPC;LIPG;LPL;PLA2G3;PLTP;SCARB1

GOBP_INTESTINAL_CHOLESTEROL_ABSORPTION	https://www.gsea-msigdb.org/gsea/msigdb/human/geneset/GOBP_INTESTINAL_CHOLESTEROL_ABSORPTION	ABCG5;ABCG8;AKR1C1;APOA1;APOA2;APOA4;CD36;CEL;CYP8B1;ENPP7;LDLR;LEP;LIMA1;LPCAT3;NPC1;NPC1L1;PNLIP;SOAT2
GOBP_LOW_DENSITY_LIPOPROTEIN_PARTICLE_REMODELING	https://www.gsea-msigdb.org/gsea/msigdb/human/geneset/GOBP_LOW_DENSITY_LIPOPROTEIN_PARTICLE_REMODELING	ABCG1;AGT;AGTR1;APOA2;APOB;APOE;CETP;LIPC;MPO;MTTP;PLA2G10;PLA2G2A;PLA2G2E;PLA2G3;PLA2G5;PLA2G7
GOBP_NEGATIVE_REGULATION_OF_CHOLESTEROL_EFFLUX	https://www.gsea-msigdb.org/gsea/msigdb/human/geneset/GOBP_NEGATIVE_REGULATION_OF_CHOLESTEROL_EFFLUX	ABCA2;EGF;MIR128-1;MIR130B;MIR144;MIR145;MIR148A;MIR19B1;MIR206;MIR26A1;MIR27A;MIR27B;MIR301B;MIR302A;MIR33A;MIR33B;MIR613;MIR758;MIR9-1;PLA2G10;SHH;SREBF2
GOBP_NEGATIVE_REGULATION_OF_CHOLESTEROL_METABOLIC_PROCESS	https://www.gsea-msigdb.org/gsea/msigdb/human/geneset/GOBP_NEGATIVE_REGULATION_OF_CHOLESTEROL_METABOLIC_PROCESS	APOE;CH25H;ERLIN1;ERLIN2;MIR185;MIR30C1;MIR342;MIR548P;MIR98;SCAP;SOD1
GOBP_NEGATIVE_REGULATION_OF_CHOLESTEROL_STORAGE	https://www.gsea-msigdb.org/gsea/msigdb/human/geneset/GOBP_NEGATIVE_REGULATION_OF_CHOLESTEROL_STORAGE	ABCA1;ABCG1;CES1;MIR146A;NR1H2;NR1H3;PPARA;PPARD;PPARG;TREM2;TTC39B
GOBP_POSITIVE_REGULATION_OF_CHOLESTEROL_EFFLUX	https://www.gsea-msigdb.org/gsea/msigdb/human/geneset/GOBP_POSITIVE_REGULATION_OF_CHOLESTEROL_EFFLUX	ABCA1;ABCA12;ABCA3;ABCA7;ABCA8;ABCG1;ABCG4;ADIPOQ;APOA1;APOE;CAV1;CES1;EEPD1;GPS2;LRP1;MIR206;NFKBIA;NR1H2;NR1H3;PLTP;PON1;PPARG;PTCH1;RXRA;SIRT1;TREM2;ZDHHC8
GOBP_POSITIVE_REGULATION_OF_CHOLESTEROL_METABOLIC_PROCESS	https://www.gsea-msigdb.org/gsea/msigdb/human/geneset/GOBP_POSITIVE_REGULATION_OF_CHOLESTEROL_METABOLIC_PROCESS	ABCG1;ABCG4;APOA1;APOE;CES1;CYP7A1;FGF1;LDLRAP1;MIR182;MIR96;PRKAA1;SCAP;SREBF1;SREBF2;STARD4
GOBP_POSITIVE_REGULATION_OF_CHOLESTEROL_STORAGE	https://www.gsea-msigdb.org/gsea/msigdb/human/geneset/GOBP_POSITIVE_REGULATION_OF_CHOLESTEROL_STORAGE	APOB;CD36;EHD1;LPL;MIR144;MSR1;SCARB1;SREBF2
GOBP_RECEPTOR_MEDIATED_ENDOCYTOSIS_INVOLVED_IN_CHOLESTEROL_TRANSPORT	https://www.gsea-msigdb.org/gsea/msigdb/human/geneset/GOBP_RECEPTOR_MEDIATED_ENDOCYTOSIS_INVOLVED_IN_CHOLESTEROL_TRANSPORT	ABCA2;ANXA2;ANXA2P2;LDLR;LDLRAP1;MIR17;MIR185;MIR27B;PCSK9

GOBP_REGULATION_OF_CHOLESTEROL_EFFLUX	https://www.gsea-msigdb.org/gsea/msigdb/human/geneset/GOBP_REGULATION_OF_CHOLESTEROL_EFFLUX	ABCA1;ABCA12;ABCA2;ABCA3;ABCA5;ABCA7;ABCA8;ABCG1;ABCG4;ADIPOQ;APOA1;APOE;CAV1;CES1;CETP;EEPD1;EGF;GPS2;LAMTOR1;LRP1;MIR128-1;MIR130B;MIR144;MIR145;MIR148A;MIR19B1;MIR206;MIR26A1;MIR27A;MIR27B;MIR301B;MIR302A;MIR33A;MIR33B;MIR613;MIR758;MIR9-1;NAXE;NFKBIA;NR1H2;NR1H3;PLA2G10;PLTP;PON1;PPARG;PTCH1;RXRA;SHH;SIRT1;SREBF2;TREM2;TTC39B;YJEFN3;ZDHHC8
GOBP_REGULATION_OF_CHOLESTEROL_METABOLIC_PROCESS	https://www.gsea-msigdb.org/gsea/msigdb/human/geneset/GOBP_REGULATION_OF_CHOLESTEROL_METABOLIC_PROCESS	ABCA2;ABCG1;ABCG4;ACADL;ACADVL;APOA1;APOB;APOE;AQP8;ARV1;CES1;CH25H;CYP7A1;DGKQ;EPHX2;ERLIN1;ERLIN2;FGF1;FMO5;GNB3;LDLR;LDLRAP1;LPCAT3;MBTPS1;MBTPS2;MIR182;MIR185;MIR30C1;MIR342;MIR548P;MIR96;MIR98;NR1H4;PRKAA1;SCAP;SEC14L2;SERPINA12;SOD1;SREBF1;SREBF2;STARD4;TTC39B
GOBP_REGULATION_OF_CHOLESTEROL_STORAGE	https://www.gsea-msigdb.org/gsea/msigdb/human/geneset/GOBP_REGULATION_OF_CHOLESTEROL_STORAGE	ABCA1;ABCG1;APOB;CD36;CES1;EHD1;LPL;MIR144;MIR146A;MSR1;NR1H2;NR1H3;PPARA;PPARD;PPARG;SCARB1;SREBF2;TREM2;TTC39B
GOBP_TRIGLYCERIDE_RICH_LIPOPROTEIN_PARTICLE_REMODELING	https://www.gsea-msigdb.org/gsea/msigdb/human/geneset/GOBP_TRIGLYCERIDE_RICH_LIPOPROTEIN_PARTICLE_REMODELING	APOA1;APOA2;APOA4;APOA5;APOC2;APOC3;APOE;CETP;LCAT;LIPC;LPL;NR1H4
GOCC_HIGH_DENSITY_LIPOPROTEIN_PARTICLE	https://www.gsea-msigdb.org/gsea/msigdb/human/geneset/GOCC_HIGH_DENSITY_LIPOPROTEIN_PARTICLE	APOA1;APOA2;APOA4;APOA5;APOC1;APOC2;APOC3;APOC4;APOE;APOF;APOH;APOL1;APOM;APOO;CETP;CLU;HDLBP;HPR;LCAT;LIPC;PLA2G7;PLTP;PON1;SAA1;SAA2;SAA4
GOCC_LOW_DENSITY_LIPOPROTEIN_PARTICLE	https://www.gsea-msigdb.org/gsea/msigdb/human/geneset/GOCC_LOW_DENSITY_LIPOPROTEIN_PARTICLE	APOA1;APOA4;APOA5;APOB;APOBR;APOC2;APOE;APOF;APOM;APOO;LDLR;LSR;MSR1;PLA2G7;SELENOS
GOCC_SPHERICAL_HIGH_DENSITY_LIPOPROTEIN_PARTICLE	https://www.gsea-msigdb.org/gsea/msigdb/human/geneset/GOCC_SPHERICAL_HIGH_DENSITY_LIPOPROTEIN_PARTICLE	APOA1;APOA2;APOC2;APOC3;APOM;CLU;HPR;PON1
GOCC_TRIGLYCERIDE_RICH_PLASMA_LIPOPROTEIN_PARTICLE	https://www.gsea-msigdb.org/gsea/msigdb/human/geneset/GOCC_TRIGLYCERIDE_RICH_PLASMA_LIPOPROTEIN_PARTICLE	APOA1;APOA2;APOA4;APOA5;APOB;APOBR;APOC1;APOC2;APOC3;APOC4;APOE;APOH;APOL1;APOM;APOO;LPL;LSR;PCYOX1;SELENOS;VLDLR
GOERING_BLOOD_HDL_CHOLESTEROL_QTL_CIS	https://www.gsea-msigdb.org/gsea/msigdb/human/geneset/GOERING_BLOOD_HDL_CHOLESTEROL_QTL_CIS	GSTM1;HLA-DRB3;HLA-DRB5;IKZF1;LGALS2;LINC00339;PPA2;RPL14;RPS26;TIMM10;TMEM176B;UBA52;UTS2

GOERING_BLOOD_HDL_CHOLESTEROL_QTL_TRANS	https://www.gsea-msigdb.org/gsea/msigdb/human/geneset/GOERING_BLOOD_HDL_CHOLESTEROL_QTL_TRANS	CYP4F35P;EPB41L4A;FLT4;KLK3;LDLRAD4;MAPK8IP1;MPO;MSI1;NACC2;PAF1;SCRIB;SELENON;TMEM241;ZC3HC1
GOMF_CHOLESTEROL_BINDING	https://www.gsea-msigdb.org/gsea/msigdb/human/geneset/GOMF_CHOLESTEROL_BINDING	ABCA1;ABCG1;ANXA6;APOA1;APOA2;APOC3;APOD;APOF;CAV1;CD81;CETP;ERLIN1;ERLIN2;GPR155;GRAMD1A;GRAMD1B;GRAMD1C;MINAR2;NPC1;NPC1L1;NPC2;NR1H3;OSBP2;OSBPL10;OSBPL1A;OSBPL2;OSBPL3;OSBPL5;OSBPL6;OSBPL7;OSBPL8;PMP2;PROM1;PROM2;PTCH1;SCARB2;SCP2;SIDT1;SLC38A9;SOAT1;SOAT2;STAR;STARD3;STARD3NL;STARD4;STARD5;SULT2B1;SYP;TMEM97;TSPO;TSPO2;VDAC1;VDAC2
GOMF_LOW_DENSITY_LIPOPROTEIN_PARTICLE_BINDING	https://www.gsea-msigdb.org/gsea/msigdb/human/geneset/GOMF_LOW_DENSITY_LIPOPROTEIN_PARTICLE_BINDING	CD36;CDH13;COLEC12;CRP;LDLR;LIPC;MSR1;PCSK9;PLTP;SAMD1;SCARB1;SCARF1;SORL1;STAB1;STAB2;THBS1;TREM2
HP_ABNORMAL_CIRCULATING_CHOLESTEROL_CONCENTRATION	https://www.gsea-msigdb.org/gsea/msigdb/human/geneset/HP_ABNORMAL_CIRCULATING_CHOLESTEROL_CONCENTRATION	ABCA1;ABCA2;ABCB4;ABCG5;ABCG8;AGPAT2;ALB;ALG12;ALG6;ALG9;ALMS1;ANGPTL3;APOA1;APOA2;APOA5;APOB;APOC2;APOC3;APOE;APTX;ARL6;ATAD3A;ATP6AP1;B4GALT1;BBIP1;BBS1;BBS10;BBS12;BBS2;BBS4;BBS5;BBS7;BBS9;BSCL2;CAV1;CAV3;CAVIN1;CCDC115;CELA2A;CEP19;CEP290;CETP;CFAP418;COG4;CREB3L3;CYP11A1;CYP27A1;CYP7A1;CYP7B1;DEAF1;DGAT1;DHCR24;DHCR7;DIO1;DLK1;DYRK1B;EBP;EMD;EPHX2;FDFT1;FHL1;FLCN;FLII;FOS;GALK1;GALNT2;GBA1;GHR;GPIHBP1;HERC2;HSD3B7;HTT;IFT172;IFT27;IFT56;IFT74;IL12A;IL12RB1;IQSEC2;IRF5;JAG1;KIF12;LCAT;LDLR;LDLRAP1;LIPA;LIPC;LMNA;LPL;LRP6;LZTFL1;MAGEL2;MEF2A;MEG3;MKKS;MKRN3;MKS1;MMEL1;MSMO1;MTTP;MYO5B;NGLY1;NPAP1;NPHP1;NSDHL;NUP107;OCRL;PCSK9;PEX12;PHKA2;PHKB;PHKG2;PIK3R5;PMM2;PNKP;PNLIP;POU2AF1;PPARG;PPP1R17;PSAP;PSMB8;PWAR1;PWRN1;PYGL;RAI1;RSPO1;RTL1;SAR1B;SC5D;SCAPER;SCARB2;SCLT1;SDCCAG8;SETX;SLC25A13;SLC25A36;SLC2A3;SLC37A4;SLC7A7;SMPD1;SNORD115-1;SNORD116-1;SPIB;STX5;SYNE1;SYNE2;TBL1X;TDP1;TMEM199;TMEM43;TNFSF15;TNPO3;TRIM32;TSHB;TTC8;TTPA;UBE3B;UBR1;WDPCP
HP_ABNORMAL_LDL_CHOLESTEROL_CONCENTRATION	https://www.gsea-msigdb.org/gsea/msigdb/human/geneset/HP_ABNORMAL_LDL_CHOLESTEROL_CONCENTRATION	ABCA2;ABCG5;ABCG8;ALB;ALG6;ANGPTL3;APOA2;APOA5;APOB;APOC3;APOE;B4GALT1;CCDC115;CELA2A;CEP19;CYP7A1;DYRK1B;EMD;EPHX2;FDFT1;FHL1;GHR;GPIHBP1;LCAT;LDLR;LDLRAP1;LIPA;LMNA;LPL;LRP6;MSMO1;MTTP;NGLY1;PCSK9;PPP1R17;SAR1B;SLC25A13;SLC7A7;SMPD1;SYNE1;SYNE2;TMEM199;TMEM43;TTPA

HP_ABNORMALITY_OF_LIPOPROTEIN_CHOLESTEROL_CONCENTRATION	https://www.gsea-msigdb.org/gsea/msigdb/human/geneset/HP_ABNORMALITY_OF_LIPOPROTEIN_CHOLESTEROL_CONCENTRATION	ABCA1;ABCA2;ABCG5;ABCG8;ALB;ALG6;ALMS1;ANGPTL3;APOA1;APOA2;APOA5;APOB;APOC2;APOC3;APOE;ARL6;B4GALT1;BBIP1;BBS1;BBS10;BBS12;BBS2;BBS4;BBS5;BBS7;BBS9;CCDC115;CELA2A;CEP19;CEP290;CETP;CFAP418;CREB3L3;CYP7A1;DYRK1B;EMD;EPHX2;FDFT1;FHL1;GALNT2;GBA1;GHR;GPIHBP1;HERC2;IFT172;IFT27;IFT74;LCAT;LDLR;LDLRAP1;LIPA;LIPC;LMNA;LPL;LRP6;LZTFL1;MAGEL2;MKKS;MKRN3;MKS1;MSMO1;MTTP;NGLY1;NPAP1;NPHP1;PCSK9;PPARG;PPP1R17;PSMB8;PWAR1;PWRN1;SAR1B;SCAPER;SCARB2;SCLT1;SDCCAG8;SLC25A13;SLC7A7;SMPD1;SNORD115-1;SNORD116-1;SYNE1;SYNE2;TMEM199;TMEM43;TRIM32;TTC8;TTPA;UBR1;WDPCP
HP_DECREASED_LDL_CHOLESTEROL_CONCENTRATION	https://www.gsea-msigdb.org/gsea/msigdb/human/geneset/HP_DECREASED_LDL_CHOLESTEROL_CONCENTRATION	ALG6;ANGPTL3;APOA5;APOB;APOC3;B4GALT1;FDFT1;GPIHBP1;LDLRAP1;MSMO1;MTTP;NGLY1;SAR1B
HP_HYPERCHOLESTEROLEMIA	https://www.gsea-msigdb.org/gsea/msigdb/human/geneset/HP_HYPERCHOLESTEROLEMIA	ABCB4;ABCG5;ABCG8;AGPAT2;ALB;APOB;APOC2;APOE;APTX;ATP6AP1;BSCL2;CAV1;CAV3;CAVIN1;CCDC115;CEP19;CETP;COG4;CREB3L3;CYP7A1;DEAF1;DGAT1;DIO1;DLK1;DYRK1B;FLII;FOS;GALK1;GHR;HERC2;IFT172;IFT56;IL12A;IL12RB1;IQSEC2;IRF5;JAG1;KIF12;LDLR;LDLRAP1;LIPA;LIPC;LMNA;LPL;MAGEL2;MEF2A;MEG3;MKRN3;MMEL1;MYO5B;NPAP1;NUP107;OCRL;PCSK9;PHKA2;PHKB;PHKG2;PIK3R5;PNKP;POU2AF1;PPARG;PWAR1;PWRN1;PYGL;RAI1;RSPO1;RTL1;SETX;SLC25A13;SLC25A36;SLC37A4;SLC7A7;SNORD115-1;SNORD116-1;SPIB;STX5;TBL1X;TDP1;TMEM199;TNFSF15;TNPO3;TSHB;TTPA
HP_HYPERLIPOPROTEINEMIA	https://www.gsea-msigdb.org/gsea/msigdb/human/geneset/HP_HYPERLIPOPROTEINEMIA	ABCA2;ABCG5;ABCG8;ALB;APOA2;APOA5;APOB;APOC2;APOC3;APOE;CCDC115;CELA2A;CEP19;CETP;CYP7A1;DYRK1B;EMD;EPHX2;FHL1;GHR;GPIHBP1;LCAT;LDLR;LDLRAP1;LIPA;LIPC;LMNA;LPL;LRP6;PCSK9;PPP1R17;SLC25A13;SLC7A7;SMPD1;SYNE1;SYNE2;TMEM199;TMEM43;TTPA;UBR1
HP_HYPOCHOLESTEROLEMIA	https://www.gsea-msigdb.org/gsea/msigdb/human/geneset/HP_HYPOCHOLESTEROLEMIA	ABCA1;ALG12;ALG9;APOB;ATAD3A;DHCR7;FDFT1;FLCN;HSD3B7;MSMO1;MTTP;PEX12;PMM2;PNLIP;PSAP;SAR1B;UBE3B

HP_HYPOLIPOPROTEINEMIA	https://www.gsea-msigdb.org/gsea/msigdb/human/geneset/HP_HYPOLIPOPROTEINEMIA	ABCA1;ALG6;ALMS1;ANGPTL3;APOA1;APOA5;APOB;APOC3;APOE;ARL6;B4GALT1;BBIP1;BBS1;BBS10;BBS12;BBS2;BBS4;BBS5;BBS7;BBS9;CELA2A;CEP19;CEP290;CFAP418;CREB3L3;FDFT1;GALNT2;GBA1;GPIHBP1;HERC2;IFT172;IFT27;IFT74;LCAT;LDLRAP1;LIPA;LMNA;LZTFL1;MAGEL2;MKKS;MKRN3;MKS1;MSMO1;MTTP;NGLY1;NPAP1;NPHP1;PPARG;PSMB8;PWAR1;PWRN1;SAR1B;SCAPER;SCARB2;SCLT1;SDCCAG8;SLC25A13;SLC7A7;SMPD1;SNORD115-1;SNORD116-1;TRIM32;TTC8;WDPCP
HP_INCREASED_LDL_CHOLESTEROL_CONCENTRATION	https://www.gsea-msigdb.org/gsea/msigdb/human/geneset/HP_INCREASED_LDL_CHOLESTEROL_CONCENTRATION	ABCA2;ABCG5;ABCG8;ALB;APOA2;APOB;APOE;CCDC115;CELA2A;CEP19;CYP7A1;DYRK1B;EMD;EPHX2;FHL1;GHR;LCAT;LDLR;LDLRAP1;LIPA;LMNA;LPL;LRP6;PCSK9;PPP1R17;SLC25A13;SLC7A7;SMPD1;SYNE1;SYNE2;TMEM199;TMEM43;TTPA
KEGG_MEDICUS_REFERENCE_BILE_ACID_BIOSYNTHESIS	https://www.gsea-msigdb.org/gsea/msigdb/human/geneset/KEGG_MEDICUS_REFERENCE_BILE_ACID_BIOSYNTHESIS	ACOT8;ACOX2;AKR1C4;AKR1D1;AMACR;CYP27A1;CYP39A1;CYP7A1;CYP7B1;CYP8B1;HSD17B4;HSD3B7;SCP2;SLC27A5
KEGG_MEDICUS_REFERENCE_CHOLESTEROL_BIOSYNTHESIS	https://www.gsea-msigdb.org/gsea/msigdb/human/geneset/KEGG_MEDICUS_REFERENCE_CHOLESTEROL_BIOSYNTHESIS	CYP51A1;DHCR24;DHCR7;EBP;FDFT1;HSD17B7;LBR;LSS;MSMO1;NSDHL;SC5D;SQLE;TM7SF2
KEGG_MEDICUS_REFERENCE_MEVALONATE_PATHWAY	https://www.gsea-msigdb.org/gsea/msigdb/human/geneset/KEGG_MEDICUS_REFERENCE_MEVALONATE_PATHWAY	ACAT1;ACAT2;FDPS;GGPS1;HMGCR;HMGCS1;HMGCS2;IDI1;IDI2;MVD;MVK;PMVK
KEGG_PRIMARY_BILE_ACID_BIOSYNTHESIS	https://www.gsea-msigdb.org/gsea/msigdb/human/geneset/KEGG_PRIMARY_BILE_ACID_BIOSYNTHESIS	ACOX2;AKR1C4;AKR1D1;AMACR;BAAT;CH25H;CYP27A1;CYP39A1;CYP46A1;CYP7A1;CYP7B1;CYP8B1;HSD17B4;HSD3B7;SCP2;SLC27A5
KEGG_STEROID_HORMONE_BIOSYNTHESIS	https://www.gsea-msigdb.org/gsea/msigdb/human/geneset/KEGG_STEROID_HORMONE_BIOSYNTHESIS	AKR1C1;AKR1C2;AKR1C3;AKR1C4;AKR1D1;COMT;CYP11A1;CYP11B1;CYP11B2;CYP17A1;CYP19A1;CYP1A1;CYP1B1;CYP21A2;CYP3A4;CYP3A43;CYP3A5;CYP3A7;CYP7A1;CYP7B1;HSD11B1;HSD11B2;HSD17B1;HSD17B12;HSD17B2;HSD17B3;HSD17B6;HSD17B7;HSD17B8;HSD3B1;HSD3B2;SRD5A1;SRD5A2;SRD5A3;STS;SULT1E1;SULT2B1;UGT1A1;UGT1A10;UGT1A3;UGT1A4;UGT1A5;UGT1A6;UGT1A7;UGT1A8;UGT1A9;UGT2A1;UGT2A3;UGT2B10;UGT2B11;UGT2B15;UGT2B17;UGT2B28;UGT2B4;UGT2B7
LIPID_RAFT	https://www.gsea-msigdb.org/gsea/msigdb/human/geneset/LIPID_RAFT	ABCA1;BTK;CARD11;CAV1;CAV2;CD24;CD48;CD55;CD79A;CLN3;FLOT1;GHSR;ITLN1;LAT;LAT2;LAX1;LCK;LYN;MAL;MALL;PAG1;PI4K2A;PIKFYVE;PPT1;PRNP;SHH;SORBS1;STOM;STX12

REACTOME_CHOLESTEROL_BIOSYNTHESIS	https://www.gsea-msigdb.org/gsea/msigdb/human/geneset/REACTOME_CHOLESTEROL_BIOSYNTHESIS	ACAT2;ARV1;CYP51A1;DHCR24;DHCR7;EBP;FDFT1;FDPS;GGPS1;HMGCR;HMGCS1;HSD17B7;IDI1;IDI2;LBR;LSS;MSMO1;MVD;MVK;NSDHL;PLPP6;PMVK;SC5D;SQLE;SREBF1;SREBF2;TM7SF2
REACTOME_NR1H2_NR1H3_REGULATE_GENE_EXPRESSION_TO_LIMIT_CHOLESTEROL_UPTAKE	https://www.gsea-msigdb.org/gsea/msigdb/human/geneset/REACTOME_NR1H2_NR1H3_REGULATE_GENE_EXPRESSION_TO_LIMIT_CHOLESTEROL_UPTAKE	MYLIP;NR1H2;NR1H3;RXRA;RXRB
REACTOME_NR1H3_NR1H2_REGULATE_GENE_EXPRESSION_LINKED_TO_CHOLESTEROL_TRANSPORT_AND_EFFLUX	https://www.gsea-msigdb.org/gsea/msigdb/human/geneset/REACTOME_NR1H3_NR1H2_REGULATE_GENE_EXPRESSION_LINKED_TO_CHOLESTEROL_TRANSPORT_AND_EFFLUX	ABCA1;ABCG1;ABCG5;ABCG8;AGO1;AGO2;AGO3;AGO4;APOC1;APOC2;APOC4;APOD;APOE;ARL4C;CETP;EEPD1;EP300;GPS2;HDAC3;KDM1A;KDM1B;KDM3A;KDM4A;MOV10;NCOA1;NCOR1;NCOR2;NR1H2;NR1H3;PLTP;RXRA;RXRB;TBL1X;TBL1XR1;TNRC6A;TNRC6B;TNRC6C
REACTOME_REGULATION_OF_CHOLESTEROL_BIOSYNTHESIS_BY_SREBP_SREBF	https://www.gsea-msigdb.org/gsea/msigdb/human/geneset/REACTOME_REGULATION_OF_CHOLESTEROL_BIOSYNTHESIS_BY_SREBP_SREBF	ACACA;ACACB;CARM1;CHD9;CREBBP;CYP51A1;DHCR7;ELOVL6;FASN;FDFT1;FDPS;GGPS1;GPAM;HELZ2;HMGCR;HMGCS1;IDI1;INSIG1;INSIG2;KPNB1;LSS;MBTPS1;MBTPS2;MED1;MTF1;MVD;MVK;NCOA1;NCOA2;NCOA6;NFYA;NFYB;NFYC;PMVK;PPARA;RAN;RXRA;SAR1B;SC5D;SCAP;SCD;SEC23A;SEC24A;SEC24B;SEC24C;SEC24D;SMARCD3;SP1;SQLE;SREBF1;SREBF2;TBL1X;TBL1XR1;TGS1;TM7SF2
WP_CHOLESTEROL_BIOSYNTHESIS_PATHWAY	https://www.gsea-msigdb.org/gsea/msigdb/human/geneset/WP_CHOLESTEROL_BIOSYNTHESIS_PATHWAY	CYP51A1;DHCR7;FDFT1;FDPS;HMGCR;HMGCS1;IDI1;LSS;MSMO1;MVD;MVK;NSDHL;PMVK;SC5D;SQLE
WP_CHOLESTEROL_BIOSYNTHESIS_PATHWAY_IN_HEPATOCYTES	https://www.gsea-msigdb.org/gsea/msigdb/human/geneset/WP_CHOLESTEROL_BIOSYNTHESIS_PATHWAY_IN_HEPATOCYTES	ABCA1;ABCB11;ABCD3;ABCG1;ACAT2;ACBD3;ACOT1;ACOT2;ACOT8;ACSL1;ACSL3;AKR1C4;AKR1D1;AMACR;BAAT;CH25H;CLIP3;CYB5R1;CYB5R2;CYB5R3;CYP11A1;CYP11B1;CYP11B2;CYP17A1;CYP19A1;CYP1A1;CYP21A2;CYP27A1;CYP27B1;CYP2R1;CYP39A1;CYP3A4;CYP3A7;CYP46A1;CYP51A1;CYP7A1;CYP7B1;CYP8B1;DHCR24;DHCR7;DHRS11;EBP;ELOVL2;ELOVL3;ELOVL4;ELOVL5;ERG28;FADS2;FDFT1;FDPS;FDX1;FDXR;GGPS1;HINT2;HMGCR;HMGCS1;HMGCS2;HNF1A;HSD17B1;HSD17B10;HSD17B11;HSD17B12;HSD17B2;HSD17B3;HSD17B7;HSD17B8;HSD3B1;HSD3B2;HSD3B7;IDI1;IDI2;LBR;LSS;MSMO1;MVD;MVK;MYLIP;NR1H2;NR1H3;NSDHL;OSBP;OSBPL1A;OSBPL2;OSBPL3;OSBPL6;OSBPL7;OSBPL9;PBX1;PCSK9;PLPP6;PMVK;PRKAA1;PRKAA2;PRLR;RDH8;SC5D;SCD;SCP2;SCP2D1;SDR42E1;SDR42E2;SERINC1;SLC22A24;SLC27A2;SLC27A5;SRD5A1;SRD5A2;SREBF1;STAR;TM7SF2;TSPO

WP_CHOLESTEROL_BIOSYNTHESIS_WITH_SKELETAL_DYSPLASIAS	https://www.gsea-msigdb.org/gsea/msigdb/human/geneset/WP_CHOLESTEROL_BIOSYNTHESIS_WITH_SKELETAL_DYSPLASIAS	CYP51A1;DHCR24;DHCR7;EBP;LBR;NSDHL;SC5D
WP_CHOLESTEROL_METABOLISM	https://www.gsea-msigdb.org/gsea/msigdb/human/geneset/WP_CHOLESTEROL_METABOLISM	ABCA1;ABCB11;ABCG5;ABCG8;ACAT2;ANGPTL3;ANGPTL8;APOA1;APOA2;APOA4;APOB;APOC1;APOC2;APOC3;APOE;APOH;CD36;CETP;CIDEB;CYP27A1;CYP51A1;CYP7A1;DGAT1;DHCR24;DHCR7;EBP;FABP2;FDFT1;FDPS;HMGCR;HMGCS1;HSD17B7;IDI1;LBR;LCAT;LDLR;LDLRAP1;LIPA;LIPC;LIPG;LPA;LPL;LRP1;LRPAP1;LSS;MGAT1;MSMO1;MTTP;MVD;MVK;MYLIP;NPC1;NPC1L1;NPC2;NSDHL;OSBPL5;PCSK9;PLTP;PMVK;SAR1B;SC5D;SCARB1;SLC27A4;SOAT1;SORT1;SQLE;STAR;STARD3;TM7SF2;TSPO;VAPA;VDAC1
WP_CHOLESTEROL_METABOLISM_WITH_BLOCH_AND_KANDUTSCHRUSSELL_PATHWAYS	https://www.gsea-msigdb.org/gsea/msigdb/human/geneset/WP_CHOLESTEROL_METABOLISM_WITH_BLOCH_AND_KANDUTSCHRUSSELL_PATHWAYS	ABCA1;ABCG1;ACAT2;ACOT2;ACSL1;ACSL3;ACSL4;CH25H;CYP27A1;CYP46A1;CYP51A1;DHCR24;DHCR7;EBP;ELOVL2;ELOVL3;ELOVL4;ELOVL5;FADS1;FADS2;FASN;FDFT1;FDPS;GGPS1;HMGCR;HMGCS1;HMGCS2;HSD17B7;IDI1;LBR;LSS;MSMO1;MVD;MVK;MYLIP;NR1H2;NR1H3;NSDHL;PMVK;SC5D;SCD;SOAT1;SOAT2;SQLE;SREBF1;SREBF2;TM7SF2
WP_CHOLESTEROL_SYNTHESIS_DISORDERS	https://www.gsea-msigdb.org/gsea/msigdb/human/geneset/WP_CHOLESTEROL_SYNTHESIS_DISORDERS	CYP51A1;DHCR24;DHCR7;EBP;FDFT1;FDPS;HMGCR;HMGCS1;HSD3B2;IDI1;LBR;LSS;MSMO1;MVD;MVK;PMVK;SC5D;SQLE
WP_ENTEROCYTE_CHOLESTEROL_METABOLISM	https://www.gsea-msigdb.org/gsea/msigdb/human/geneset/WP_ENTEROCYTE_CHOLESTEROL_METABOLISM	ABCA1;ABCG5;ABCG8;ACAT2;APOA1;APOA4;APOB;CD36;CYP51A1;DGAT1;DHCR24;DHCR7;EBP;FABP2;FDFT1;FDPS;HMGCR;HMGCS1;HSD17B7;IDI1;LBR;LDLR;LSS;MGAT1;MSMO1;MTTP;MVD;MVK;NPC1L1;NSDHL;PMVK;SAR1B;SC5D;SLC27A4;SQLE;TM7SF2
WP_MEVALONATE_ARM_OF_CHOLESTEROL_BIOSYNTHESIS_PATHWAY	https://www.gsea-msigdb.org/gsea/msigdb/human/geneset/WP_MEVALONATE_ARM_OF_CHOLESTEROL_BIOSYNTHESIS_PATHWAY	ACAT1;ACAT2;FDFT1;FDPS;GGPS1;HMGCL;HMGCR;HMGCS1;IDI1;IDI2;MVD;MVK;PMVK
WP_OXYSTEROLS_DERIVED_FROM_CHOLESTEROL	https://www.gsea-msigdb.org/gsea/msigdb/human/geneset/WP_OXYSTEROLS_DERIVED_FROM_CHOLESTEROL	ACOT1;ACOT11;ACOT12;ACOT13;ACOT2;ACOT4;ACOT6;ACOT7;ACOT8;ACOT9;ACOX2;AKR1C4;AKR1D1;AMACR;BAAT;CH25H;CYP27A1;CYP39A1;CYP3A4;CYP46A1;CYP7A1;CYP7B1;CYP8B1;DBP;DHCR7;EBP;EPHX2;ESR1;ESR2;GPR183;HSD11B1;HSD11B2;HSD3B7;IL17A;IL17B;IL17C;IL17D;IL17F;IL25;INSIG1;LBP;NR1H2;NR1I2;RORC;SCP2;SLC27A2;SLC27A5;SULT2A1;SULT2B1;THEM5;UGT3A1

WP_SREBF_AND_MIR33_IN_CHOLESTEROL_AND_LIPID_HOMEOSTASIS	https://www.gsea-msigdb.org/gsea/msigdb/human/geneset/WP_SREBF_AND_MIR33_IN_CHOLESTEROL_AND_LIPID_HOMEOSTASIS	ABCA1;FASN;HMGCR;HMGCS1;LDLR;MED15;MIR33A;MIR33B;MTOR;NR1H3;PPARA;PPARGC1A;PRKAA1;SCD;SIRT1;SIRT6;SREBF1;SREBF2
WP_STATIN_INHIBITION_OF_CHOLESTEROL_PRODUCTION	https://www.gsea-msigdb.org/gsea/msigdb/human/geneset/WP_STATIN_INHIBITION_OF_CHOLESTEROL_PRODUCTION	ABCA1;ABCG5;ABCG8;ACSS1;APOA1;APOA2;APOA4;APOA5;APOB;APOC1;APOC2;APOC3;APOE;CETP;CYP7A1;DGAT1;FDFT1;HMGCR;LCAT;LDLR;LIPC;LPL;LRP1;MIR33A;MIR33B;MTTP;PDIA2;PLTP;SCARB1;SOAT1;SQLE

Supplementary Table S5. DEG of cholesterol related genes in public datasets

	GSE57957					GSE62232					TCGA-LIHC				
**Gene.symbol**	**t**	**B**	**logFC**	**adj.P.Val**	**P.Value**	**t**	**B**	**logFC**	**adj.P.Val**	**P.Value**	**t**	**B**	**logFC**	**adj.P.Val**	**P.Value**
ABCA1	-2.707232	-3.211398	-0.301068	0.0344	0.00832	-2.344344	-3.965681	-0.211529	0.0517	0.0212	-3.501977	-1.385607	-0.380952	0.000995	0.000512
ABCA12	-0.138868	-6.695523	-0.005366	0.933	0.89	-1.496236	-5.516347	-0.081436	0.226	0.138	1.296428	-6.5843	0.041309	0.244961	0.195545
ABCA3	2.372927	-3.993289	0.101661	0.0676	0.0201	-0.954542	-6.164208	-0.105256	0.458	0.342	4.984594	4.630278	0.577471	2.50E-06	9.12E-07
ABCA5	3.734163	-0.308773	0.305701	0.00256	0.000355	1.054667	-6.065428	0.29397	0.409	0.294	4.365591	1.884659	0.388251	3.86E-05	1.60E-05
ABCA7	-1.148769	-6.052282	-0.038037	0.409	0.254	2.74104	-3.031737	0.242219	0.0226	0.00737	7.385916	18.17694	0.607996	4.31E-12	8.32E-13
ABCA8	-7.122952	12.743	-1.388307	3.06E-08	4.43E-10	-2.834345	-2.794054	-1.89699	0.0184	0.00565	-6.988318	15.63848	-0.981377	5.13E-11	1.11E-11
ABCB11	-2.560923	-3.564294	-0.316473	0.0467	0.0124	0.329504	-6.560495	0.167736	0.816	0.743	-4.139925	0.966047	-0.944847	9.42E-05	4.21E-05
ABCB4	-2.473365	-3.767569	-0.507551	0.0557	0.0155	-0.795508	-6.301233	-0.439403	0.544	0.428	-2.945706	-3.132913	-0.637014	0.005813	0.003403
ABCD3	-2.803655	-2.969977	-0.229023	0.0278	0.00636	-2.207026	-4.259055	-0.329007	0.0677	0.0298	-2.828009	-3.465632	-0.255981	0.00825	0.00491
ABCG1	3.072901	-2.259946	0.441951	0.0149	0.00291	1.768493	-5.087801	0.362629	0.148	0.0803	4.11844	0.880926	0.531434	0.000102	4.60E-05
ABCG4	-2.150238	-4.464621	-0.155268	0.101	0.0346	-4.306741	1.755449	-0.316118	0.000445	4.17E-05	0.094347	-7.417587	0.001787	0.948103	0.924879
ABCG5	-2.338164	-4.069532	-0.228152	0.0722	0.0219	-1.109907	-6.006818	-0.514605	0.381	0.27	-1.958363	-5.515364	-0.335926	0.072173	0.050854
ABCG8	-4.082835	0.826322	-0.539598	0.000925	0.000106	-0.411132	-6.530521	-0.134511	0.768	0.682	-2.59248	-4.092161	-0.443335	0.015956	0.009864
ACACA	5.916336	7.69605	0.784061	2.18E-06	8.13E-08	4.563443	2.681958	0.880428	0.000215	1.57E-05	9.78347	35.59622	0.921441	2.05E-19	1.74E-20
ACACB	-7.299042	13.49833	-1.332143	1.63E-08	2.04E-10	-3.54169	-0.785123	-0.400931	0.00339	0.000629	-5.55442	7.441218	-0.690982	1.55E-07	4.97E-08
ACADL	-6.159303	8.689348	-0.755167	9.23E-07	2.91E-08	-4.711778	3.23251	-1.915758	0.00014	8.76E-06	-6.737079	14.09168	-0.938865	2.24E-10	5.38E-11
ACADVL	-7.848134	15.87166	-0.947028	2.44E-09	1.77E-11	-1.788253	-5.054091	-0.412815	0.143	0.0771	-1.169881	-6.739608	-0.118582	0.299193	0.242717
ACAT1	-6.156403	8.677413	-0.81608	9.33E-07	2.94E-08	-4.959129	4.173677	-0.966053	6.67E-05	3.26E-06	-7.74543	20.56461	-0.937338	4.19E-13	7.31E-14
ACAT2	0.306352	-6.658347	0.013506	0.846	0.76	-3.181948	-1.851198	-0.787991	0.00826	0.002	-1.835719	-5.745768	-0.238999	0.09315	0.067111
ACBD3	6.225867	8.963858	0.465833	7.32E-07	2.19E-08	5.456154	6.144132	1.040078	1.43E-05	4.14E-07	8.835119	28.30302	0.819689	2.20E-16	2.82E-17
ACE	-1.273693	-5.904112	-0.037174	0.355	0.207	1.3658	-5.697574	0.117674	0.273	0.175	6.601706	13.27708	0.431286	5.00E-10	1.24E-10
ACOT11	-2.643861	-3.366256	-0.217405	0.0394	0.00989	-3.817703	0.090769	-0.25506	0.00166	0.000245	2.022587	-5.388876	0.06574	0.063525	0.043754
ACOT12	-2.935914	-2.627692	-0.688687	0.0206	0.00436	-3.104686	-2.068405	-1.681676	0.00991	0.00254	-5.290652	6.107019	-1.135338	5.78E-07	1.97E-07
ACOT13	-1.383991	-5.761125	-0.207718	0.311	0.17	-3.057886	-2.19788	-0.705721	0.0111	0.00293	1.600377	-6.146782	0.146558	0.146327	0.110272
ACOT4	-1.619073	-5.418926	-0.187809	0.229	0.109	-2.218001	-4.236187	-0.535197	0.0663	0.029	0.382692	-7.348901	0.039464	0.747373	0.702143
ACOT6	-1.253471	-5.929094	-0.050873	0.364	0.214	-0.695012	-6.375156	-0.132824	0.601	0.489	0.94622	-6.975347	0.023513	0.403954	0.344585
ACOT7	2.728049	-3.159867	0.250951	0.0329	0.00786	0.955944	-6.162892	0.162211	0.458	0.342	5.912108	9.339313	0.584803	2.35E-08	7.04E-09
ACOT8	5.104162	4.495709	0.299837	3.50E-05	2.26E-06	2.422703	-3.79126	0.44052	0.0442	0.0174	11.82839	52.79866	0.70779	1.55E-26	4.91E-28
ACOT9	6.957081	12.03485	0.735415	5.43E-08	9.20E-10	3.81348	0.077006	0.514599	0.00168	0.000249	8.484776	25.73621	0.627974	2.65E-15	3.81E-16
ACOX2	-3.408578	-1.30449	-0.558296	0.00635	0.00103	-3.559663	-0.729575	-1.078124	0.00324	0.000592	-5.155018	5.443026	-0.883296	1.11E-06	3.92E-07
ACSL1	-6.171249	8.738545	-1.065241	8.84E-07	2.76E-08	-4.368706	1.975947	-1.40169	0.000374	3.30E-05	-8.490376	25.77667	-1.674648	2.56E-15	3.66E-16
ACSL3	6.498423	10.09747	0.518727	2.79E-07	6.79E-09	2.464613	-3.695908	0.567846	0.0406	0.0156	5.59193	7.635509	0.582919	1.29E-07	4.07E-08
ACSL4	5.485504	5.972873	1.627564	9.76E-06	4.85E-07	4.405066	2.106278	3.469899	0.000336	2.87E-05	7.706099	20.29923	2.343952	5.36E-13	9.58E-14
ACSS1	3.016698	-2.412427	0.319288	0.017	0.00344	-3.459424	-1.036658	-0.299651	0.00418	0.000825	4.610281	2.93083	0.606533	1.35E-05	5.36E-06
ACSS2	4.552718	2.456408	0.322987	0.000214	1.90E-05	0.971152	-6.148488	0.255454	0.45	0.334	1.831958	-5.7526	0.240011	0.09374	0.067671
ADH4	-5.776937	7.132824	-2.252227	3.55E-06	1.46E-07	-5.200632	5.118618	-1.496694	3.21E-05	1.21E-06	-8.183887	23.59035	-3.425785	2.21E-14	3.37E-15
ADIPOQ	-3.492474	-1.05418	-0.190021	0.00507	0.000789	-4.778104	3.482106	-0.318485	0.000115	6.74E-06	0.69579	-7.180381	0.022293	0.546832	0.486948
AGO1	3.082753	-2.23299	0.194963	0.0145	0.00283	3.460624	-1.033023	0.773948	0.00417	0.000822	7.535309	19.15866	0.496945	1.66E-12	3.06E-13
AGO2	1.611128	-5.431313	0.220787	0.231	0.111	5.367116	5.783929	1.408862	1.89E-05	6.04E-07	9.9424	36.86473	0.800155	5.87E-20	4.82E-21

AGO3	2.243291	-4.27262	0.13531	0.0858	0.0277	1.45827	-5.570724	0.262913	0.239	0.148	2.566989	-4.156813	0.090556	0.017039	0.010607
AGO4	1.329964	-5.83258	0.069291	0.332	0.187	3.078145	-2.142028	0.481066	0.0106	0.00275	3.474679	-1.47802	0.229017	0.001086	0.000565
AGPAT2	-4.115639	0.936529	-0.525215	0.000837	9.45E-05	-4.861658	3.799459	-1.001738	8.93E-05	4.83E-06	-3.443758	-1.581871	-0.566593	0.001201	0.000632
AGT	0.05124	-6.703833	0.006207	0.976	0.959	-1.749267	-5.120264	-0.256608	0.152	0.0836	1.200041	-6.704029	0.178593	0.286033	0.230805
AGTR1	-5.513617	6.083666	-0.679298	8.88E-06	4.33E-07	-2.762965	-2.976485	-1.025882	0.0216	0.00693	-6.682988	13.7646	-1.067131	3.09E-10	7.52E-11
AIFM1	0.743918	-6.430017	0.031011	0.61	0.459	0.438763	-6.518883	0.084443	0.752	0.662	2.661858	-3.913072	0.213907	0.013211	0.008071
AKR1C1	0.301474	-6.659825	0.011902	0.848	0.764	-0.836829	-6.267987	-0.428336	0.521	0.405	2.170787	-5.081747	0.51257	0.045398	0.030511
AKR1C3	10.61365	27.86072	1.37607	1.04E-13	7.67E-17	4.752396	3.385117	1.398856	0.000125	7.46E-06	12.48907	58.71419	1.947193	7.26E-29	1.26E-30
AKR1C4	4.450229	2.091764	0.727134	0.000295	2.79E-05	-1.53293	-5.462529	-0.849316	0.214	0.129	-2.662668	-3.910956	-0.682888	0.013211	0.008052
AKR1D1	-7.384686	13.86686	-0.68852	1.23E-08	1.39E-10	-3.875755	0.28105	-2.716357	0.00142	2.00E-04	-8.183955	23.59084	-2.294714	2.21E-14	3.37E-15
ALB	-2.179547	-4.404921	-0.123744	0.096	0.0323	-3.908408	0.388976	-1.532197	0.00131	0.000178	-6.618495	13.37738	-1.817531	4.54E-10	1.12E-10
ALCAM	1.256993	-5.924769	0.128725	0.362	0.212	2.257814	-4.152386	0.514821	0.0614	0.0263	6.841168	14.72705	0.781349	1.20E-10	2.81E-11
ALDOC	-0.49846	-6.581379	-0.126473	0.743	0.62	-0.221354	-6.590053	-0.077194	0.879	0.825	-1.034945	-6.887762	-0.216468	0.361137	0.301294
ALG12	-0.94417	-6.26292	-0.043023	0.506	0.348	3.074276	-2.152716	0.400946	0.0106	0.00278	7.124654	16.49657	0.571972	2.18E-11	4.62E-12
ALG6	5.750306	7.025816	0.515643	3.93E-06	1.63E-07	3.629282	-0.512423	0.577826	0.00271	0.000469	11.4683	49.6413	0.668217	3.06E-25	1.19E-26
ALG9	-0.145199	-6.694626	-0.01271	0.93	0.885	3.078145	-2.142029	0.525321	0.0106	0.00275	5.789744	8.678626	0.28504	4.50E-08	1.39E-08
ALMS1	2.844897	-2.864612	0.105798	0.0255	0.00566	7.904447	16.88959	1.314244	1.92E-09	5.96E-12	8.609351	26.64065	0.441806	1.12E-15	1.52E-16
AMACR	-0.209728	-6.683204	-0.039443	0.898	0.834	-1.04306	-6.077371	-0.092451	0.414	0.3	2.170292	-5.082808	0.279271	0.045398	0.030549
ANGPTL3	-4.481542	2.202657	-0.590619	0.000268	2.49E-05	-1.873322	-4.904997	-0.825189	0.124	0.0642	-4.658209	3.141784	-1.059412	1.09E-05	4.30E-06
ANGPTL8	0.399254	-6.625695	0.117218	0.796	0.691	0.750827	-6.335315	0.389984	0.568	0.455	1.615028	-6.1234	0.481951	0.142338	0.107061
ANTXR2	-4.326515	1.658183	-0.555858	0.000433	4.41E-05	-4.464683	2.32147	-1.046754	0.000283	2.29E-05	-6.376903	11.95408	-0.594043	1.85E-09	4.80E-10
ANXA1	1.243252	-5.941572	0.217514	0.368	0.217	0.347706	-6.554382	0.032658	0.806	0.729	2.304181	-4.787165	0.325535	0.033031	0.021703
ANXA13	-0.823184	-6.368531	-0.147542	0.568	0.413	-0.265252	-6.579452	-0.122275	0.853	0.791	2.409952	-4.541423	0.569439	0.025499	0.016387
ANXA2	7.794113	15.63722	1.12087	2.96E-09	2.25E-11	8.806387	21.07065	2.025587	5.27E-11	7.88E-14	10.31837	39.91497	1.567266	3.19E-21	2.21E-22
ANXA2P2	-3.307577	-1.599849	-0.161798	0.00824	0.00142	7.562898	15.32335	1.575771	7.80E-09	3.01E-11	7.090391	16.2797	0.817922	2.70E-11	5.76E-12
ANXA5	1.7987	-5.123786	0.341473	0.177	0.0759	3.435291	-1.109593	0.976732	0.00443	0.000893	5.161179	5.472857	0.832145	1.08E-06	3.81E-07
ANXA6	-0.203225	-6.684543	-0.017503	0.9	0.839	-4.275843	1.646271	-0.317027	0.000484	4.68E-05	0.395316	-7.343997	0.053049	0.74085	0.692812
APOA1	-2.006489	-4.746968	-0.182799	0.128	0.0482	-2.373888	-3.900513	-0.470671	0.0488	0.0197	-4.929917	4.374653	-1.739241	3.24E-06	1.19E-06
APOA2	-1.322453	-5.842299	-0.117709	0.335	0.19	0.303757	-6.568582	0.035468	0.831	0.762	0.44583	-7.322785	0.152549	0.705783	0.655951
APOA4	-2.314672	-4.120504	-0.608761	0.0755	0.0232	-2.521394	-3.56445	-0.924914	0.0361	0.0134	-1.830656	-5.754964	-0.705947	0.093822	0.067866
APOA5	-6.884003	11.72401	-1.764124	6.96E-08	1.27E-09	-7.592993	15.46083	-2.550573	6.87E-09	2.61E-11	-6.132668	10.55954	-1.747592	7.22E-09	2.01E-09
APOB	-3.804686	-0.084708	-0.2597	0.0021	0.000279	-1.353589	-5.71373	-0.128201	0.278	0.179	-3.160387	-2.492596	-0.601496	0.003008	0.001691
APOBR	0.390217	-6.629248	0.019127	0.801	0.697	-2.914678	-2.584104	-0.211269	0.0154	0.00448	1.685197	-6.008495	0.141871	0.124435	0.092699
APOC1	-2.430255	-3.865437	-0.154855	0.0605	0.0174	-1.797151	-5.038798	-0.198584	0.141	0.0756	-1.973228	-5.486443	-0.411929	0.070012	0.04913
APOC2	-1.707783	-5.276765	-0.188637	0.202	0.0916	-1.759295	-5.103373	-0.243254	0.15	0.0819	6.162971	10.73011	0.888795	6.12E-09	1.69E-09
APOC3	-3.552374	-0.87276	-0.685293	0.00431	0.000649	-2.921882	-2.565037	-0.471671	0.0151	0.00439	-4.369321	1.900218	-1.402099	3.81E-05	1.58E-05
APOD	1.750264	-5.206198	0.211987	0.19	0.084	-0.128881	-6.606129	-0.026122	0.93	0.898	3.472631	-1.484925	0.409006	0.001091	0.000569
APOE	0.812309	-6.377329	0.044221	0.573	0.419	1.293053	-5.791764	0.224838	0.301	0.199	2.319961	-4.751185	0.387489	0.031834	0.020825
APOF	-7.726858	15.34558	-2.387209	3.71E-09	3.04E-11	-4.739121	3.335155	-3.145736	0.00013	7.86E-06	-11.67222	51.42328	-3.399298	5.46E-26	1.97E-27
APOH	-3.339118	-1.508325	-0.447935	0.0076	0.00129	-2.09389	-4.488811	-0.561032	0.0836	0.039	-3.570573	-1.150386	-0.981268	0.000791	0.000398
APOL1	-2.582475	-3.513342	-0.191384	0.0447	0.0117	-3.404756	-1.201313	-0.856569	0.00478	0.000986	-4.083771	0.744429	-0.605506	0.000117	5.31E-05
APOM	-2.97565	-2.522388	-0.491358	0.0188	0.00388	-1.577102	-5.396106	-0.751657	0.201	0.118	-1.557119	-6.214594	-0.39395	0.156804	0.120201

APOO	1.775177	-5.164068	0.093519	0.183	0.0797	2.618165	-3.334445	0.521524	0.0293	0.0104	9.179328	30.89358	0.730472	1.79E-17	2.04E-18
APTX	-0.691963	-6.466991	-0.036014	0.638	0.491	1.692975	-5.213405	0.171067	0.167	0.0939	9.192964	30.99757	0.369331	1.65E-17	1.83E-18
AQP1	1.386137	-5.758232	0.109714	0.31	0.17	1.508028	-5.499188	0.480848	0.222	0.135	0.561802	-7.264458	0.116342	0.625965	0.574553
AQP8	3.753389	-0.247975	0.477903	0.00243	0.000332	0.450178	-6.513855	0.169249	0.744	0.654	4.649533	3.103449	0.894011	1.13E-05	4.47E-06
ARL4C	1.983773	-4.78998	0.142497	0.133	0.0508	-3.385664	-1.25834	-0.178544	0.00502	0.00105	-0.635852	-7.220203	-0.102518	0.584141	0.525222
ARL6	-1.495521	-5.605079	-0.054809	0.269	0.139	1.832882	-4.976674	0.209084	0.132	0.0701	3.291366	-2.08086	0.142983	0.001981	0.001082
ARV1	4.605	2.644	0.484	0.000	0.000	2.634	-3.295	0.420	0.028	0.010	10.541	41.751	0.886	0.000	0.000
ASS1	-8.539	18.880	-1.383	0.000	0.000	-7.810	16.456	-1.930	0.000	0.000	-8.964	29.265	-1.598	0.000	0.000
ATAD3A	-2.280	-4.195	-0.180	0.080	0.025	-0.717	-6.360	-0.060	0.588	0.475	6.804	14.499	0.638	0.000	0.000
ATF3	-5.304	5.263	-1.171	0.000	0.000	-4.485	2.396	-0.394	0.000	0.000	-7.731	20.465	-1.141	0.000	0.000
ATF5	-6.073	8.336	-1.535	0.000	0.000	-6.094	8.804	-0.542	0.000	0.000	-5.476	7.037	-1.652	0.000	0.000
ATP6AP1	5.477	5.938	0.454	0.000	0.000	6.147	9.030	0.920	0.000	0.000	15.277	85.063	1.270	0.000	0.000
ATXN2	-0.952	-6.256	-0.057	0.502	0.344	4.357	1.933	0.534	0.000	0.000	10.557	41.890	0.542	0.000	0.000
AVPR1A	0.533	-6.564	0.025	0.724	0.596	-5.565	6.590	-1.049	0.000	0.000	-9.344	32.156	-2.005	0.000	0.000
AXIN2	1.786	-5.145	0.343	0.180	0.078	1.403	-5.648	0.886	0.259	0.164	3.732	-0.580	0.618	0.000	0.000
B4GALT1	-4.549	2.443	-0.502	0.000	0.000	-2.821	-2.829	-0.257	0.019	0.006	-1.106	-6.812	-0.106	0.328	0.269
BAAT	-3.442	-1.205	-0.597	0.006	0.001	-3.008	-2.333	-1.153	0.012	0.003	-2.135	-5.157	-0.453	0.049	0.033
BBIP1	3.727	-0.332	0.128	0.003	0.000	1.863	-4.923	0.178	0.126	0.066	6.406	12.123	0.221	0.000	0.000
BBS1	-1.333	-5.829	-0.086	0.331	0.187	-3.303	-1.502	-0.229	0.006	0.001	6.269	11.332	0.037	0.000	0.000
BBS10	5.553	6.240	0.428	0.000	0.000	2.142	-4.392	0.357	0.077	0.035	3.737	-0.562	0.319	0.000	0.000
BBS12	0.726	-6.443	0.026	0.619	0.470	2.288	-4.088	0.377	0.058	0.025	4.160	1.044	0.168	0.000	0.000
BBS2	1.894	-4.956	0.183	0.153	0.062	2.037	-4.600	0.423	0.093	0.045	5.072	5.046	0.405	0.000	0.000
BBS4	1.951	-4.851	0.142	0.140	0.055	3.859	0.227	0.660	0.001	0.000	10.764	43.618	0.782	0.000	0.000
BBS5	-1.386	-5.758	-0.060	0.310	0.170	-0.289	-6.573	-0.040	0.840	0.773	2.003	-5.427	0.099	0.066	0.046
BBS7	4.445	2.073	0.151	0.000	0.000	1.697	-5.207	0.253	0.166	0.093	5.997	9.806	0.346	0.000	0.000
BBS9	3.256	-1.748	0.152	0.009	0.002	-1.835	-4.973	-0.137	0.132	0.070	3.500	-1.392	0.148	0.001	0.001
BCL2L11	1.992	-4.775	0.078	0.131	0.050	-5.209	5.150	-0.317	0.000	0.000	3.348	-1.898	0.346	0.002	0.001
BMP2	2.677	-3.286	0.378	0.037	0.009	1.064	-6.055	0.456	0.404	0.290	2.683	-3.857	0.372	0.013	0.008
BMP5	-3.430	-1.242	-0.107	0.006	0.001	-7.066	13.072	-0.621	0.000	0.000	-9.403	32.613	-0.489	0.000	0.000
BTK	1.051	-6.158	0.102	0.453	0.297	-0.665	-6.395	-0.102	0.618	0.507	-0.251	-7.391	-0.019	0.839	0.802
CACNA1H	-4.393	1.889	-0.386	0.000	0.000	-3.358	-1.340	-0.246	0.005	0.001	0.902	-7.016	0.185	0.426	0.367
CARD11	-2.676	-3.288	-0.174	0.037	0.009	-2.539	-3.522	-0.137	0.035	0.013	1.690	-6.000	0.161	0.124	0.092
CARM1	1.320	-5.846	0.109	0.336	0.191	5.199	5.112	0.725	0.000	0.000	6.342	11.749	0.664	0.000	0.000
CASP9	-1.865	-5.008	-0.118	0.160	0.066	-2.303	-4.056	-0.131	0.056	0.024	3.787	-0.382	0.296	0.000	0.000
CAV1	5.244	5.033	0.538	0.000	0.000	-0.659	-6.399	-0.174	0.621	0.512	5.358	6.444	0.694	0.000	0.000
CAV2	2.566	-3.553	0.362	0.046	0.012	-3.031	-2.271	-0.277	0.012	0.003	0.454	-7.319	0.078	0.700	0.650
CAV3	-0.506	-6.578	-0.013	0.739	0.614	-4.209	1.413	-0.256	0.001	0.000	0.553	-7.270	0.003	0.632	0.581
CBS	-4.731	3.103	-1.059	0.000	0.000	-4.255	1.572	-1.470	0.001	0.000	-2.008	-5.417	-0.183	0.065	0.045
CCDC115	-1.069	-6.140	-0.058	0.445	0.289	0.673	-6.390	0.090	0.614	0.503	4.664	3.167	0.361	0.000	0.000
CD24	4.927	3.827	0.863	0.000	0.000	3.330	-1.423	1.545	0.006	0.001	4.735	3.486	1.565	0.000	0.000
CD36	2.432	-3.861	0.515	0.060	0.017	0.482	-6.499	0.086	0.726	0.631	2.338	-4.710	0.424	0.030	0.020
CD48	0.967	-6.242	0.129	0.494	0.337	-0.061	-6.613	-0.005	0.968	0.951	-0.261	-7.388	-0.023	0.835	0.794

CD55	0.390	-6.629	0.038	0.801	0.697	-1.182	-5.926	-0.524	0.348	0.240	0.623	-7.228	0.091	0.592	0.534
CD79A	-1.956	-4.843	-0.170	0.139	0.054	-0.897	-6.217	-0.135	0.488	0.372	-0.871	-7.044	-0.154	0.444	0.384
CD81	-4.540	2.409	-0.402	0.000	0.000	-2.727	-3.066	-0.594	0.023	0.008	-4.532	2.592	-0.598	0.000	0.000
CD9	3.904	0.234	0.416	0.002	0.000	-3.557	-0.739	-0.321	0.003	0.001	2.231	-4.951	0.318	0.039	0.026
CDH13	8.537	18.869	0.690	0.000	0.000	3.222	-1.737	0.554	0.007	0.002	7.531	19.128	0.327	0.000	0.000
CEL	1.309	-5.860	0.048	0.341	0.194	1.769	-5.086	0.314	0.147	0.080	4.079	0.726	0.376	0.000	0.000
CEP19	-5.667	6.692	-0.766	0.000	0.000	1.326	-5.750	0.135	0.288	0.188	5.763	8.538	0.314	0.000	0.000
CEP290	3.995	0.534	0.169	0.001	0.000	4.404	2.102	0.561	0.000	0.000	7.080	16.215	0.221	0.000	0.000
CETP	-10.129	25.784	-1.660	0.000	0.000	-7.977	17.225	-2.314	0.000	0.000	-14.598	78.480	-1.732	0.000	0.000
CFLAR	-5.101	4.483	-0.437	0.000	0.000	-3.199	-1.801	-0.187	0.008	0.002	3.505	-1.376	0.214	0.001	0.001
CH25H	-2.649	-3.353	-0.316	0.039	0.010	0.652	-6.404	0.244	0.625	0.516	-1.817	-5.780	-0.243	0.097	0.070
CHD9	-3.315	-1.578	-0.370	0.008	0.001	3.159	-1.915	0.468	0.009	0.002	1.861	-5.700	0.114	0.089	0.064
CHKA	-2.589	-3.499	-0.099	0.044	0.012	3.498	-0.920	0.938	0.004	0.001	10.222	39.123	1.230	0.000	0.000
CIDEB	-5.530	6.148	-1.132	0.000	0.000	-5.170	4.997	-1.744	0.000	0.000	0.000	-7.422	0.000	1.000	1.000
CLIP3	0.871	-6.328	0.102	0.543	0.386	-2.981	-2.407	-0.218	0.013	0.004	3.056	-2.808	0.299	0.004	0.002
CLN3	7.458	14.183	0.815	0.000	0.000	5.400	5.918	0.933	0.000	0.000	5.682	8.109	0.035	0.000	0.000
CLU	-3.989	0.515	-0.302	0.001	0.000	-4.137	1.162	-1.427	0.001	0.000	-3.969	0.301	-0.610	0.000	0.000
COG4	-3.308	-1.598	-0.182	0.008	0.001	3.976	0.615	0.428	0.001	0.000	10.521	41.586	0.777	0.000	0.000
COLEC12	3.759	-0.229	0.553	0.002	0.000	1.629	-5.316	0.555	0.185	0.107	3.609	-1.018	0.344	0.001	0.000
COMT	5.034	4.231	0.333	0.000	0.000	-3.695	-0.304	-0.382	0.002	0.000	-3.122	-2.611	-0.402	0.003	0.002
CPEB2	-1.578	-5.482	-0.123	0.242	0.119	-1.084	-6.035	-0.235	0.394	0.281	-0.599	-7.243	-0.059	0.604	0.550
CREB3L3	-1.504	-5.593	-0.325	0.267	0.137	0.843	-6.263	0.250	0.518	0.401	-2.753	-3.671	-0.593	0.010	0.006
CREBBP	-1.742	-5.221	-0.073	0.192	0.086	-2.207	-4.258	-0.171	0.068	0.030	4.239	1.363	0.329	0.000	0.000
CRH	-1.076	-6.132	-0.037	0.442	0.285	-3.448	-1.072	-0.270	0.004	0.001	0.766	-7.129	0.030	0.504	0.444
CRP	-3.952	0.393	-1.816	0.001	0.000	-1.282	-5.806	-1.423	0.306	0.203	-3.543	-1.247	-1.878	0.001	0.000
CTNNB1	5.927	7.739	0.449	0.000	0.000	4.537	2.585	0.567	0.000	0.000	6.011	9.881	0.582	0.000	0.000
CXCL16	-1.669	-5.340	-0.301	0.213	0.099	-0.180	-6.598	-0.059	0.902	0.857	1.340	-6.527	0.227	0.228	0.181
CYB5R1	2.661	-3.324	0.273	0.038	0.009	3.633	-0.501	0.639	0.003	0.000	12.086	55.087	1.122	0.000	0.000
CYB5R2	-2.584	-3.509	-0.305	0.045	0.012	0.512	-6.484	0.093	0.708	0.610	1.412	-6.429	0.073	0.203	0.159
CYB5R3	2.258	-4.242	0.188	0.084	0.027	2.441	-3.750	0.447	0.043	0.017	4.271	1.493	0.414	0.000	0.000
CYP11A1	-0.895	-6.308	-0.118	0.531	0.374	0.542	-6.469	0.163	0.690	0.589	0.945	-6.976	0.139	0.404	0.345
CYP11B1	-1.184	-6.012	-0.039	0.393	0.240	-1.807	-5.023	-0.097	0.138	0.074	0.587	-7.250	0.004	0.610	0.558
CYP11B2	-0.228	-6.679	-0.008	0.888	0.820	-1.089	-6.030	-0.183	0.392	0.279	0.616	-7.233	0.039	0.595	0.538
CYP17A1	2.291	-4.170	0.313	0.079	0.025	1.373	-5.688	0.883	0.270	0.173	4.983	4.624	1.276	0.000	0.000
CYP19A1	-1.550	-5.525	-0.039	0.251	0.125	-3.998	0.688	-0.198	0.001	0.000	2.118	-5.193	0.176	0.051	0.035
CYP1A1	-4.889	3.687	-1.181	0.000	0.000	-3.368	-1.312	-2.231	0.005	0.001	-3.877	-0.048	-1.233	0.000	0.000
CYP1B1	2.074	-4.617	0.255	0.115	0.041	1.096	-6.022	0.442	0.388	0.276	3.014	-2.933	0.516	0.005	0.003
CYP27A1	-1.762	-5.186	-0.238	0.187	0.082	-1.251	-5.844	-0.328	0.319	0.214	-3.612	-1.008	-0.691	0.001	0.000
CYP27B1	0.255	-6.673	0.016	0.873	0.799	1.217	-5.885	0.228	0.333	0.227	5.025	4.819	0.300	0.000	0.000
CYP2R1	7.556	14.605	0.490	0.000	0.000	4.787	3.514	0.825	0.000	0.000	12.654	60.210	0.785	0.000	0.000
CYP39A1	-10.335	26.671	-0.986	0.000	0.000	-4.744	3.352	-2.126	0.000	0.000	-11.787	52.431	-2.372	0.000	0.000
CYP3A4	-7.632	14.933	-3.412	0.000	0.000	-6.213	9.313	-3.103	0.000	0.000	-8.938	29.067	-4.540	0.000	0.000

CYP3A43	-9.119	21.409	-0.913	0.000	0.000	-7.165	13.517	-2.153	0.000	0.000	-6.425	12.232	-0.879	0.000	0.000
CYP3A5	-4.361	1.777	-0.949	0.000	0.000	-2.309	-4.042	-1.118	0.056	0.023	-1.193	-6.712	-0.251	0.288	0.233
CYP46A1	-1.218	-5.972	-0.041	0.378	0.227	-4.256	1.575	-0.448	0.001	0.000	0.859	-7.054	0.012	0.450	0.391
CYP7A1	3.632	-0.627	0.491	0.003	0.000	0.767	-6.323	0.712	0.560	0.445	3.744	-0.535	1.249	0.000	0.000
CYP7B1	-0.853	-6.344	-0.028	0.552	0.396	-1.915	-4.830	-0.798	0.115	0.059	-3.136	-2.568	-0.454	0.003	0.002
CYP8B1	-3.489	-1.064	-1.160	0.005	0.001	-3.355	-1.348	-2.705	0.005	0.001	-6.749	14.162	-2.580	0.000	0.000
DAG1	-1.982	-4.794	-0.073	0.133	0.051	0.377	-6.544	0.051	0.788	0.707	7.356	17.982	0.704	0.000	0.000
DBP	0.645	-6.498	0.060	0.663	0.521	-2.240	-4.189	-0.147	0.064	0.028	7.629	19.779	0.752	0.000	0.000
DDIT4	-0.836	-6.358	-0.194	0.561	0.405	0.287	-6.573	0.116	0.841	0.775	3.850	-0.147	0.719	0.000	0.000
DEAF1	4.411	1.954	0.219	0.000	0.000	-2.568	-3.454	-0.167	0.033	0.012	11.928	53.679	0.988	0.000	0.000
DGAT1	1.366	-5.785	0.155	0.318	0.176	-1.782	-5.065	-0.431	0.144	0.078	6.108	10.422	0.665	0.000	0.000
DGKQ	3.924	0.301	0.336	0.001	0.000	4.646	2.986	0.774	0.000	0.000	11.660	51.315	0.969	0.000	0.000
DHCR24	0.077	-6.702	0.010	0.963	0.939	1.635	-5.307	0.295	0.183	0.106	2.849	-3.408	0.333	0.008	0.005
DHCR7	2.396	-3.942	0.473	0.065	0.019	1.024	-6.097	0.307	0.424	0.309	3.304	-2.041	0.485	0.002	0.001
DHRS11	3.354	-1.466	0.242	0.007	0.001	1.781	-5.067	0.425	0.145	0.078	8.038	22.570	0.573	0.000	0.000
DIO1	-1.202	-5.991	-0.248	0.385	0.233	-0.696	-6.374	-0.455	0.600	0.488	-2.664	-3.906	-0.729	0.013	0.008
DKK3	3.084	-2.230	0.736	0.015	0.003	2.380	-3.887	1.155	0.048	0.019	3.687	-0.740	0.550	0.001	0.000
DLK1	2.224	-4.314	0.604	0.089	0.029	-4.525	2.540	-0.355	0.000	0.000	2.496	-4.333	0.842	0.021	0.013
DYRK1B	3.382	-1.383	0.270	0.007	0.001	-1.347	-5.722	-0.107	0.280	0.181	7.324	17.775	0.767	0.000	0.000
EBP	-0.294	-6.662	-0.053	0.852	0.770	-1.337	-5.736	-0.354	0.284	0.185	0.916	-7.003	0.137	0.419	0.360
ECH1	-4.134	0.998	-0.567	0.001	0.000	-1.626	-5.320	-0.272	0.186	0.107	-1.145	-6.769	-0.130	0.310	0.253
EDN1	-2.020	-4.722	-0.225	0.126	0.047	-2.673	-3.200	-0.570	0.026	0.009	-0.618	-7.232	-0.071	0.594	0.537
EEPD1	4.258	1.422	0.388	0.001	0.000	-2.994	-2.371	-0.179	0.013	0.004	6.890	15.030	0.695	0.000	0.000
EGF	1.302	-5.869	0.053	0.344	0.197	0.469	-6.505	0.085	0.733	0.640	3.195	-2.384	0.194	0.003	0.002
EGFR	-2.855	-2.838	-0.382	0.025	0.006	-3.386	-1.257	-0.808	0.005	0.001	-1.315	-6.560	-0.173	0.238	0.189
EHD1	-2.258	-4.242	-0.208	0.084	0.027	1.778	-5.072	0.173	0.145	0.079	6.112	10.444	0.476	0.000	0.000
EIF4E	-2.368	-4.004	-0.077	0.068	0.020	-4.056	0.884	-0.323	0.001	0.000	3.053	-2.819	0.130	0.004	0.002
ELOVL2	1.790	-5.139	0.373	0.179	0.077	0.735	-6.347	0.303	0.577	0.464	2.975	-3.049	0.635	0.005	0.003
ELOVL3	-2.516	-3.668	-0.132	0.051	0.014	0.471	-6.504	0.046	0.732	0.639	3.235	-2.260	0.201	0.002	0.001
ELOVL4	-0.316	-6.655	-0.016	0.841	0.753	-0.779	-6.314	-0.062	0.553	0.438	2.481	-4.369	0.140	0.021	0.013
ELOVL5	5.092	4.448	0.755	0.000	0.000	2.670	-3.208	0.451	0.026	0.009	5.213	5.727	0.644	0.000	0.000
ELOVL6	-2.543	-3.606	-0.490	0.048	0.013	1.579	-5.394	0.703	0.200	0.118	-1.838	-5.742	-0.271	0.093	0.067
EMD	1.046	-6.163	0.066	0.456	0.299	1.228	-5.871	0.187	0.328	0.222	11.014	45.732	0.903	0.000	0.000
ENPP7	2.281	-4.193	0.303	0.080	0.025	0.271	-6.578	0.082	0.850	0.787	0.331	-7.367	0.066	0.786	0.740
EP300	3.940	0.352	0.369	0.001	0.000	3.113	-2.046	0.439	0.010	0.002	3.551	-1.218	0.348	0.001	0.000
EPB41L4A	-3.085	-2.227	-0.148	0.015	0.003	-1.635	-5.307	-0.519	0.183	0.106	-1.017	-6.906	-0.046	0.370	0.310
EPHX2	-4.525	2.356	-0.952	0.000	0.000	-4.601	2.819	-1.649	0.000	0.000	-7.847	21.256	-1.259	0.000	0.000
ERLIN1	-5.927	7.738	-0.647	0.000	0.000	-5.039	4.485	-0.907	0.000	0.000	-7.361	18.015	-0.745	0.000	0.000
ERLIN2	-3.207	-1.887	-0.380	0.011	0.002	-2.871	-2.698	-0.684	0.017	0.005	-0.960	-6.962	-0.097	0.398	0.338
ERRFI1	-6.830	11.493	-1.111	0.000	0.000	-3.623	-0.533	-1.647	0.003	0.000	-6.012	9.887	-1.100	0.000	0.000
ESR1	-10.923	29.177	-1.226	0.000	0.000	-7.970	17.191	-0.637	0.000	0.000	-13.073	64.060	-1.129	0.000	0.000
ESR2	-2.776	-3.039	-0.077	0.030	0.007	-3.291	-1.537	-0.242	0.006	0.001	2.450	-4.446	0.020	0.023	0.015

ETHE1	1.005	-6.205	0.050	0.475	0.318	-3.581	-0.663	-0.574	0.003	0.001	-0.945	-6.976	-0.089	0.404	0.345
FABP2	-0.574	-6.541	-0.022	0.701	0.567	-2.943	-2.509	-0.232	0.014	0.004	1.494	-6.310	0.034	0.177	0.136
FABP5	5.788	7.178	0.870	0.000	0.000	6.663	11.279	2.305	0.000	0.000	7.446	18.571	0.911	0.000	0.000
FADS2	0.643	-6.499	0.079	0.664	0.522	1.695	-5.210	0.601	0.167	0.094	2.500	-4.323	0.615	0.020	0.013
FAM107A	-0.092	-6.701	-0.014	0.956	0.927	-4.035	0.813	-0.431	0.001	0.000	-0.991	-6.932	-0.089	0.381	0.322
FASN	2.966	-2.549	0.619	0.019	0.004	-5.028	4.441	-0.374	0.000	0.000	7.818	21.057	1.484	0.000	0.000
FBXO32	5.224	4.953	0.288	0.000	0.000	2.989	-2.386	0.988	0.013	0.004	6.801	14.481	0.785	0.000	0.000
FBXO6	3.675	-0.493	0.414	0.003	0.000	2.019	-4.634	0.503	0.096	0.046	6.790	14.411	0.732	0.000	0.000
FDFT1	3.310	-1.593	0.530	0.008	0.001	1.652	-5.280	0.405	0.178	0.102	4.690	3.282	0.567	0.000	0.000
FDPS	2.234	-4.292	0.360	0.087	0.028	4.338	1.868	1.256	0.000	0.000	9.703	34.956	1.381	0.000	0.000
FDX1	-5.599	6.422	-0.387	0.000	0.000	-3.139	-1.972	-0.589	0.009	0.002	-1.793	-5.823	-0.154	0.102	0.074
FDXR	1.079	-6.129	0.057	0.441	0.284	2.922	-2.564	0.671	0.015	0.004	4.345	1.800	0.498	0.000	0.000
FECH	-1.937	-4.877	-0.109	0.143	0.056	-1.772	-5.082	-0.239	0.147	0.080	0.162	-7.409	0.012	0.904	0.872
FGF1	-1.143	-6.059	-0.083	0.411	0.256	-2.403	-3.835	-0.145	0.046	0.018	1.572	-6.192	0.106	0.153	0.117
FHL1	-2.269	-4.218	-0.267	0.082	0.026	-4.213	1.426	-0.891	0.001	0.000	-4.319	1.689	-0.553	0.000	0.000
FLCN	2.856	-2.837	0.136	0.025	0.005	-3.061	-2.189	-0.217	0.011	0.003	7.836	21.181	0.544	0.000	0.000
FLII	3.549	-0.882	0.299	0.004	0.001	3.591	-0.631	0.550	0.003	0.001	6.950	15.399	0.656	0.000	0.000
FLOT1	5.402	5.646	0.408	0.000	0.000	4.108	1.063	0.631	0.001	0.000	10.611	42.337	0.986	0.000	0.000
FLT3	-0.359	-6.641	-0.012	0.818	0.721	-2.939	-2.519	-0.245	0.015	0.004	-2.199	-5.021	-0.072	0.043	0.028
FLT4	-1.221	-5.968	-0.034	0.377	0.226	-2.854	-2.743	-0.186	0.018	0.005	2.870	-3.350	0.227	0.007	0.004
FMO5	-1.495	-5.605	-0.228	0.269	0.139	-1.281	-5.807	-0.424	0.306	0.203	-2.483	-4.366	-0.466	0.021	0.013
FOS	-8.967	20.746	-2.545	0.000	0.000	-4.204	1.395	-2.202	0.001	0.000	-12.100	55.208	-2.817	0.000	0.000
FOXO3	-2.199	-4.365	-0.203	0.093	0.031	-2.037	-4.600	-0.206	0.093	0.045	2.059	-5.314	0.194	0.059	0.040
GALK1	0.856	-6.341	0.129	0.551	0.395	-1.489	-5.527	-0.165	0.229	0.140	3.453	-1.550	0.520	0.001	0.001
GALNT2	1.143	-6.059	0.058	0.411	0.257	0.993	-6.127	0.224	0.439	0.323	2.232	-4.950	0.245	0.039	0.026
GGPS1	4.614	2.678	0.539	0.000	0.000	4.323	1.813	1.111	0.000	0.000	11.170	47.068	0.789	0.000	0.000
GHR	-7.574	14.682	-1.838	0.000	0.000	-5.352	5.724	-2.773	0.000	0.000	-11.338	48.510	-2.042	0.000	0.000
GHSR	-1.618	-5.420	-0.055	0.229	0.110	-5.091	4.686	-0.243	0.000	0.000	0.751	-7.140	0.007	0.513	0.453
GJB2	-3.709	-0.387	-0.584	0.003	0.000	-2.162	-4.353	-0.758	0.074	0.033	-4.580	2.799	-0.756	0.000	0.000
GLDC	-2.792	-3.000	-0.622	0.029	0.007	-2.390	-3.866	-1.128	0.047	0.019	-3.178	-2.440	-0.555	0.003	0.002
GNAI1	1.615	-5.425	0.088	0.230	0.110	3.362	-1.329	1.090	0.005	0.001	5.034	4.866	0.404	0.000	0.000
GNB3	0.244	-6.675	0.009	0.879	0.808	-3.372	-1.300	-0.245	0.005	0.001	3.245	-2.229	0.045	0.002	0.001
GPAM	-1.387	-5.757	-0.297	0.310	0.169	-0.337	-6.558	-0.133	0.811	0.737	1.056	-6.866	0.215	0.352	0.292
GPER1	-3.133	-2.094	-0.656	0.013	0.002	-2.597	-3.386	-0.713	0.031	0.011	-0.014	-7.422	-0.002	1.000	0.989
GPIHBP1	3.831	-0.001	0.416	0.002	0.000	1.306	-5.776	0.187	0.296	0.195	5.886	9.198	0.601	0.000	0.000
GPLD1	-2.040	-4.682	-0.321	0.121	0.045	-3.531	-0.817	-0.292	0.003	0.001	-2.380	-4.613	-0.514	0.028	0.018
GPR155	-1.128	-6.075	-0.031	0.418	0.263	1.544	-5.446	0.470	0.211	0.126	1.778	-5.850	0.136	0.105	0.076
GPR183	-0.668	-6.483	-0.026	0.651	0.506	-2.027	-4.620	-0.583	0.094	0.046	-2.155	-5.116	-0.301	0.047	0.032
GPS2	1.515	-5.576	0.104	0.262	0.134	2.907	-2.604	0.329	0.016	0.005	5.585	7.601	0.071	0.000	0.000
GPX8	2.268	-4.221	0.167	0.082	0.026	2.075	-4.527	0.603	0.087	0.041	4.832	3.923	0.517	0.000	0.000
GRAMD1A	5.561	6.273	0.754	0.000	0.000	3.755	-0.112	0.685	0.002	0.000	9.737	35.225	1.397	0.000	0.000
GRAMD1B	1.655	-5.363	0.075	0.218	0.102	1.016	-6.104	0.160	0.427	0.312	3.028	-2.893	0.300	0.005	0.003

GRAMD1C	-4.824	3.445	-0.430	0.000	0.000	-4.554	2.646	-1.736	0.000	0.000	-7.520	19.057	-0.689	0.000	0.000
GSK3A	1.173	-6.025	0.046	0.398	0.244	-2.187	-4.300	-0.156	0.070	0.031	11.963	53.996	0.853	0.000	0.000
GSTM1	-0.681	-6.475	-0.296	0.644	0.498	-1.626	-5.321	-0.720	0.186	0.107	-3.571	-1.148	-1.201	0.001	0.000
GSTM2	-3.095	-2.200	-0.143	0.014	0.003	-1.650	-5.283	-0.790	0.179	0.102	3.509	-1.361	0.150	0.001	0.000
GUSB	-1.634	-5.396	-0.149	0.224	0.106	-3.110	-2.053	-0.278	0.010	0.002	2.613	-4.040	0.234	0.015	0.009
H6PD	-1.528	-5.558	-0.200	0.258	0.131	1.513	-5.492	0.346	0.221	0.134	3.445	-1.576	0.436	0.001	0.001
HDAC3	-1.223	-5.966	-0.071	0.376	0.225	1.528	-5.470	0.152	0.216	0.130	9.925	36.724	0.600	0.000	0.000
HDLBP	2.476	-3.761	0.124	0.055	0.015	1.434	-5.604	0.238	0.248	0.155	6.021	9.935	0.421	0.000	0.000
HELZ2	1.890	-4.963	0.227	0.154	0.063	2.215	-4.242	0.285	0.067	0.029	3.943	0.201	0.382	0.000	0.000
HERC2	3.930	0.320	0.258	0.001	0.000	3.471	-1.000	0.432	0.004	0.001	7.349	17.935	0.448	0.000	0.000
HINT2	-4.834	3.481	-0.510	0.000	0.000	-2.953	-2.482	-0.538	0.014	0.004	7.379	18.131	0.081	0.000	0.000
HMGCL	-6.760	11.197	-0.958	0.000	0.000	-3.890	0.329	-0.889	0.001	0.000	-7.671	20.061	-0.848	0.000	0.000
HMGCR	2.565	-3.554	0.427	0.046	0.012	1.193	-5.913	0.352	0.344	0.236	3.978	0.335	0.512	0.000	0.000
HMGCS1	2.725	-3.167	0.568	0.033	0.008	2.045	-4.585	0.611	0.091	0.044	3.297	-2.063	0.592	0.002	0.001
HMGCS1	2.725	-3.167	0.568	0.033	0.008	2.045	-4.585	0.781	0.091	0.044	3.297	-2.063	0.592	0.002	0.001
HMGCS2	-3.251	-1.761	-0.564	0.010	0.002	-5.971	8.280	-2.112	0.000	0.000	-4.571	2.758	-1.161	0.000	0.000
HNF1A	3.177	-1.971	0.201	0.012	0.002	-1.439	-5.598	-0.142	0.246	0.154	4.763	3.608	0.497	0.000	0.000
HNRNPU	-5.591	6.391	-0.709	0.000	0.000	4.848	3.746	0.702	0.000	0.000	11.955	53.919	0.888	0.000	0.000
HPR	-2.274	-4.207	-0.543	0.081	0.026	-2.761	-2.981	-1.555	0.022	0.007	-3.322	-1.983	-0.993	0.002	0.001
HSD11B1	-3.407	-1.310	-1.203	0.006	0.001	-2.872	-2.696	-2.228	0.017	0.005	-5.793	8.695	-2.369	0.000	0.000
HSD11B2	2.772	-3.049	0.192	0.030	0.007	1.201	-5.903	0.260	0.340	0.233	5.941	9.499	0.699	0.000	0.000
HSD17B1	2.907	-2.704	0.125	0.022	0.005	3.467	-1.015	0.415	0.004	0.001	3.926	0.137	0.245	0.000	0.000
HSD17B10	-2.587	-3.503	-0.337	0.044	0.012	-3.561	-0.726	-0.596	0.003	0.001	-0.898	-7.020	-0.087	0.428	0.370
HSD17B11	-2.629	-3.401	-0.099	0.041	0.010	1.114	-6.002	0.216	0.379	0.268	1.107	-6.811	0.127	0.328	0.269
HSD17B12	-3.253	-1.757	-0.267	0.009	0.002	-3.149	-1.944	-0.170	0.009	0.002	-0.679	-7.192	-0.057	0.558	0.498
HSD17B2	-6.123	8.542	-1.262	0.000	0.000	-2.359	-3.933	-1.437	0.050	0.020	-4.312	1.661	-0.717	0.000	0.000
HSD17B3	-0.104	-6.700	-0.006	0.950	0.917	0.874	-6.237	0.225	0.501	0.384	1.692	-5.997	0.004	0.123	0.091
HSD17B4	-0.099	-6.700	-0.011	0.953	0.922	-0.651	-6.405	-0.128	0.626	0.517	0.004	-7.422	0.001	1.000	0.996
HSD17B6	-2.961	-2.562	-0.770	0.019	0.004	-2.225	-4.221	-1.002	0.065	0.029	-5.484	7.078	-1.576	0.000	0.000
HSD17B7	-4.690	2.951	-0.624	0.000	0.000	3.141	-1.967	0.569	0.009	0.002	8.147	23.330	0.815	0.000	0.000
HSD17B8	-2.762	-3.074	-0.512	0.031	0.007	-1.171	-5.939	-0.253	0.354	0.245	-0.612	-7.235	-0.080	0.597	0.541
HSD3B1	-1.161	-6.039	-0.055	0.403	0.249	-1.274	-5.816	-0.274	0.309	0.206	0.224	-7.397	0.020	0.856	0.823
HSD3B2	-1.875	-4.990	-0.069	0.158	0.065	-3.226	-1.725	-0.215	0.007	0.002	0.592	-7.247	0.021	0.608	0.555
HSD3B7	-1.649	-5.372	-0.212	0.219	0.103	0.320	-6.564	0.064	0.822	0.750	1.946	-5.539	0.249	0.074	0.052
HTT	-3.057	-2.303	-0.171	0.016	0.003	3.039	-2.249	0.308	0.012	0.003	10.450	41.000	0.701	0.000	0.000
IDI1	3.856	0.080	0.591	0.002	0.000	5.813	7.614	1.120	0.000	0.000	6.492	12.630	0.824	0.000	0.000
IDI2	-1.548	-5.528	-0.088	0.252	0.126	-5.124	4.815	-0.295	0.000	0.000	1.752	-5.894	0.005	0.110	0.080
IFT172	4.989	4.058	0.226	0.000	0.000	2.534	-3.536	0.283	0.035	0.013	7.651	19.932	0.362	0.000	0.000
IFT27	2.001	-4.757	0.117	0.129	0.049	2.746	-3.020	0.518	0.022	0.007	10.173	38.725	0.648	0.000	0.000
IFT74	0.456	-6.602	0.036	0.766	0.650	2.215	-4.243	0.520	0.067	0.029	5.394	6.625	0.293	0.000	0.000
IGF1R	0.808	-6.381	0.029	0.576	0.422	-3.182	-1.851	-0.204	0.008	0.002	1.730	-5.932	0.148	0.114	0.084
IKZF1	-0.860	-6.338	-0.055	0.549	0.393	-2.784	-2.924	-0.134	0.021	0.007	-0.775	-7.122	-0.051	0.499	0.439

IL12A	1.479	-5.629	0.058	0.275	0.143	-4.064	0.911	-0.235	0.001	0.000	3.862	-0.102	0.109	0.000	0.000
IL12RB1	1.278	-5.899	0.036	0.354	0.205	-2.981	-2.408	-0.213	0.013	0.004	0.994	-6.929	0.072	0.380	0.321
IL17A	-1.150	-6.050	-0.028	0.408	0.253	-5.545	6.505	-0.300	0.000	0.000	1.049	-6.873	0.007	0.355	0.295
IL17B	-0.244	-6.675	-0.008	0.879	0.808	0.948	-6.170	0.068	0.462	0.346	4.119	0.883	0.072	0.000	0.000
IL17C	-1.392	-5.750	-0.046	0.307	0.168	-4.339	1.869	-0.425	0.000	0.000	1.229	-6.669	0.006	0.273	0.220
IL17D	3.266	-1.721	0.390	0.009	0.002	1.479	-5.542	0.562	0.232	0.143	4.449	2.234	0.473	0.000	0.000
IL17F	-1.734	-5.233	-0.054	0.194	0.087	-1.660	-5.267	-0.124	0.176	0.100	2.085	-5.263	0.022	0.055	0.038
IL25	-3.157	-2.027	-0.154	0.012	0.002	-3.250	-1.656	-0.201	0.007	0.002	0.940	-6.981	0.008	0.406	0.348
INHBA	1.667	-5.343	0.061	0.214	0.100	-1.269	-5.822	-0.640	0.311	0.208	-2.534	-4.239	-0.294	0.019	0.012
INHBB	-0.801	-6.387	-0.114	0.580	0.426	0.233	-6.587	0.079	0.872	0.816	-0.363	-7.356	-0.063	0.762	0.717
INSIG1	-0.747	-6.428	-0.150	0.609	0.457	-1.626	-5.320	-0.645	0.186	0.107	-1.488	-6.318	-0.336	0.178	0.137
INSIG2	-2.248	-4.263	-0.266	0.085	0.027	-3.669	-0.389	-1.065	0.002	0.000	-0.611	-7.236	-0.074	0.597	0.542
IQSEC2	-1.666	-5.345	-0.057	0.214	0.100	-5.588	6.684	-0.521	0.000	0.000	2.094	-5.243	0.077	0.054	0.037
IRF5	1.957	-4.840	0.075	0.139	0.054	-4.283	1.672	-0.330	0.000	0.000	8.841	28.350	0.740	0.000	0.000
ISL1	-1.514	-5.579	-0.079	0.263	0.134	-1.928	-4.805	-0.100	0.112	0.057	0.835	-7.074	0.022	0.462	0.404
ITLN1	-7.345	13.697	-0.707	0.000	0.000	-4.521	2.526	-0.756	0.000	0.000	-10.793	43.863	-0.957	0.000	0.000
JAG1	3.027	-2.383	0.355	0.017	0.003	-3.921	0.432	-0.253	0.001	0.000	4.696	3.308	0.664	0.000	0.000
JAK2	1.164	-6.035	0.064	0.402	0.248	1.346	-5.724	0.223	0.280	0.182	1.388	-6.462	0.095	0.211	0.166
KDM1A	1.701	-5.287	0.156	0.204	0.093	1.418	-5.627	0.166	0.253	0.160	9.205	31.093	0.747	0.000	0.000
KDM1B	-2.259	-4.240	-0.067	0.084	0.027	2.580	-3.427	0.597	0.032	0.012	5.548	7.408	0.472	0.000	0.000
KDM3A	3.335	-1.520	0.170	0.008	0.001	3.680	-0.351	0.622	0.002	0.000	7.143	16.614	0.535	0.000	0.000
KDM4A	-1.399	-5.741	-0.097	0.305	0.166	2.066	-4.543	0.219	0.088	0.042	8.363	24.858	0.527	0.000	0.000
KIF12	-1.743	-5.219	-0.219	0.192	0.085	-2.422	-3.792	-0.312	0.044	0.017	2.565	-4.162	0.509	0.017	0.011
KLF9	-3.915	0.273	-0.618	0.002	0.000	-3.905	0.377	-0.334	0.001	0.000	-4.269	1.487	-0.675	0.000	0.000
KLK3	-1.433	-5.694	-0.051	0.292	0.156	-4.128	1.132	-0.283	0.001	0.000	1.592	-6.161	0.140	0.148	0.112
KPNB1	4.797	3.344	0.407	0.000	0.000	5.807	7.591	0.683	0.000	0.000	10.067	37.865	0.910	0.000	0.000
LAMTOR1	5.194	4.841	0.364	0.000	0.000	4.139	1.168	0.457	0.001	0.000	13.914	71.939	1.015	0.000	0.000
LAT	-3.102	-2.179	-0.157	0.014	0.003	1.046	-6.074	0.105	0.413	0.298	4.908	4.273	0.032	0.000	0.000
LAT2	1.370	-5.779	0.100	0.316	0.174	-1.591	-5.374	-0.129	0.196	0.115	1.964	-5.504	0.189	0.071	0.050
LAX1	-0.159	-6.692	-0.009	0.923	0.874	0.664	-6.396	0.113	0.619	0.508	-0.030	-7.422	-0.001	0.997	0.976
LBP	-1.172	-6.026	-0.310	0.398	0.245	-0.426	-6.524	-0.194	0.759	0.671	-2.354	-4.672	-0.628	0.029	0.019
LBR	6.991	12.178	0.790	0.000	0.000	2.492	-3.632	0.581	0.038	0.015	8.133	23.236	0.908	0.000	0.000
LCAT	-10.215	26.153	-1.910	0.000	0.000	-6.562	10.833	-2.909	0.000	0.000	-12.560	59.360	-2.465	0.000	0.000
LCK	0.545	-6.557	0.019	0.717	0.588	1.202	-5.903	0.385	0.340	0.232	1.154	-6.758	0.136	0.306	0.249
LDLR	-3.577	-0.797	-0.648	0.004	0.001	-3.419	-1.157	-0.577	0.005	0.001	-6.046	10.075	-0.779	0.000	0.000
LDLRAP1	0.396	-6.627	0.028	0.798	0.693	-0.568	-6.455	-0.089	0.675	0.572	2.850	-3.403	0.241	0.008	0.005
LEP	-5.012	4.148	-0.723	0.000	0.000	-1.875	-4.902	-0.148	0.123	0.064	0.847	-7.064	0.034	0.455	0.398
LGALS2	-0.626	-6.510	-0.064	0.673	0.533	-0.831	-6.273	-0.185	0.524	0.408	1.561	-6.208	0.279	0.156	0.119
LGALS3	4.551	2.451	0.522	0.000	0.000	3.549	-0.764	1.779	0.003	0.001	4.696	3.309	1.074	0.000	0.000
LGMN	0.627	-6.509	0.107	0.672	0.532	-0.349	-6.554	-0.064	0.805	0.728	0.424	-7.332	0.044	0.719	0.672
LIMA1	4.370	1.809	0.466	0.000	0.000	3.050	-2.220	0.775	0.011	0.003	4.327	1.725	0.469	0.000	0.000
LINC00339	2.263	-4.232	0.220	0.083	0.026	0.298	-6.570	0.058	0.834	0.766	6.236	11.143	0.118	0.000	0.000

LIPA	3.019	-2.405	0.490	0.017	0.003	2.564	-3.465	0.491	0.033	0.012	0.939	-6.982	0.102	0.406	0.348
LIPC	-5.732	6.952	-1.132	0.000	0.000	-2.131	-4.416	-0.959	0.078	0.036	-4.159	1.041	-0.782	0.000	0.000
LIPG	-5.056	4.313	-0.640	0.000	0.000	-3.107	-2.062	-1.252	0.010	0.003	-4.151	1.009	-0.557	0.000	0.000
LMNA	5.650	6.625	0.637	0.000	0.000	5.500	6.324	1.048	0.000	0.000	14.005	72.799	1.413	0.000	0.000
LPA	-6.196	8.839	-1.328	0.000	0.000	-6.495	10.539	-3.018	0.000	0.000	-12.156	55.715	-1.903	0.000	0.000
LPCAT3	0.863	-6.336	0.132	0.547	0.391	-0.354	-6.552	-0.031	0.802	0.724	-0.429	-7.330	-0.055	0.717	0.668
LPL	1.367	-5.783	0.088	0.317	0.175	4.529	2.555	1.390	0.000	0.000	6.911	15.159	0.668	0.000	0.000
LRP1	-1.477	-5.632	-0.167	0.276	0.144	-2.868	-2.706	-0.155	0.017	0.005	0.259	-7.388	0.028	0.835	0.796
LRP6	1.436	-5.689	0.054	0.291	0.155	-1.352	-5.716	-0.211	0.278	0.180	2.239	-4.933	0.210	0.039	0.026
LRP8	1.836	-5.058	0.110	0.167	0.070	1.685	-5.227	0.208	0.169	0.095	2.817	-3.497	0.076	0.009	0.005
LRPAP1	0.345	-6.646	0.034	0.825	0.731	3.392	-1.239	0.842	0.005	0.001	8.875	28.599	0.672	0.000	0.000
LSR	-5.876	7.534	-0.681	0.000	0.000	-0.952	-6.167	-0.380	0.460	0.344	-1.026	-6.897	-0.198	0.366	0.306
LSS	-2.656	-3.337	-0.174	0.038	0.010	-1.221	-5.881	-0.329	0.332	0.225	5.480	7.058	0.725	0.000	0.000
LYN	3.829	-0.008	0.488	0.002	0.000	2.285	-4.094	0.542	0.058	0.025	4.265	1.469	0.423	0.000	0.000
LZTFL1	-0.674	-6.479	-0.063	0.647	0.502	-3.474	-0.992	-0.299	0.004	0.001	-1.666	-6.040	-0.120	0.129	0.096
MAGEL2	-3.562	-0.844	-0.287	0.004	0.001	-2.810	-2.856	-0.209	0.019	0.006	1.398	-6.449	0.012	0.208	0.163
MAL	-1.073	-6.135	-0.048	0.444	0.287	-2.927	-2.553	-0.234	0.015	0.004	1.198	-6.707	0.080	0.287	0.232
MAL2	3.299	-1.625	0.619	0.008	0.001	0.895	-6.219	0.391	0.490	0.373	3.367	-1.837	0.676	0.002	0.001
MALL	3.800	-0.101	0.431	0.002	0.000	0.744	-6.341	0.258	0.573	0.459	3.605	-1.030	0.148	0.001	0.000
MAPK8IP1	0.324	-6.653	0.016	0.836	0.747	-4.833	3.690	-0.335	0.000	0.000	1.370	-6.486	0.137	0.217	0.171
MBTPS1	-2.215	-4.331	-0.203	0.090	0.030	2.093	-4.491	0.204	0.084	0.039	4.679	3.235	0.351	0.000	0.000
MBTPS2	1.004	-6.206	0.064	0.476	0.319	-1.645	-5.290	-0.089	0.180	0.103	3.321	-1.985	0.236	0.002	0.001
MED1	3.026	-2.386	0.246	0.017	0.003	4.949	4.135	0.646	0.000	0.000	6.659	13.618	0.489	0.000	0.000
MED15	2.224	-4.313	0.121	0.089	0.029	1.987	-4.696	0.184	0.101	0.050	9.694	34.887	0.785	0.000	0.000
MEF2A	3.649	-0.574	0.196	0.003	0.000	1.884	-4.885	0.310	0.121	0.063	1.007	-6.916	0.098	0.374	0.314
MEG3	-4.791	3.321	-0.896	0.000	0.000	-3.837	0.155	-0.301	0.002	0.000	1.755	-5.889	0.237	0.109	0.080
METTL21C	0.064	-6.703	0.002	0.970	0.949	-1.823	-4.993	-0.091	0.135	0.072	0.392	-7.345	0.010	0.742	0.695
MGAT1	-3.021	-2.400	-0.246	0.017	0.003	1.460	-5.568	0.154	0.238	0.148	6.918	15.203	0.443	0.000	0.000
MIR146A	-2.265	-4.228	-0.094	0.083	0.026	-4.386	2.038	-0.345	0.000	0.000	0.000	-7.422	0.000	1.000	1.000
MKKS	3.081	-2.238	0.396	0.015	0.003	3.223	-1.734	0.642	0.007	0.002	8.096	22.973	0.734	0.000	0.000
MKRN3	2.039	-4.685	0.077	0.121	0.045	-1.165	-5.946	-0.062	0.356	0.247	5.319	6.250	0.206	0.000	0.000
MKS1	2.579	-3.521	0.113	0.045	0.012	1.504	-5.506	0.121	0.224	0.136	8.844	28.372	0.356	0.000	0.000
MLC1	-1.202	-5.991	-0.075	0.385	0.233	-1.484	-5.534	-0.111	0.230	0.141	0.145	-7.411	0.003	0.915	0.885
MMEL1	-2.201	-4.360	-0.096	0.093	0.031	-1.001	-6.119	-0.091	0.435	0.319	2.451	-4.442	0.053	0.023	0.015
MOV10	0.297	-6.661	0.024	0.850	0.767	0.605	-6.433	0.097	0.654	0.547	10.739	43.403	0.836	0.000	0.000
MPO	-0.259	-6.672	-0.008	0.871	0.797	-2.409	-3.821	-0.167	0.045	0.018	-1.067	-6.854	-0.022	0.347	0.286
MSI1	-2.080	-4.605	-0.140	0.114	0.041	0.520	-6.480	0.037	0.703	0.604	5.522	7.276	1.027	0.000	0.000
MSMO1	-1.827	-5.075	-0.096	0.170	0.072	-2.280	-4.105	-0.547	0.059	0.025	-1.983	-5.468	-0.303	0.069	0.048
MSR1	1.661	-5.353	0.093	0.216	0.101	-1.028	-6.092	-0.334	0.421	0.307	-1.776	-5.852	-0.181	0.105	0.076
MSTN	-0.736	-6.436	-0.027	0.614	0.464	0.727	-6.353	0.041	0.582	0.469	2.204	-5.011	0.040	0.042	0.028
MTF1	4.180	1.153	0.376	0.001	0.000	2.930	-2.543	0.513	0.015	0.004	5.665	8.015	0.311	0.000	0.000
MTOR	1.974	-4.809	0.192	0.135	0.052	3.718	-0.232	0.528	0.002	0.000	6.383	11.989	0.585	0.000	0.000

MTTP	-5.471	5.914	-0.887	0.000	0.000	-1.869	-4.912	-1.081	0.124	0.065	-4.494	2.426	-0.942	0.000	0.000
MVD	1.449	-5.671	0.169	0.286	0.151	1.998	-4.676	0.306	0.099	0.049	9.065	30.026	1.242	0.000	0.000
MVK	-0.183	-6.688	-0.022	0.911	0.855	-2.042	-4.591	-0.226	0.092	0.044	0.966	-6.957	0.103	0.395	0.335
MYLIP	3.521	-0.968	0.485	0.005	0.001	-6.421	10.215	-0.335	0.000	0.000	4.927	4.359	0.527	0.000	0.000
MYO5B	1.029	-6.180	0.075	0.464	0.306	1.940	-4.783	0.478	0.110	0.056	2.575	-4.137	0.263	0.017	0.010
MYOD1	-1.978	-4.801	-0.073	0.134	0.051	-3.310	-1.481	-0.212	0.006	0.001	1.021	-6.902	0.011	0.368	0.308
NACC2	4.557	2.473	0.304	0.000	0.000	-2.280	-4.104	-0.184	0.059	0.025	8.462	25.569	0.667	0.000	0.000
NAXE	5.668	6.694	0.543	0.000	0.000	4.261	1.595	0.758	0.001	0.000	13.461	67.675	1.281	0.000	0.000
NCOA1	1.462	-5.654	0.066	0.282	0.148	3.268	-1.603	0.524	0.007	0.002	2.294	-4.810	0.171	0.034	0.022
NCOA2	3.556	-0.860	0.231	0.004	0.001	4.435	2.214	1.055	0.000	0.000	6.106	10.410	0.647	0.000	0.000
NCOA6	4.460	2.125	0.209	0.000	0.000	4.784	3.504	0.612	0.000	0.000	9.425	32.782	0.738	0.000	0.000
NCOR1	-7.191	13.036	-0.943	0.000	0.000	-10.105	27.120	-1.674	0.000	0.000	-5.173	5.531	-0.447	0.000	0.000
NCOR2	2.484	-3.744	0.189	0.055	0.015	-6.142	9.011	-0.397	0.000	0.000	9.736	35.218	0.900	0.000	0.000
NFIL3	-3.214	-1.867	-0.563	0.011	0.002	-4.170	1.277	-1.076	0.001	0.000	-6.290	11.454	-0.810	0.000	0.000
NFKBIA	-3.480	-1.090	-0.383	0.005	0.001	-4.070	0.932	-0.255	0.001	0.000	-0.526	-7.284	-0.056	0.651	0.599
NFYA	5.900	7.630	0.275	0.000	0.000	4.033	0.805	0.895	0.001	0.000	8.613	26.665	0.896	0.000	0.000
NFYB	-4.111	0.921	-0.231	0.001	0.000	2.468	-3.689	0.447	0.040	0.016	6.911	15.160	0.620	0.000	0.000
NFYC	0.739	-6.434	0.049	0.613	0.462	3.619	-0.546	0.505	0.003	0.000	10.088	38.041	0.679	0.000	0.000
NGLY1	0.622	-6.513	0.043	0.675	0.536	2.293	-4.077	0.296	0.057	0.024	5.039	4.889	0.258	0.000	0.000
NPAP1	-1.491	-5.612	-0.056	0.271	0.140	-2.265	-4.137	-0.196	0.061	0.026	-0.102	-7.417	-0.001	0.945	0.919
NPAS4	-1.822	-5.084	-0.082	0.171	0.072	-2.406	-3.829	-0.159	0.046	0.018	-2.490	-4.349	-0.007	0.021	0.013
NPC1	7.636	14.952	0.999	0.000	0.000	4.343	1.885	1.006	0.000	0.000	8.597	26.549	0.811	0.000	0.000
NPC1L1	-4.299	1.562	-1.071	0.000	0.000	-4.437	2.222	-0.478	0.000	0.000	-3.437	-1.605	-0.892	0.001	0.001
NPC2	4.078	0.812	0.442	0.001	0.000	4.063	0.910	0.744	0.001	0.000	8.260	24.130	0.869	0.000	0.000
NPHP1	2.271	-4.215	0.085	0.082	0.026	-3.059	-2.194	-0.185	0.011	0.003	3.008	-2.953	0.047	0.005	0.003
NR1H2	2.440	-3.844	0.223	0.059	0.017	3.129	-2.002	0.350	0.009	0.002	9.587	34.045	0.662	0.000	0.000
NR1H3	-5.016	4.162	-0.407	0.000	0.000	-1.044	-6.076	-0.147	0.414	0.299	3.526	-1.303	0.275	0.001	0.000
NR1H4	-2.481	-3.749	-0.304	0.055	0.015	-3.623	-0.534	-0.496	0.003	0.000	-1.441	-6.387	-0.158	0.193	0.150
NR1I2	-6.379	9.598	-1.080	0.000	0.000	-3.657	-0.424	-0.709	0.003	0.000	-6.029	9.983	-1.095	0.000	0.000
NR3C1	4.122	0.959	0.207	0.001	0.000	1.649	-5.283	0.253	0.179	0.102	0.647	-7.213	0.053	0.578	0.518
NSDHL	3.964	0.431	0.568	0.001	0.000	1.699	-5.203	0.359	0.166	0.093	8.122	23.159	0.839	0.000	0.000
NUP107	6.474	9.994	0.521	0.000	0.000	3.974	0.608	0.630	0.001	0.000	10.062	37.824	0.813	0.000	0.000
OCRL	3.115	-2.146	0.362	0.014	0.003	4.239	1.517	0.800	0.001	0.000	11.060	46.125	1.047	0.000	0.000
OSBP	-0.648	-6.496	-0.038	0.661	0.519	-2.163	-4.349	-0.283	0.074	0.033	1.066	-6.855	0.075	0.348	0.287
OSBP2	3.964	0.432	0.364	0.001	0.000	-5.147	4.906	-0.436	0.000	0.000	6.282	11.406	0.518	0.000	0.000
OSBPL10	1.629	-5.403	0.139	0.226	0.107	-5.052	4.534	-0.406	0.000	0.000	2.277	-4.849	0.123	0.035	0.023
OSBPL1A	-1.166	-6.032	-0.090	0.401	0.247	-3.098	-2.088	-0.257	0.010	0.003	-0.083	-7.419	-0.008	0.956	0.934
OSBPL2	-0.550	-6.555	-0.048	0.714	0.584	3.167	-1.894	0.363	0.009	0.002	9.192	30.987	0.471	0.000	0.000
OSBPL3	4.995	4.081	0.207	0.000	0.000	4.339	1.868	1.237	0.000	0.000	8.591	26.510	0.748	0.000	0.000
OSBPL5	-0.719	-6.448	-0.059	0.623	0.474	-2.756	-2.994	-0.250	0.022	0.007	2.849	-3.407	0.216	0.008	0.005
OSBPL6	-1.199	-5.994	-0.037	0.386	0.234	-5.282	5.443	-1.161	0.000	0.000	-0.226	-7.397	-0.013	0.856	0.822
OSBPL7	1.256	-5.926	0.072	0.363	0.213	-4.418	2.154	-0.300	0.000	0.000	5.294	6.122	0.375	0.000	0.000

OSBPL8	3.464	-1.139	0.348	0.005	0.001	-2.299	-4.064	-0.163	0.057	0.024	1.271	-6.617	0.113	0.256	0.204
OSBPL9	-1.195	-6.000	-0.036	0.388	0.236	-1.381	-5.677	-0.263	0.267	0.171	1.550	-6.226	0.162	0.159	0.122
PAF1	-2.919	-2.671	-0.253	0.021	0.005	2.075	-4.526	0.186	0.087	0.041	7.970	22.102	0.551	0.000	0.000
PAG1	3.918	0.281	0.363	0.002	0.000	1.398	-5.655	0.501	0.261	0.166	3.598	-1.056	0.401	0.001	0.000
PBX1	-5.197	4.852	-0.321	0.000	0.000	-1.396	-5.658	-0.205	0.262	0.166	0.014	-7.422	0.001	1.000	0.989
PCK1	-8.014	16.594	-1.527	0.000	0.000	-3.332	-1.418	-1.984	0.006	0.001	-8.608	26.633	-2.936	0.000	0.000
PCK2	-5.327	5.353	-0.929	0.000	0.000	-4.958	4.171	-1.508	0.000	0.000	-5.903	9.290	-1.100	0.000	0.000
PCSK9	3.190	-1.935	0.419	0.011	0.002	1.411	-5.636	0.387	0.256	0.162	5.603	7.693	0.957	0.000	0.000
PCYOX1	-6.872	11.673	-0.741	0.000	0.000	-1.834	-4.974	-0.301	0.132	0.070	-0.763	-7.132	-0.081	0.506	0.446
PCYT2	-0.978	-6.231	-0.110	0.489	0.331	-0.968	-6.152	-0.145	0.451	0.336	5.578	7.563	0.628	0.000	0.000
PDIA2	0.934	-6.272	0.050	0.511	0.353	-3.429	-1.129	-0.239	0.005	0.001	3.409	-1.697	0.490	0.001	0.001
PDK3	-2.236	-4.289	-0.245	0.087	0.028	-1.506	-5.502	-0.350	0.223	0.136	1.418	-6.420	0.101	0.201	0.157
PEX12	-0.886	-6.316	-0.023	0.535	0.379	-0.915	-6.201	-0.159	0.479	0.363	3.159	-2.497	0.239	0.003	0.002
PHKA2	-3.012	-2.424	-0.296	0.017	0.003	1.624	-5.324	0.256	0.186	0.108	3.096	-2.688	0.287	0.004	0.002
PHKB	4.140	1.020	0.276	0.001	0.000	2.426	-3.784	0.555	0.044	0.017	4.568	2.747	0.349	0.000	0.000
PHKG2	0.889	-6.313	0.072	0.533	0.377	-2.444	-3.742	-0.140	0.042	0.016	7.378	18.125	0.519	0.000	0.000
PI4K2A	5.904	7.647	0.315	0.000	0.000	3.496	-0.924	0.518	0.004	0.001	8.709	27.370	0.609	0.000	0.000
PIK3R5	0.914	-6.291	0.031	0.521	0.364	-1.969	-4.730	-0.122	0.105	0.052	-1.005	-6.918	-0.062	0.375	0.315
PIKFYVE	-1.708	-5.276	-0.082	0.202	0.092	-3.423	-1.146	-0.199	0.005	0.001	2.702	-3.808	0.200	0.012	0.007
PLA2G2A	0.244	-6.675	0.101	0.879	0.808	-0.439	-6.519	-0.415	0.752	0.662	-2.356	-4.667	-1.209	0.029	0.019
PLA2G2E	-1.853	-5.029	-0.056	0.163	0.068	-3.640	-0.478	-0.221	0.003	0.000	0.886	-7.031	0.029	0.435	0.376
PLA2G3	-1.876	-4.988	-0.076	0.158	0.064	-4.560	2.671	-0.398	0.000	0.000	1.690	-6.001	0.014	0.124	0.092
PLA2G5	-2.533	-3.631	-0.112	0.050	0.013	-3.823	0.107	-0.310	0.002	0.000	-2.673	-3.884	-0.198	0.013	0.008
PLA2G7	3.428	-1.246	0.413	0.006	0.001	2.093	-4.490	0.842	0.084	0.039	3.289	-2.090	0.524	0.002	0.001
PLAUR	1.472	-5.638	0.163	0.277	0.145	-3.520	-0.852	-0.537	0.004	0.001	-0.830	-7.078	-0.104	0.464	0.407
PLPP6	-1.268	-5.912	-0.111	0.358	0.209	0.534	-6.473	0.115	0.695	0.595	-1.491	-6.314	-0.131	0.177	0.137
PLSCR1	0.161	-6.692	0.011	0.922	0.872	-4.868	3.824	-0.277	0.000	0.000	-2.053	-5.329	-0.285	0.059	0.041
PLTP	1.590	-5.464	0.130	0.238	0.116	0.033	-6.614	0.014	0.983	0.974	-1.751	-5.896	-0.362	0.110	0.081
PMM2	-1.591	-5.463	-0.143	0.238	0.116	0.341	-6.557	0.059	0.810	0.734	3.499	-1.396	0.188	0.001	0.001
PMP2	-3.701	-0.412	-0.120	0.003	0.000	-5.730	7.272	-0.282	0.000	0.000	-1.379	-6.474	-0.022	0.214	0.168
PMVK	1.557	-5.515	0.143	0.249	0.124	1.765	-5.094	0.269	0.149	0.081	11.241	47.671	1.130	0.000	0.000
PNKP	0.123	-6.698	0.009	0.941	0.903	4.052	0.871	0.587	0.001	0.000	10.396	40.555	0.815	0.000	0.000
PNLIP	-1.195	-6.000	-0.036	0.388	0.236	-2.866	-2.712	-0.178	0.017	0.005	1.126	-6.789	0.045	0.318	0.261
PNRC1	-2.296	-4.160	-0.191	0.078	0.024	-2.913	-2.588	-0.426	0.015	0.005	-5.257	5.942	-0.593	0.000	0.000
PON1	-5.198	4.854	-1.232	0.000	0.000	-4.234	1.500	-1.830	0.001	0.000	-5.423	6.770	-1.560	0.000	0.000
POU2AF1	-1.016	-6.194	-0.054	0.470	0.313	1.107	-6.009	0.499	0.383	0.271	0.718	-7.164	0.057	0.534	0.473
PPA2	-5.337	5.391	-0.820	0.000	0.000	-3.716	-0.236	-0.399	0.002	0.000	2.665	-3.904	0.210	0.013	0.008
PPARA	-1.044	-6.165	-0.034	0.457	0.300	-2.448	-3.733	-0.162	0.042	0.016	0.319	-7.371	0.035	0.793	0.750
PPARD	-2.166	-4.433	-0.110	0.098	0.033	4.389	2.049	0.706	0.000	0.000	7.465	18.694	0.856	0.000	0.000
PPARG	4.232	1.332	0.553	0.001	0.000	1.246	-5.850	0.311	0.321	0.216	5.828	8.883	0.704	0.000	0.000
PPARGC1A	-6.561	10.359	-1.442	0.000	0.000	-6.178	9.163	-0.567	0.000	0.000	-4.145	0.986	-0.743	0.000	0.000
PPP1R17	-0.935	-6.272	-0.031	0.511	0.353	-4.074	0.948	-0.286	0.001	0.000	-1.158	-6.753	-0.003	0.304	0.247

PPT1	4.623	2.711	0.680	0.000	0.000	3.596	-0.616	0.739	0.003	0.001	6.426	12.238	0.780	0.000	0.000
PRKAA1	-1.458	-5.659	-0.114	0.283	0.149	1.472	-5.551	0.327	0.234	0.144	0.193	-7.403	0.017	0.880	0.847
PRKAA2	0.985	-6.224	0.045	0.485	0.328	4.347	1.897	2.613	0.000	0.000	6.273	11.357	0.771	0.000	0.000
PRLR	3.453	-1.171	0.207	0.006	0.001	1.966	-4.735	0.542	0.105	0.052	2.333	-4.722	0.248	0.031	0.020
PRNP	-2.698	-3.235	-0.422	0.035	0.009	-1.868	-4.915	-0.699	0.125	0.065	-3.525	-1.307	-0.592	0.001	0.000
PROM1	-5.159	4.704	-0.711	0.000	0.000	-3.656	-0.428	-1.148	0.003	0.000	-1.338	-6.530	-0.169	0.229	0.182
PROM2	-3.748	-0.264	-0.186	0.002	0.000	-4.935	4.082	-0.377	0.000	0.000	0.095	-7.418	0.010	0.948	0.925
PSAP	0.774	-6.408	0.061	0.594	0.441	2.165	-4.345	0.245	0.073	0.033	5.228	5.797	0.447	0.000	0.000
PSMB8	2.101	-4.564	0.258	0.110	0.039	1.407	-5.642	0.438	0.257	0.163	4.205	1.225	0.590	0.000	0.000
PTCH1	0.195	-6.686	0.008	0.905	0.846	-4.460	2.305	-0.366	0.000	0.000	1.104	-6.814	0.054	0.328	0.270
PYGL	-0.663	-6.486	-0.096	0.653	0.509	-0.705	-6.368	-0.196	0.595	0.483	-0.750	-7.141	-0.097	0.513	0.454
RAI1	-1.276	-5.901	-0.057	0.354	0.206	-3.440	-1.094	-0.242	0.004	0.001	4.874	4.115	0.433	0.000	0.000
RAN	7.374	13.822	0.782	0.000	0.000	4.662	3.046	0.898	0.000	0.000	10.696	43.046	1.041	0.000	0.000
RDH8	-1.505	-5.591	-0.069	0.266	0.136	-3.097	-2.090	-0.209	0.010	0.003	2.339	-4.708	0.132	0.030	0.020
REST	0.385	-6.631	0.017	0.804	0.701	-1.989	-4.692	-0.154	0.101	0.050	3.164	-2.482	0.213	0.003	0.002
RORC	-2.390	-3.956	-0.321	0.066	0.019	-1.946	-4.772	-0.684	0.109	0.055	0.436	-7.327	0.079	0.713	0.663
RPL14	0.589	-6.532	0.124	0.693	0.557	2.140	-4.397	0.274	0.077	0.035	8.511	25.924	0.933	0.000	0.000
RPS6KB1	-0.094	-6.701	-0.006	0.955	0.925	3.794	0.013	0.485	0.002	0.000	6.977	15.566	0.445	0.000	0.000
RSPO1	-0.922	-6.283	-0.034	0.517	0.359	-1.878	-4.896	-0.120	0.123	0.064	0.581	-7.253	0.009	0.614	0.561
RXRA	-3.396	-1.341	-0.378	0.007	0.001	-5.062	4.572	-0.943	0.000	0.000	2.789	-3.574	0.277	0.009	0.006
RXRB	1.508	-5.586	0.122	0.265	0.135	3.877	0.286	0.792	0.001	0.000	12.147	55.631	0.965	0.000	0.000
S100A11	2.772	-3.050	0.364	0.030	0.007	1.405	-5.645	0.558	0.258	0.164	4.242	1.376	0.932	0.000	0.000
SAMD1	2.062	-4.639	0.111	0.117	0.043	3.863	0.238	0.459	0.001	0.000	13.558	68.584	1.337	0.000	0.000
SAR1B	2.491	-3.726	0.332	0.054	0.015	-0.523	-6.479	-0.105	0.702	0.602	0.157	-7.410	0.016	0.906	0.875
SC5D	-3.354	-1.466	-0.543	0.007	0.001	-3.898	0.356	-1.087	0.001	0.000	-3.866	-0.088	-0.535	0.000	0.000
SCAP	-0.242	-6.676	-0.019	0.880	0.809	1.969	-4.730	0.282	0.105	0.052	10.124	38.327	0.732	0.000	0.000
SCAPER	-0.396	-6.627	-0.025	0.798	0.693	-1.170	-5.940	-0.220	0.354	0.245	2.661	-3.915	0.133	0.013	0.008
SCARB1	1.338	-5.822	0.149	0.329	0.185	-4.748	3.367	-0.224	0.000	0.000	4.148	0.999	0.486	0.000	0.000
SCARB2	-1.662	-5.351	-0.105	0.215	0.100	1.889	-4.877	0.371	0.120	0.062	2.372	-4.631	0.191	0.028	0.018
SCARF1	0.358	-6.641	0.012	0.818	0.721	-1.379	-5.679	-0.098	0.268	0.171	3.048	-2.833	0.203	0.004	0.002
SCD	0.276	-6.667	0.058	0.862	0.783	3.629	-0.513	1.226	0.003	0.000	1.350	-6.514	0.358	0.225	0.178
SCNN1A	-0.341	-6.647	-0.049	0.827	0.734	-4.970	4.214	-0.360	0.000	0.000	0.230	-7.396	0.031	0.854	0.818
SCNN1B	1.043	-6.166	0.040	0.457	0.300	-3.592	-0.628	-0.283	0.003	0.001	1.705	-5.976	0.095	0.120	0.089
SCNN1G	0.197	-6.686	0.007	0.904	0.844	-3.160	-1.914	-0.157	0.009	0.002	0.673	-7.196	0.032	0.560	0.501
SCP2	-2.232	-4.295	-0.535	0.088	0.028	-3.341	-1.390	-0.569	0.006	0.001	-6.845	14.752	-1.053	0.000	0.000
SCP2D1	-0.720	-6.447	-0.025	0.622	0.474	-3.807	0.057	-0.261	0.002	0.000	0.000	-7.422	0.000	1.000	1.000
SCRIB	5.492	5.999	0.619	0.000	0.000	4.805	3.585	1.042	0.000	0.000	13.843	71.265	1.599	0.000	0.000
SDCCAG8	3.088	-2.218	0.133	0.014	0.003	2.521	-3.566	0.289	0.036	0.014	7.878	21.469	0.518	0.000	0.000
SDR42E1	-3.076	-2.251	-0.126	0.015	0.003	-2.752	-3.005	-0.387	0.022	0.007	-0.849	-7.063	-0.041	0.454	0.397
SEC14L2	-2.872	-2.795	-0.603	0.024	0.005	-5.418	5.988	-0.443	0.000	0.000	-3.333	-1.948	-0.755	0.002	0.001
SEC23A	1.852	-5.031	0.130	0.163	0.068	-1.345	-5.725	-0.212	0.281	0.182	-1.150	-6.763	-0.108	0.308	0.251
SEC24A	-1.257	-5.925	-0.061	0.362	0.213	3.267	-1.606	0.650	0.007	0.002	0.627	-7.225	0.057	0.589	0.531

SEC24B	-8.034	16.681	-0.429	0.000	0.000	-4.374	1.996	-0.777	0.000	0.000	-4.246	1.390	-0.439	0.000	0.000
SEC24C	1.492	-5.611	0.062	0.271	0.140	6.277	9.589	0.731	0.000	0.000	3.030	-2.888	0.436	0.005	0.003
SEC24D	-0.551	-6.554	-0.041	0.714	0.583	-1.858	-4.933	-0.383	0.127	0.066	-1.595	-6.155	-0.165	0.147	0.111
SEMA3B	1.172	-6.026	0.068	0.398	0.245	-0.022	-6.614	-0.001	0.989	0.983	4.399	2.023	0.711	0.000	0.000
SERINC1	-2.671	-3.301	-0.228	0.037	0.009	-3.390	-1.245	-0.494	0.005	0.001	-3.321	-1.984	-0.335	0.002	0.001
SERPINA12	0.497	-6.582	0.114	0.744	0.621	-1.063	-6.057	-0.129	0.404	0.291	2.452	-4.442	0.365	0.023	0.015
SERPINF1	-3.080	-2.241	-0.492	0.015	0.003	-0.624	-6.421	-0.127	0.642	0.534	-1.661	-6.049	-0.276	0.130	0.097
SETX	2.255	-4.247	0.145	0.084	0.027	-2.444	-3.743	-0.281	0.042	0.017	1.973	-5.486	0.143	0.070	0.049
SGK1	-0.942	-6.265	-0.221	0.507	0.349	-1.923	-4.814	-0.737	0.113	0.058	-3.642	-0.902	-0.575	0.001	0.000
SHH	-1.723	-5.252	-0.073	0.198	0.089	-4.157	1.230	-0.268	0.001	0.000	-0.698	-7.179	-0.081	0.546	0.485
SIDT1	-0.116	-6.698	-0.004	0.944	0.908	0.530	-6.475	0.085	0.697	0.598	1.241	-6.654	0.048	0.268	0.215
SIRT1	-1.019	-6.190	-0.115	0.469	0.311	-2.082	-4.512	-0.384	0.085	0.040	-1.479	-6.332	-0.131	0.180	0.140
SIRT6	-1.129	-6.075	-0.038	0.418	0.262	-2.635	-3.295	-0.155	0.028	0.010	11.401	49.055	0.943	0.000	0.000
SLC22A24	-1.941	-4.870	-0.085	0.142	0.056	-1.377	-5.682	-0.102	0.269	0.172	2.053	-5.328	0.018	0.059	0.041
SLC25A13	-2.809	-2.957	-0.393	0.028	0.006	-1.842	-4.961	-0.397	0.130	0.069	-0.958	-6.964	-0.104	0.398	0.339
SLC25A36	-0.738	-6.434	-0.034	0.613	0.462	-1.649	-5.284	-0.097	0.179	0.103	1.898	-5.631	0.148	0.082	0.058
SLC27A2	-3.634	-0.621	-0.691	0.003	0.000	-2.500	-3.615	-1.222	0.038	0.014	-5.996	9.798	-1.278	0.000	0.000
SLC27A4	1.049	-6.160	0.049	0.454	0.297	-0.518	-6.481	-0.071	0.705	0.606	3.656	-0.850	0.336	0.001	0.000
SLC27A5	-5.726	6.930	-1.458	0.000	0.000	-5.801	7.568	-0.422	0.000	0.000	-6.898	15.078	-1.782	0.000	0.000
SLC2A3	-1.455	-5.662	-0.386	0.284	0.150	-2.366	-3.919	-0.584	0.050	0.020	-1.955	-5.522	-0.254	0.073	0.051
SLC37A4	-4.424	1.999	-0.630	0.000	0.000	-2.166	-4.344	-0.567	0.073	0.033	-2.020	-5.394	-0.229	0.064	0.044
SLC38A9	4.981	4.029	0.403	0.000	0.000	4.114	1.081	0.587	0.001	0.000	8.911	28.868	0.515	0.000	0.000
SMARCD3	1.056	-6.152	0.072	0.451	0.294	-3.944	0.508	-0.338	0.001	0.000	5.081	5.089	0.600	0.000	0.000
SMO	2.717	-3.188	0.326	0.034	0.008	2.016	-4.642	0.300	0.096	0.047	7.295	17.588	1.097	0.000	0.000
SMPD1	-4.612	2.669	-0.449	0.000	0.000	-3.117	-2.033	-0.245	0.010	0.002	-1.262	-6.628	-0.111	0.259	0.208
SMYD3	6.464	9.955	0.770	0.000	0.000	3.435	-1.111	1.127	0.004	0.001	10.387	40.477	1.087	0.000	0.000
SOAT1	4.727	3.088	0.245	0.000	0.000	3.238	-1.690	0.657	0.007	0.002	5.214	5.731	0.499	0.000	0.000
SOAT2	3.516	-0.982	0.339	0.005	0.001	-0.647	-6.407	-0.197	0.628	0.519	3.276	-2.130	0.744	0.002	0.001
SOD1	-2.242	-4.276	-0.213	0.086	0.028	-2.242	-4.187	-0.421	0.063	0.027	-2.014	-5.406	-0.235	0.064	0.045
SORBS1	-5.727	6.933	-0.572	0.000	0.000	-3.164	-1.902	-0.194	0.009	0.002	-2.611	-4.046	-0.253	0.015	0.009
SORL1	-8.690	19.538	-1.233	0.000	0.000	-5.930	8.108	-1.629	0.000	0.000	-6.411	12.153	-0.837	0.000	0.000
SORT1	4.777	3.272	0.707	0.000	0.000	3.493	-0.936	0.844	0.004	0.001	10.168	38.687	1.383	0.000	0.000
SP1	0.702	-6.460	0.041	0.632	0.485	3.934	0.475	0.675	0.001	0.000	7.135	16.565	0.553	0.000	0.000
SPIB	-1.136	-6.067	-0.037	0.414	0.259	-1.736	-5.143	-0.264	0.156	0.086	1.413	-6.427	0.147	0.202	0.158
SQLE	7.231	13.207	1.930	0.000	0.000	4.413	2.134	2.077	0.000	0.000	10.429	40.824	2.015	0.000	0.000
SRD5A1	-4.425	2.002	-0.602	0.000	0.000	-5.098	4.715	-1.636	0.000	0.000	-6.723	14.006	-0.877	0.000	0.000
SRD5A2	-8.153	17.196	-1.555	0.000	0.000	-5.282	5.443	-2.318	0.000	0.000	-9.451	32.986	-1.934	0.000	0.000
SRD5A3	0.992	-6.217	0.039	0.482	0.324	3.607	-0.583	0.936	0.003	0.001	7.738	20.516	0.547	0.000	0.000
SREBF1	-1.714	-5.267	-0.222	0.200	0.091	-1.214	-5.888	-0.167	0.334	0.228	-0.260	-7.388	-0.039	0.835	0.795
SREBF2	-0.115	-6.699	-0.004	0.945	0.909	3.754	-0.115	0.768	0.002	0.000	9.177	30.873	1.063	0.000	0.000
SSTR2	-3.159	-2.023	-0.376	0.012	0.002	-1.724	-5.163	-0.312	0.159	0.088	0.240	-7.393	0.026	0.847	0.811
SSTR3	-1.945	-4.863	-0.049	0.142	0.055	-3.763	-0.085	-0.222	0.002	0.000	2.194	-5.033	0.080	0.043	0.029

SSTR4	-3.490	-1.062	-0.117	0.005	0.001	-3.849	0.194	-0.333	0.002	0.000	0.102	-7.417	0.000	0.945	0.919
SSTR5	-0.142	-6.695	-0.005	0.931	0.887	-0.983	-6.137	-0.072	0.444	0.328	3.231	-2.274	0.415	0.002	0.001
STAB1	-4.966	3.975	-0.582	0.000	0.000	-5.946	8.174	-0.348	0.000	0.000	-4.388	1.978	-0.520	0.000	0.000
STAB2	-12.550	35.921	-1.557	0.000	0.000	-11.680	34.355	-2.179	0.000	0.000	-31.039	241.062	-1.391	0.000	0.000
STAR	0.817	-6.373	0.033	0.571	0.416	-3.857	0.219	-0.263	0.002	0.000	1.061	-6.861	0.046	0.350	0.290
STARD3	3.327	-1.545	0.225	0.008	0.001	2.999	-2.359	0.332	0.013	0.003	11.786	52.421	0.879	0.000	0.000
STARD3NL	6.634	10.668	0.494	0.000	0.000	1.982	-4.705	0.514	0.102	0.051	5.929	9.434	0.538	0.000	0.000
STARD4	0.728	-6.442	0.022	0.619	0.469	-1.308	-5.773	-0.333	0.295	0.194	0.474	-7.310	0.052	0.688	0.636
STARD5	-8.183	17.329	-1.110	0.000	0.000	-5.211	5.159	-1.070	0.000	0.000	-4.703	3.338	-0.161	0.000	0.000
STC1	4.613	2.672	0.644	0.000	0.000	4.813	3.613	1.631	0.000	0.000	6.923	15.230	0.952	0.000	0.000
STOM	-4.696	2.975	-0.995	0.000	0.000	-2.366	-3.917	-0.185	0.050	0.020	-4.291	1.575	-0.557	0.000	0.000
STS	0.923	-6.283	0.052	0.516	0.359	-2.221	-4.230	-0.591	0.066	0.029	-0.134	-7.413	-0.016	0.923	0.893
STX12	-0.481	-6.590	-0.029	0.753	0.632	-0.614	-6.428	-0.075	0.648	0.541	3.838	-0.191	0.259	0.000	0.000
STX5	3.081	-2.239	0.321	0.015	0.003	2.016	-4.640	0.211	0.096	0.047	9.280	31.662	0.569	0.000	0.000
SULT1E1	-3.506	-1.013	-0.238	0.005	0.001	-3.975	0.611	-2.071	0.001	0.000	-4.050	0.611	-0.605	0.000	0.000
SULT2A1	-4.087	0.839	-0.978	0.001	0.000	-1.753	-5.114	-0.916	0.151	0.083	-4.483	2.379	-1.447	0.000	0.000
SULT2B1	-1.900	-4.945	-0.064	0.152	0.061	-2.188	-4.299	-0.170	0.070	0.031	1.910	-5.607	0.151	0.080	0.057
SYNE1	-2.317	-4.116	-0.162	0.075	0.023	-6.876	12.222	-0.489	0.000	0.000	-5.953	9.564	-0.258	0.000	0.000
SYNE2	-1.877	-4.987	-0.069	0.158	0.064	-5.466	6.184	-0.344	0.000	0.000	0.855	-7.058	0.072	0.452	0.393
SYP	1.464	-5.650	0.069	0.280	0.147	-2.140	-4.397	-0.175	0.077	0.035	5.461	6.963	0.358	0.000	0.000
TBL1X	-2.360	-4.021	-0.237	0.069	0.021	-3.412	-1.181	-0.237	0.005	0.001	3.857	-0.124	0.341	0.000	0.000
TBL1XR1	6.313	9.325	0.547	0.000	0.000	5.781	7.483	0.851	0.000	0.000	7.128	16.519	0.565	0.000	0.000
TBX2	2.920	-2.670	0.270	0.021	0.005	2.211	-4.251	0.420	0.067	0.030	6.942	15.348	0.699	0.000	0.000
TDP1	-5.295	5.230	-0.714	0.000	0.000	2.848	-2.758	0.411	0.018	0.005	8.713	27.397	0.510	0.000	0.000
TFAP4	0.722	-6.446	0.027	0.621	0.472	-1.879	-4.894	-0.165	0.122	0.063	9.651	34.552	0.543	0.000	0.000
TGFB1	-0.075	-6.702	-0.002	0.964	0.940	-2.344	-3.967	-0.196	0.052	0.021	3.332	-1.949	0.617	0.002	0.001
TGS1	5.399	5.635	0.305	0.000	0.000	4.761	3.418	0.914	0.000	0.000	7.968	22.083	0.623	0.000	0.000
THBS1	-3.379	-1.390	-0.906	0.007	0.001	-5.539	6.481	-2.064	0.000	0.000	-6.137	10.584	-1.265	0.000	0.000
THEM5	-2.322	-4.105	-0.067	0.075	0.023	-0.939	-6.179	-0.049	0.466	0.350	10.852	44.360	1.207	0.000	0.000
TIMM10	3.794	-0.120	0.465	0.002	0.000	0.726	-6.353	0.108	0.582	0.469	8.139	23.276	0.760	0.000	0.000
TM7SF2	-0.623	-6.512	-0.110	0.674	0.535	0.337	-6.558	0.117	0.811	0.737	3.746	-0.529	0.690	0.000	0.000
TMEM176B	-5.341	5.409	-0.386	0.000	0.000	-1.147	-5.966	-0.113	0.364	0.254	-1.330	-6.541	-0.216	0.232	0.184
TMEM241	5.394	5.614	0.395	0.000	0.000	1.053	-6.067	0.139	0.409	0.295	7.149	16.650	0.386	0.000	0.000
TMEM43	3.268	-1.714	0.198	0.009	0.002	-1.822	-4.996	-0.139	0.135	0.072	6.217	11.036	0.598	0.000	0.000
TMEM97	2.742	-3.124	0.400	0.032	0.008	1.309	-5.771	0.302	0.295	0.194	4.340	1.778	0.671	0.000	0.000
TNFRSF12A	2.701	-3.227	0.537	0.035	0.008	1.289	-5.797	0.296	0.303	0.201	3.917	0.102	0.747	0.000	0.000
TNFSF15	-5.402	5.644	-0.581	0.000	0.000	2.081	-4.514	0.437	0.086	0.040	4.143	0.978	0.276	0.000	0.000
TNPO3	-0.588	-6.533	-0.043	0.693	0.558	3.448	-1.071	0.398	0.004	0.001	8.271	24.207	0.646	0.000	0.000
TNRC6A	-6.086	8.386	-0.434	0.000	0.000	1.699	-5.203	0.268	0.166	0.093	3.393	-1.752	0.246	0.001	0.001
TNRC6B	1.514	-5.578	0.053	0.263	0.134	4.522	2.530	0.755	0.000	0.000	4.860	4.051	0.246	0.000	0.000
TNRC6B	-1.512	-5.580	-0.107	0.263	0.134	4.522	2.530	0.755	0.000	0.000	4.860	4.051	0.246	0.000	0.000
TNRC6C	-3.544	-0.899	-0.154	0.004	0.001	-3.241	-1.683	-0.221	0.007	0.002	3.697	-0.705	0.246	0.001	0.000

TP53INP1	1.411	-5.724	0.209	0.300	0.162	-1.282	-5.805	-0.210	0.306	0.203	2.179	-5.065	0.336	0.045	0.030
TREM2	-0.510	-6.576	-0.036	0.736	0.611	1.042	-6.078	0.162	0.414	0.300	6.974	15.549	1.246	0.000	0.000
TRIB3	1.879	-4.982	0.351	0.157	0.064	1.093	-6.025	0.306	0.390	0.277	3.633	-0.934	0.610	0.001	0.000
TRIM32	7.048	12.423	0.513	0.000	0.000	5.254	5.329	0.744	0.000	0.000	7.790	20.866	0.599	0.000	0.000
TSHB	-0.102	-6.700	-0.004	0.951	0.919	-1.711	-5.183	-0.123	0.162	0.090	0.256	-7.389	0.001	0.837	0.798
TSPO	1.724	-5.250	0.170	0.197	0.089	3.059	-2.195	0.709	0.011	0.003	4.107	0.834	0.712	0.000	0.000
TSPO2	-1.995	-4.770	-0.127	0.131	0.050	-3.481	-0.971	-0.419	0.004	0.001	6.359	11.850	0.545	0.000	0.000
TTC39B	-2.632	-3.396	-0.135	0.040	0.010	-4.219	1.448	-0.563	0.001	0.000	-4.768	3.633	-0.279	0.000	0.000
TTC8	2.224	-4.314	0.163	0.089	0.029	2.701	-3.133	0.713	0.025	0.008	2.724	-3.749	0.185	0.011	0.007
TTPA	-2.602	-3.467	-0.088	0.043	0.011	0.050	-6.613	0.020	0.974	0.960	-4.649	3.102	-1.006	0.000	0.000
UBA52	5.717	6.893	0.319	0.000	0.000	3.682	-0.346	0.336	0.002	0.000	8.652	26.952	0.852	0.000	0.000
UBE3B	4.186	1.175	0.323	0.001	0.000	5.488	6.273	0.610	0.000	0.000	8.983	29.404	0.542	0.000	0.000
UBR1	2.843	-2.870	0.151	0.026	0.006	1.814	-5.010	0.243	0.137	0.073	2.425	-4.505	0.149	0.025	0.016
UGT1A1	-2.441	-3.841	-0.552	0.059	0.017	-0.694	-6.376	-0.049	0.601	0.489	-5.270	6.003	-1.303	0.000	0.000
UGT1A6	3.969	0.450	0.934	0.001	0.000	2.040	-4.595	1.278	0.092	0.044	3.169	-2.467	0.521	0.003	0.002
UGT2A3	1.148	-6.053	0.186	0.409	0.255	-1.839	-4.966	-1.050	0.131	0.069	-1.640	-6.083	-0.337	0.136	0.102
UGT2B15	-1.220	-5.969	-0.350	0.377	0.226	-1.854	-4.940	-1.072	0.128	0.067	-3.119	-2.619	-1.015	0.003	0.002
UGT2B17	-2.523	-3.653	-0.476	0.051	0.014	-2.383	-3.880	-2.207	0.048	0.019	-2.449	-4.447	-0.706	0.023	0.015
UGT2B28	-2.517	-3.667	-0.607	0.051	0.014	-2.436	-3.761	-0.977	0.043	0.017	2.031	-5.373	0.098	0.062	0.043
UGT2B4	-0.923	-6.282	-0.157	0.516	0.359	-0.430	-6.523	-0.212	0.756	0.668	-0.638	-7.219	-0.168	0.583	0.524
UGT3A1	-1.561	-5.508	-0.327	0.247	0.122	-2.939	-2.520	-2.233	0.015	0.004	-2.945	-3.135	-0.681	0.006	0.003
USP8	1.435	-5.692	0.128	0.291	0.155	3.933	0.472	0.613	0.001	0.000	4.682	3.247	0.201	0.000	0.000
UTS2	-2.862	-2.820	-0.108	0.025	0.005	-1.457	-5.572	-0.103	0.240	0.148	2.603	-4.067	0.157	0.016	0.010
VAPA	1.190	-6.004	0.074	0.390	0.237	3.038	-2.252	0.423	0.012	0.003	7.469	18.723	0.502	0.000	0.000
VDAC1	5.295	5.228	0.399	0.000	0.000	3.048	-2.224	0.477	0.011	0.003	9.739	35.246	0.788	0.000	0.000
VDAC2	-1.423	-5.708	-0.057	0.296	0.159	2.390	-3.866	0.305	0.047	0.019	9.762	35.428	0.765	0.000	0.000
VLDLR	2.344	-4.057	0.227	0.071	0.022	1.016	-6.105	0.487	0.427	0.312	3.189	-2.403	0.309	0.003	0.002
WDPCP	0.994	-6.215	0.035	0.481	0.323	2.409	-3.823	0.278	0.045	0.018	4.070	0.690	0.137	0.000	0.000
WNT4	0.646	-6.498	0.038	0.663	0.520	-2.000	-4.671	-0.135	0.099	0.048	3.020	-2.916	0.438	0.005	0.003
YJEFN3	1.621	-5.417	0.067	0.229	0.109	-0.088	-6.611	-0.006	0.953	0.930	6.556	13.003	0.444	0.000	0.000
ZC3HC1	3.108	-2.164	0.285	0.014	0.003	3.086	-2.120	0.382	0.010	0.003	11.963	53.994	0.729	0.000	0.000
ZDHHC8	-2.106	-4.553	-0.251	0.109	0.038	-4.029	0.794	-0.304	0.001	0.000	3.576	-1.130	0.383	0.001	0.000
ZFP36	-6.456	9.921	-1.142	0.000	0.000	-3.665	-0.399	-0.864	0.002	0.000	-10.252	39.369	-1.560	0.000	0.000
ZFP36L1	-4.088	0.842	-0.451	0.001	0.000	-2.682	-3.179	-0.660	0.026	0.009	-5.050	4.939	-0.523	0.000	0.000
ZFP36L2	-0.023	-6.705	-0.002	0.989	0.982	-2.591	-3.399	-0.196	0.031	0.011	-1.038	-6.884	-0.183	0.360	0.300
ZNF764	0.661	-6.488	0.021	0.654	0.510	3.016	-2.312	0.497	0.012	0.003	10.770	43.670	0.730	0.000	0.000

Supplementary Table S6. Associatation of gene expression with patients' OS in TCGA-LIHC dataset

id	gene_name	HR	P.value	P.adj
ENSG00000001167.14	NFYA	1.653250839	0.004906774	0.061645797
ENSG00000001630.17	CYP51A1	0.934948724	0.703283405	0.8463914
ENSG00000003402.20	CFLAR	1.087305324	0.63497986	0.8047065
ENSG00000004487.17	KDM1A	1.896546462	0.000359377	0.021861457
ENSG00000004864.14	SLC25A13	0.760965617	0.124247461	0.331302084
ENSG00000005339.15	CREBBP	0.987065579	0.940938893	0.973595896
ENSG00000005381.8	MPO	1.045793901	0.799083119	0.90118652
ENSG00000005421.9	PON1	0.59151812	0.003311231	0.05166836
ENSG00000005471.19	ABCB4	0.774498078	0.147763527	0.365126306
ENSG00000006025.12	OSBPL7	1.114724668	0.53662252	0.738316219
ENSG00000006327.14	TNFRSF12A	1.473800434	0.029395353	0.150878312
ENSG00000006712.15	PAF1	1.678565209	0.003712674	0.054200456
ENSG00000007062.12	PROM1	0.991592795	0.961652397	0.98286138
ENSG00000007944.15	MYLIP	1.337546988	0.100569707	0.294220465
ENSG00000010270.14	STARD3NL	1.860254105	0.000522937	0.024742507
ENSG00000010278.15	CD9	1.205785021	0.290084385	0.52917445
ENSG00000010327.10	STAB1	1.401667	0.057853426	0.217687164
ENSG00000010671.16	BTK	1.171103685	0.370679213	0.606337924
ENSG00000011143.19	MKS1	1.203330058	0.292238926	0.531611298
ENSG00000011422.12	PLAUR	1.551314459	0.01460657	0.105271528
ENSG00000012171.20	SEMA3B	0.89322362	0.521907629	0.727125039
ENSG00000012174.12	MBTPS2	1.247804232	0.209501482	0.441923566
ENSG00000012504.15	NR1H4	0.929649456	0.677911692	0.830891492
ENSG00000012660.14	ELOVL5	0.96102054	0.821508927	0.913212915
ENSG00000015520.15	NPC1L1	1.185440614	0.334572594	0.572857693
ENSG00000016082.15	ISL1	NA	NA	NA
ENSG00000021461.17	CYP3A43	0.613605824	0.00624184	0.068741499
ENSG00000021762.20	OSBPL5	1.004284907	0.980675536	0.990777292
ENSG00000022267.19	FHL1	0.918360857	0.628320989	0.800444687
ENSG00000023171.18	GRAMD1B	1.521676307	0.018186222	0.117166882
ENSG00000025423.11	HSD17B6	0.742324697	0.091267519	0.279480965
ENSG00000025434.19	NR1H3	1.505226743	0.021585064	0.128193878
ENSG00000036530.9	CYP46A1	1.280886827	0.161918507	0.384200164
ENSG00000037280.16	FLT4	0.862616969	0.402262255	0.633979928
ENSG00000038945.15	MSR1	1.386466555	0.065744581	0.233408179
ENSG00000039650.12	PNKP	1.106418841	0.564716807	0.757488923
ENSG00000042088.14	TDP1	1.702625897	0.003002872	0.049740323
ENSG00000044446.12	PHKA2	1.559071809	0.012389497	0.097071979
ENSG00000049239.13	H6PD	1.120282556	0.520371001	0.726394158
ENSG00000049247.14	UTS2	0.815215263	0.248615053	0.486453478
ENSG00000050165.18	DKK3	0.701047085	0.045373178	0.191877749
ENSG00000050405.13	LIMA1	1.394194203	0.059903612	0.221779673
ENSG00000052802.13	MSMO1	1.051079238	0.776953441	0.888738091
ENSG00000054282.16	SDCCAG8	1.081390257	0.658401697	0.819369894
ENSG00000054654.19	SYNE2	0.874088887	0.445231402	0.670025242
ENSG00000057252.13	SOAT1	1.398758712	0.056751466	0.21531355
ENSG00000059804.16	SLC2A3	1.10332209	0.576815675	0.764287828
ENSG00000060566.14	CREB3L3	1.230162221	0.24194625	0.479142205
ENSG00000064419.13	TNPO3	1.592447413	0.009030815	0.082665506
ENSG00000064687.13	ABCA7	1.117147799	0.529301028	0.732832324
ENSG00000066135.13	KDM4A	1.422362731	0.045996814	0.193283723
ENSG00000066136.20	NFYC	1.642690569	0.005295767	0.063657378
ENSG00000066926.12	FECH	1.327509511	0.109229767	0.308165689
ENSG00000067064.11	IDI1	1.417221724	0.049100473	0.199949072

ENSG00000067992.16	PDK3	1.521032862	0.017854655	0.116044538
ENSG00000068305.17	MEF2A	0.951218689	0.776901367	0.888738091
ENSG00000068366.20	ACSL4	1.365975016	0.080418194	0.261491954
ENSG00000069764.10	PLA2G10	1.167573344	0.378801315	0.614337964
ENSG00000070018.9	LRP6	1.361996802	0.082801674	0.26544283
ENSG00000070882.13	OSBPL3	1.53667994	0.015285421	0.10788047
ENSG00000071553.18	ATP6AP1	1.508076563	0.020505339	0.124458497
ENSG00000072310.17	SREBF1	1.180725108	0.344430451	0.582296547
ENSG00000072506.13	HSD17B10	1.224808428	0.25104567	0.488695978
ENSG00000072778.20	ACADVL	0.860458428	0.394206544	0.627273169
ENSG00000072858.11	SIDT1	0.57032575	0.001768956	0.040067668
ENSG00000073060.16	SCARB1	1.593326945	0.00924582	0.08345402
ENSG00000073734.10	ABCB11	0.776029477	0.150916539	0.369010092
ENSG00000074660.16	SCARF1	1.004894633	0.977910724	0.989646091
ENSG00000075239.14	ACAT1	0.67486108	0.026179792	0.142135373
ENSG00000076555.15	ACACB	0.95295854	0.783927047	0.89311811
ENSG00000076641.4	PAG1	1.373573316	0.072786932	0.246842171
ENSG00000077463.15	SIRT6	1.773515849	0.001381481	0.036605464
ENSG00000078401.7	EDN1	0.97656937	0.89381067	0.951137442
ENSG00000078687.17	TNRC6C	1.23731427	0.226690153	0.462016446
ENSG00000079156.17	OSBPL6	1.188511892	0.326092609	0.564768197
ENSG00000079459.13	FDFT1	1.107461066	0.561930884	0.756118473
ENSG00000080511.5	RDH8	1.42672391	0.045468725	0.192108379
ENSG00000082014.17	SMARCD3	1.474783902	0.028833807	0.149332012
ENSG00000083807.10	SLC27A5	0.672333182	0.025202717	0.139116513
ENSG00000084093.19	REST	1.351778603	0.08833212	0.275224504
ENSG00000084674.15	APOB	0.891558239	0.514047397	0.721984058
ENSG00000084676.15	NCOA1	1.573468946	0.0109748	0.09138394
ENSG00000086062.13	B4GALT1	1.135304839	0.476139437	0.694055284
ENSG00000086696.11	HSD17B2	0.834732222	0.310291507	0.549711459
ENSG00000086730.17	LAT2	1.095171462	0.606390454	0.785888772
ENSG00000086848.15	ALG9	1.068904993	0.704568597	0.847059063
ENSG00000087237.12	CETP	0.831478655	0.295788382	0.534690585
ENSG00000088002.12	SULT2B1	1.402693659	0.055017753	0.212246056
ENSG00000088035.18	ALG6	1.611636772	0.007385324	0.0749609
ENSG00000089351.14	GRAMD1A	1.509514304	0.020534826	0.124583201
ENSG00000090447.12	TFAP4	1.481399845	0.027884461	0.14658275
ENSG00000090905.19	TNRC6A	1.208904896	0.282897404	0.522346715
ENSG00000091039.17	OSBPL8	1.461526998	0.032193273	0.158738049
ENSG00000091583.11	APOH	0.658408652	0.018343874	0.117837845
ENSG00000091732.17	ZC3HC1	1.626963468	0.006432383	0.069957659
ENSG00000091831.24	ESR1	0.497458623	0.000111258	0.014479532
ENSG00000092847.13	AGO1	1.560904412	0.012236049	0.096455986
ENSG00000093010.15	COMT	1.209234481	0.281437067	0.521189123
ENSG00000095637.22	SORBS1	1.179240034	0.353347604	0.59087717
ENSG00000095970.17	TREM2	1.518209966	0.018399239	0.117969543
ENSG00000096717.12	SIRT1	1.067085594	0.713324359	0.852949184
ENSG00000096872.17	IFT74	1.213836751	0.271136242	0.510822059
ENSG00000096968.14	JAK2	1.137401324	0.466163011	0.687030452
ENSG00000096996.16	IL12RB1	1.038266988	0.830907044	0.917983562
ENSG00000097021.20	ACOT7	2.106957393	3.88E-05	0.009907181
ENSG00000099194.6	SCD	1.209968127	0.278956523	0.518216636
ENSG00000099377.14	HSD3B7	1.090604216	0.623537541	0.796938679
ENSG00000099904.16	ZDHHC8	1.175874196	0.358417621	0.595821972
ENSG00000099917.17	MED15	1.914135485	0.000333362	0.021417394
ENSG00000100003.18	SEC14L2	0.691386236	0.03697947	0.171175486

ENSG00000100078.4	PLA2G3	NA	NA	NA	
ENSG00000100079.7	LGALS2		1.058078275	0.747803212	0.872694989
ENSG00000100243.21	CYB5R3		1.958715348	0.000189078	0.017843169
ENSG00000100300.18	TSPO		1.416858059	0.048671796	0.19913736
ENSG00000100342.21	APOL1		0.992932577	0.967943221	0.985429775
ENSG00000100354.21	TNRC6B		1.105518402	0.568948255	0.760074975
ENSG00000100360.15	IFT27		0.960597535	0.819444513	0.912269662
ENSG00000100393.14	EP300		1.371089361	0.07515398	0.251386642
ENSG00000100427.16	MLC1		0.819540139	0.260359643	0.499393151
ENSG00000100504.17	PYGL		1.039030517	0.8277848	0.916427178
ENSG00000100600.15	LGMN		1.473995705	0.02902493	0.149772179
ENSG00000100889.12	PCK2		0.771157857	0.142164572	0.357093343
ENSG00000100906.11	NFKBIA		0.950294947	0.772934078	0.886544024
ENSG00000100934.15	SEC23A		1.231443779	0.236492216	0.473194198
ENSG00000100979.15	PLTP		1.486461369	0.02582626	0.14109321
ENSG00000101255.11	TRIB3		1.521918435	0.017797077	0.115865051
ENSG00000101384.12	JAG1		1.294385652	0.144896391	0.360940951
ENSG00000101473.17	ACOT8		1.791979762	0.001200721	0.03483763
ENSG00000101558.14	VAPA		1.328834462	0.110472216	0.310070962
ENSG00000101670.12	LIPG		0.997355479	0.98797809	0.994179856
ENSG00000101846.8	STS		1.053221112	0.770143236	0.88482138
ENSG00000101849.17	TBL1X		1.456787534	0.03311892	0.161391387
ENSG00000102003.11	SYP		1.010498949	0.952732242	0.978662859
ENSG00000102119.11	EMD		1.48482903	0.026119478	0.141970182
ENSG00000102893.16	PHKB		1.447368698	0.036838135	0.170842611
ENSG00000103051.20	COG4		1.684898419	0.003579717	0.053567133
ENSG00000103375.11	AQP8		0.830628669	0.293502647	0.532907416
ENSG00000104537.17	ANXA13		1.244808214	0.215744136	0.449349369
ENSG00000104549.12	SQLE		1.509587339	0.020678272	0.124882773
ENSG00000104823.9	ECH1		0.957413765	0.805264373	0.905291191
ENSG00000105204.14	DYRK1B		1.253579247	0.200762017	0.431080179
ENSG00000105270.15	CLIP3		1.540607855	0.015673303	0.108733037
ENSG00000105329.11	TGFB1		1.335806142	0.10264551	0.297400615
ENSG00000105369.10	CD79A		0.801512153	0.210943696	0.44389349
ENSG00000105398.4	SULT2A1		0.726075111	0.070075603	0.24155693
ENSG00000105516.11	DBP		1.129793163	0.488985227	0.704274014
ENSG00000105699.16	LSR		1.023196166	0.896544145	0.95226595
ENSG00000105723.13	GSK3A		1.634948506	0.005908218	0.066946616
ENSG00000105755.8	ETHE1		1.018877809	0.915236046	0.961893121
ENSG00000105971.15	CAV2		1.319207098	0.11919443	0.323333861
ENSG00000105974.13	CAV1		1.076599905	0.674861148	0.828841309
ENSG00000106258.15	CYP3A5		0.590212478	0.003218764	0.051073817
ENSG00000106341.11	PPP1R17	NA	NA	NA	
ENSG00000106565.18	TMEM176B		0.747932701	0.099264849	0.291980818
ENSG00000107290.14	SETX		0.98619784	0.93697212	0.971757224
ENSG00000107566.14	ERLIN1		1.040680912	0.820902584	0.913013392
ENSG00000107798.18	LIPA		1.35861159	0.084809835	0.269018309
ENSG00000108424.11	KPNB1		1.581450291	0.009647537	0.085012638
ENSG00000108443.14	RPS6KB1		1.456592783	0.033544605	0.162471364
ENSG00000108479.12	GALK1		1.033027499	0.853732872	0.931289436
ENSG00000108557.19	RAI1		1.230919415	0.238519366	0.475360028
ENSG00000108733.11	PEX12		0.90436296	0.570481871	0.760548842
ENSG00000108786.11	HSD17B1		1.329947601	0.107825195	0.305803003
ENSG00000109084.14	TMEM97		1.197371833	0.306489606	0.545618416
ENSG00000109107.14	ALDOC		1.122358356	0.51153141	0.720268154
ENSG00000109181.12	UGT2B10		0.730436341	0.078374798	0.257320289

ENSG00000109193.12	SULT1E1	0.921556231	0.642564335	0.80974892
ENSG00000109819.9	PPARGC1A	0.571880341	0.001826311	0.040382994
ENSG00000109929.10	SC5D	0.795345719	0.193951831	0.423000006
ENSG00000110047.18	EHD1	1.521898775	0.018593079	0.11865032
ENSG00000110048.12	OSBP	0.980116092	0.909323702	0.958985091
ENSG00000110243.12	APOA5	0.868096603	0.421538595	0.650549568
ENSG00000110244.7	APOA4	1.192278237	0.320053008	0.558812882
ENSG00000110245.12	APOC3	0.621420029	0.007237161	0.073984123
ENSG00000110651.12	CD81	1.135402682	0.474253158	0.692674706
ENSG00000110721.12	CHKA	1.526257503	0.016792608	0.112490939
ENSG00000110777.12	POU2AF1	1.085219842	0.641461485	0.809301007
ENSG00000110921.14	MVK	1.333551309	0.103049132	0.298059623
ENSG00000111012.10	CYP27B1	1.271854326	0.174474918	0.400024924
ENSG00000111319.13	SCNN1A	0.863523446	0.403453456	0.634970061
ENSG00000111581.10	NUP107	1.606595826	0.007760153	0.076519939
ENSG00000111664.11	GNB3	1.021188057	0.905416556	0.956637661
ENSG00000111684.11	LPCAT3	1.244738018	0.213661372	0.446745804
ENSG00000111897.7	SERINC1	0.954272739	0.790218541	0.896380151
ENSG00000112033.14	PPARD	1.624251835	0.006524312	0.070410606
ENSG00000112115.7	IL17A	NA	NA	NA
ENSG00000112116.10	IL17F	NA	NA	NA
ENSG00000112175.8	BMP5	0.877502605	0.457956746	0.680325382
ENSG00000112212.12	TSPO2	1.532547103	0.016407093	0.111266249
ENSG00000112293.15	GPLD1	0.620817522	0.007456938	0.07526866
ENSG00000112304.11	ACOT13	0.916460275	0.619811492	0.794298688
ENSG00000112964.14	GHR	0.647412412	0.01393897	0.102934304
ENSG00000112972.15	HMGCS1	0.993227152	0.969199367	0.985990712
ENSG00000113161.16	HMGCR	1.112226408	0.544941658	0.744727922
ENSG00000113494.17	PRLR	0.902969175	0.561538604	0.756036689
ENSG00000113578.18	FGF1	0.983344724	0.924094168	0.965466196
ENSG00000113580.15	NR3C1	1.11731971	0.529201443	0.732829316
ENSG00000113615.13	SEC24A	1.198070515	0.305891285	0.544804071
ENSG00000113966.10	ARL6	1.497899122	0.022819672	0.131543554
ENSG00000114120.14	SLC25A36	1.155969769	0.411576959	0.641600247
ENSG00000114650.20	SCAP	1.111013736	0.549289086	0.747612025
ENSG00000115020.17	PIKFYVE	1.520802846	0.019741064	0.121973557
ENSG00000115361.8	ACADL	0.654568134	0.016909759	0.112801177
ENSG00000115548.17	KDM3A	1.340226726	0.096084338	0.28759432
ENSG00000115677.17	HDLBP	1.480237549	0.027862676	0.146540245
ENSG00000116005.12	PCYOX1	0.943973927	0.742855118	0.8696574
ENSG00000116127.19	ALMS1	1.427161801	0.045256411	0.191450389
ENSG00000116133.13	DHCR24	1.261750335	0.186117699	0.414019732
ENSG00000116171.19	SCP2	0.736507146	0.083126435	0.265922076
ENSG00000116285.13	ERRFI1	0.765799585	0.131645783	0.342691736
ENSG00000116663.11	FBXO6	1.500309665	0.022870461	0.131720655
ENSG00000117091.10	CD48	0.745800105	0.098257422	0.290451181
ENSG00000117305.15	HMGCL	1.08455648	0.644684578	0.81109901
ENSG00000117528.14	ABCD3	1.148967921	0.4334392	0.660764721
ENSG00000117594.10	HSD11B1	0.831578341	0.296363019	0.535259862
ENSG00000117758.14	STX12	1.136429765	0.467965358	0.688356368
ENSG00000117859.19	OSBPL9	1.581477022	0.010214428	0.088086831
ENSG00000118137.10	APOA1	0.731937781	0.076586488	0.254161607
ENSG00000118402.6	ELOVL4	1.267892686	0.178782623	0.405466286
ENSG00000118515.11	SGK1	0.939893338	0.725158723	0.859647323
ENSG00000118689.15	FOXO3	1.342762285	0.09724495	0.289296223
ENSG00000119138.4	KLF9	0.675269343	0.026517673	0.143108734

ENSG00000119401.11	TRIM32	1.47646083	0.027580603	0.146049865
ENSG00000119655.11	NPC2	1.610340771	0.007196214	0.073917704
ENSG00000119673.14	ACOT2	1.028894672	0.871212004	0.940114364
ENSG00000119915.5	ELOVL3	1.607910749	0.007929726	0.077230791
ENSG00000119927.14	GPAM	0.746174066	0.097885122	0.29003396
ENSG00000120437.9	ACAT2	1.060658977	0.737720143	0.86675713
ENSG00000120837.8	NFYB	0.835944306	0.308540099	0.547648055
ENSG00000120885.22	CLU	0.878790246	0.461938114	0.683763383
ENSG00000120915.14	EPHX2	0.670802241	0.024466076	0.136751571
ENSG00000121068.14	TBX2	0.918000602	0.630479965	0.801755596
ENSG00000121653.11	MAPK8IP1	1.106244413	0.565286857	0.757859151
ENSG00000121853.4	GHSR	NA	NA	NA
ENSG00000122025.15	FLT3	0.671031699	0.025627508	0.140489396
ENSG00000122126.17	OCRL	1.418503897	0.048414664	0.198663779
ENSG00000122188.13	LAX1	0.832935208	0.299563737	0.53856614
ENSG00000122507.21	BBS9	1.308345504	0.126659441	0.33499473
ENSG00000122547.11	EEPD1	1.181932778	0.342928636	0.58075629
ENSG00000122641.11	INHBA	1.063407581	0.727626265	0.860907086
ENSG00000122787.15	AKR1D1	0.694245631	0.03882437	0.175343335
ENSG00000123130.17	ACOT9	1.398732443	0.057622855	0.217232649
ENSG00000123384.14	LRP1	1.074630874	0.681885225	0.833158792
ENSG00000123908.12	AGO2	1.384385395	0.06662743	0.234844341
ENSG00000123983.14	ACSL3	1.677523882	0.003626092	0.053618296
ENSG00000124253.11	PCK1	0.728109661	0.073564057	0.248332586
ENSG00000124313.18	IQSEC2	1.050365323	0.780671072	0.891116541
ENSG00000124391.5	IL17C	NA	NA	NA
ENSG00000125124.13	BBS2	1.021755212	0.902821329	0.955672513
ENSG00000125629.15	INSIG2	1.122532619	0.514261523	0.722068513
ENSG00000125686.12	MED1	1.265995981	0.180587102	0.407576298
ENSG00000125845.7	BMP2	1.667577235	0.004530785	0.059211157
ENSG00000125863.20	MKKS	1.599059125	0.008538668	0.080429876
ENSG00000126070.20	AGO3	1.625357035	0.006860752	0.072375745
ENSG00000127472.11	PLA2G5	0.842573625	0.330713887	0.569420234
ENSG00000127743.6	IL17B	1.230759068	0.242806276	0.479968559
ENSG00000127955.17	GNAI1	1.204967329	0.289963987	0.52917445
ENSG00000128016.7	ZFP36	1.044448254	0.805142909	0.905291191
ENSG00000128039.12	SRD5A3	2.130173939	3.55E-05	0.00960515
ENSG00000128602.11	SMO	1.040981481	0.819417628	0.912269662
ENSG00000128604.20	IRF5	1.466654983	0.031320982	0.156431549
ENSG00000128731.18	HERC2	1.060591518	0.737772945	0.866781547
ENSG00000129152.4	MYOD1	NA	NA	NA
ENSG00000129595.14	EPB41L4A	0.809166652	0.228723758	0.464482529
ENSG00000129988.6	LBP	1.3692165	0.077750527	0.256078204
ENSG00000130164.14	LDLR	0.881550573	0.47525301	0.693536334
ENSG00000130173.13	ANGPTL8	1.119999908	0.519146373	0.725518447
ENSG00000130203.10	APOE	0.896893846	0.538153799	0.739410741
ENSG00000130208.9	APOC1	0.591378988	0.003416222	0.052313632
ENSG00000130589.16	HELZ2	0.988692443	0.948438547	0.976865541
ENSG00000130703.17	OSBPL2	1.536446356	0.01543606	0.108236261
ENSG00000130707.18	ASS1	0.765221864	0.129513155	0.339152763
ENSG00000130948.10	HSD17B3	NA	NA	NA
ENSG00000131018.24	SYNE1	0.860714365	0.394649411	0.627719581
ENSG00000131069.20	ACSS2	1.204304524	0.29129106	0.530437446
ENSG00000131238.18	PPT1	1.700256776	0.002873791	0.048681805
ENSG00000131408.15	NR1H2	1.270777841	0.174040349	0.399599003
ENSG00000131446.17	MGAT1	1.573914466	0.010831589	0.090609118

ENSG00000131748.16	STARD3	1.788811252	0.001221179	0.03493518
ENSG00000131781.13	FMO5	0.880398635	0.46943593	0.689545316
ENSG00000131871.15	SELENOS	1.618733223	0.006742854	0.071762159
ENSG00000131981.16	LGALS3	1.691535703	0.003610683	0.053618296
ENSG00000132170.23	PPARG	1.744954977	0.001788058	0.040086253
ENSG00000132196.15	HSD17B7	1.23796551	0.225223876	0.460346823
ENSG00000132341.12	RAN	2.004721494	0.000112285	0.014492829
ENSG00000132356.11	PRKAA1	1.295328991	0.148354636	0.365570718
ENSG00000132386.11	SERPINF1	0.863427144	0.40304321	0.634693408
ENSG00000132522.16	GPS2	0.963529929	0.83317242	0.91929549
ENSG00000132631.6	SCP2D1	NA	NA	NA	
ENSG00000132671.6	SSTR4	NA	NA	NA	
ENSG00000132693.12	CRP	1.15055671	0.425294105	0.653692998
ENSG00000132855.5	ANGPTL3	0.762893161	0.124824151	0.332338783
ENSG00000132906.18	CASP9	1.178149117	0.351748024	0.589231762
ENSG00000133835.17	HSD17B4	0.70395392	0.048389135	0.198623628
ENSG00000133935.7	ERG28	0.966530279	0.846297096	0.926781236
ENSG00000134184.13	GSTM1	0.91972387	0.634745015	0.804690094
ENSG00000134200.4	TSHB	NA	NA	NA	
ENSG00000134240.12	HMGCS2	0.543381262	0.000717739	0.027975744
ENSG00000134243.12	SORT1	1.417827616	0.047886793	0.197425448
ENSG00000134339.9	SAA2	0.920962959	0.639893095	0.807962409
ENSG00000134490.14	TMEM241	1.530237193	0.01658433	0.112094333
ENSG00000134698.11	AGO4	1.705157646	0.003017126	0.049740323
ENSG00000134809.9	TIMM10	1.120001696	0.521033151	0.726617247
ENSG00000134824.14	FADS2	1.119008884	0.522328016	0.727488101
ENSG00000135046.14	ANXA1	1.062359096	0.730847075	0.862788172
ENSG00000135097.7	MSI1	1.15531082	0.412113821	0.642030453
ENSG00000135100.18	HNF1A	1.263950286	0.1835825	0.410607145
ENSG00000135218.19	CD36	0.961689409	0.824313459	0.914889928
ENSG00000135220.11	UGT2A3	0.999315505	0.996892236	0.998321815
ENSG00000135226.18	UGT2B28	1.09970144	0.590363602	0.774017664
ENSG00000135744.8	AGT	1.087564937	0.633950968	0.804060005
ENSG00000135842.17	NIBAN1	1.09312477	0.612689561	0.789898863
ENSG00000135929.9	CYP27A1	0.645692953	0.014056326	0.103454254
ENSG00000136011.15	STAB2	0.936618716	0.709601167	0.850741999
ENSG00000136305.11	CIDEB	NA	NA	NA	
ENSG00000136710.10	CCDC115	1.3299072	0.110627607	0.310266196
ENSG00000136881.12	BAAT	0.674525474	0.026330667	0.142554233
ENSG00000136883.14	KIF12	1.167620018	0.378837962	0.614337964
ENSG00000137074.20	APTX	1.185396496	0.334393606	0.572696123
ENSG00000137133.11	HINT2	1.014627367	0.934248859	0.970271115
ENSG00000137312.15	FLOT1	1.670276932	0.003997343	0.055680478
ENSG00000137449.17	CPEB2	0.992624062	0.966396474	0.98485376
ENSG00000137561.5	TTPA	0.690745448	0.037757588	0.172979884
ENSG00000137574.11	TGS1	1.400443551	0.057589955	0.217155606
ENSG00000137642.13	SORL1	0.749324748	0.104384099	0.300284543
ENSG00000137700.18	SLC37A4	0.732037936	0.076664597	0.254238838
ENSG00000137714.3	FDX1	0.80635555	0.221155261	0.455191066
ENSG00000137801.11	THBS1	1.217915982	0.262854806	0.501938459
ENSG00000137869.15	CYP19A1	1.886844075	0.000371544	0.022251537
ENSG00000138002.16	IFT172	1.803638152	0.000970257	0.031492642
ENSG00000138061.12	CYP1B1	0.915948029	0.618938486	0.793880428
ENSG00000138075.14	ABCG5	0.66116929	0.019956598	0.122564624
ENSG00000138135.7	CH25H	0.883623416	0.481470877	0.698168879
ENSG00000138379.5	MSTN	0.925510918	0.661866155	0.821425424

ENSG00000138592.14	USP8	1.212706073	0.276389933	0.516000705
ENSG00000138686.10	BBS7	1.746084587	0.001827454	0.040382994
ENSG00000138760.10	SCARB2	1.05928679	0.744262748	0.870552331
ENSG00000138777.20	PPA2	0.949170942	0.767149091	0.883545267
ENSG00000138798.13	EGF	1.536052135	0.015404179	0.108082303
ENSG00000138802.11	SEC24B	0.874650738	0.448836295	0.672324144
ENSG00000138823.13	MTTP	1.006969453	0.968531854	0.985634988
ENSG00000139780.7	METTL21C	NA	NA	NA
ENSG00000140009.18	ESR2	1.075028182	0.68144177	0.832787194
ENSG00000140284.11	SLC27A2	0.745737756	0.097421475	0.289388711
ENSG00000140386.13	SCAPER	1.378320621	0.071911819	0.245170397
ENSG00000140396.13	NCOA2	1.078378066	0.669331367	0.825284087
ENSG00000140443.15	IGF1R	1.482993568	0.026769226	0.143978331
ENSG00000140459.18	CYP11A1	0.824725956	0.275397702	0.514832917
ENSG00000140463.14	BBS4	1.231290796	0.238144628	0.475129014
ENSG00000140465.14	CYP1A1	1.006214845	0.971941745	0.986826785
ENSG00000140650.12	PMM2	1.02000377	0.91057461	0.959637729
ENSG00000140943.17	MBTPS1	1.211653551	0.274426055	0.514047378
ENSG00000140945.17	CDH13	0.922760419	0.648876063	0.813889309
ENSG00000141027.21	NCOR1	0.789217522	0.18100841	0.408085782
ENSG00000141338.14	ABCA8	0.665451444	0.022703178	0.131235324
ENSG00000141447.18	OSBPL1A	1.133916813	0.479547038	0.696984608
ENSG00000141458.13	NPC1	1.599401301	0.008042214	0.07783437
ENSG00000141506.14	PIK3R5	1.076206755	0.677412055	0.830755476
ENSG00000141858.12	SAMD1	1.933961832	0.000249219	0.01961935
ENSG00000142168.15	SOD1	0.827934313	0.28490185	0.524075366
ENSG00000142453.12	CARM1	1.251499	0.201969614	0.432609389
ENSG00000142515.15	KLK3	NA	NA	NA
ENSG00000142606.16	MMEL1	1.09198071	0.618003241	0.793198413
ENSG00000142615.8	CELA2A	NA	NA	NA
ENSG00000143365.19	RORC	0.627227516	0.008794844	0.081879137
ENSG00000143641.10	GALNT2	1.247221604	0.209682582	0.44224432
ENSG00000143815.15	LBR	1.683575828	0.004071644	0.056091301
ENSG00000143921.9	ABCG8	0.662639832	0.020589459	0.124716192
ENSG00000143951.16	WDPCP	1.002559377	0.988383934	0.994249233
ENSG00000144061.14	NPHP1	1.200866979	0.297712904	0.536664335
ENSG00000144063.4	MALL	0.940339507	0.726908695	0.860698559
ENSG00000144452.15	ABCA12	1.571131542	0.011385511	0.093254394
ENSG00000144645.14	OSBPL10	1.086861295	0.635762723	0.805226076
ENSG00000144852.19	NR1I2	0.685761841	0.032571117	0.159786181
ENSG00000144891.18	AGTR1	1.000752576	0.996595789	0.998292186
ENSG00000145214.14	DGKQ	1.037902196	0.832371767	0.918710908
ENSG00000145384.4	FABP2	NA	NA	NA
ENSG00000145545.12	SRD5A1	0.859059174	0.389536071	0.623473309
ENSG00000145626.12	UGT3A1	0.960331571	0.81793292	0.91151846
ENSG00000146070.17	PLA2G7	1.224162678	0.251467272	0.489024356
ENSG00000146233.8	CYP39A1	0.973507141	0.878688169	0.94389241
ENSG00000146278.11	PNRC1	1.28587034	0.154821794	0.374375495
ENSG00000146648.19	EGFR	1.00144213	0.993459352	0.996759272
ENSG00000147155.11	EBP	1.112432851	0.544869857	0.744667392
ENSG00000147383.11	NSDHL	1.593155543	0.009490653	0.084342555
ENSG00000147465.12	STAR	1.284300276	0.155321105	0.375003774
ENSG00000147475.17	ERLIN2	1.034890067	0.845075568	0.926210226
ENSG00000147571.5	CRH	NA	NA	NA
ENSG00000147588.7	PMP2	NA	NA	NA
ENSG00000147676.14	MAL2	1.227464168	0.244889254	0.482343618

ENSG00000147852.16	VLDLR	1.501752916	0.021648551	0.128401904
ENSG00000148175.13	STOM	0.714720071	0.058225128	0.218329592
ENSG00000148377.6	IDI2	NA	NA	NA
ENSG00000148411.8	NACC2	1.270283977	0.175342152	0.401226716
ENSG00000148795.7	CYP17A1	0.762914418	0.126975153	0.335338283
ENSG00000148965.10	SAA4	0.984348359	0.928908247	0.967471288
ENSG00000149084.13	HSD17B12	1.632907291	0.00639232	0.069629202
ENSG00000149357.10	LAMTOR1	1.623489995	0.007029219	0.072943979
ENSG00000149485.19	FADS1	1.590747932	0.009180362	0.083290532
ENSG00000149809.16	TM7SF2	0.932512162	0.691341253	0.839423991
ENSG00000150961.15	SEC24D	1.026773141	0.880463228	0.944599698
ENSG00000151092.18	NGLY1	1.43837264	0.040543708	0.179538388
ENSG00000151148.14	UBE3B	1.185769223	0.332870899	0.57138968
ENSG00000151247.12	EIF4E	1.815983357	0.000866286	0.030180658
ENSG00000151466.12	SCLT1	1.507411451	0.022485797	0.130508259
ENSG00000151632.17	AKR1C2	1.259930448	0.195374745	0.42475094
ENSG00000151726.15	ACSL1	0.763400221	0.125562938	0.333401324
ENSG00000152234.16	ATP5F1A	1.318740338	0.1173667	0.320370733
ENSG00000152518.8	ZFP36L2	1.102539107	0.582466511	0.768067484
ENSG00000152700.14	SAR1B	0.696727671	0.041263182	0.181477711
ENSG00000152904.11	GGPS1	1.705931763	0.002725899	0.047606499
ENSG00000153094.24	BCL2L11	0.796586454	0.197197386	0.426982342
ENSG00000153187.20	HNRNPU	1.542941777	0.014577594	0.105161592
ENSG00000154265.16	ABCA5	0.927287961	0.667694686	0.824583243
ENSG00000154803.13	FLCN	1.231448455	0.239736085	0.476514855
ENSG00000154930.15	ACSS1	1.409739219	0.053003006	0.208242023
ENSG00000155066.16	PROM2	1.127877134	0.494476884	0.707590487
ENSG00000155158.20	TTC39B	0.756070333	0.114039222	0.31545491
ENSG00000155252.13	PI4K2A	1.281682398	0.162233689	0.38452552
ENSG00000155363.18	MOV10	1.643950261	0.00559471	0.065369251
ENSG00000155465.20	SLC7A7	1.543710993	0.014953605	0.106496737
ENSG00000156096.14	UGT2B4	1.22921761	0.242811565	0.479968559
ENSG00000156709.15	AIFM1	1.135236874	0.470975454	0.690790721
ENSG00000156804.7	FBXO32	1.022013451	0.90158946	0.954889733
ENSG00000156873.16	PHKG2	1.542793485	0.014739064	0.105808115
ENSG00000157193.18	LRP8	1.649296186	0.004990366	0.062253096
ENSG00000157978.12	LDLRAP1	1.315956987	0.120478718	0.32522526
ENSG00000158270.12	COLEC12	1.893378457	0.000397998	0.022776588
ENSG00000158874.11	APOA2	0.959549465	0.814798967	0.909888537
ENSG00000159167.12	STC1	1.211246042	0.277500932	0.517115843
ENSG00000159348.13	CYB5R1	1.28006824	0.161017045	0.382915876
ENSG00000159459.12	UBR1	1.018646291	0.916769705	0.962307802
ENSG00000159640.17	ACE	1.019981553	0.910871429	0.959651654
ENSG00000160179.19	ABCG1	1.221153238	0.258034012	0.496586144
ENSG00000160200.18	CBS	0.677180202	0.028584394	0.148448305
ENSG00000160285.15	LSS	1.219094111	0.260028854	0.4989708
ENSG00000160752.15	FDPS	1.327874795	0.108580007	0.307068616
ENSG00000160789.22	LMNA	1.607186623	0.007806491	0.076628631
ENSG00000160868.15	CYP3A4	0.62941029	0.0093019	0.083571995
ENSG00000160870.15	CYP3A7	0.992288639	0.964831167	0.984023233
ENSG00000160882.13	CYP11B1	NA	NA	NA
ENSG00000161513.12	FDXR	1.065179323	0.720499848	0.856901317
ENSG00000161921.17	CXCL16	1.485587076	0.026074811	0.141964026
ENSG00000162009.8	SSTR5	NA	NA	NA
ENSG00000162236.13	STX5	1.398770159	0.058710905	0.219486837
ENSG00000162390.18	ACOT11	1.623240162	0.006553713	0.070568422

ENSG00000162409.11	PRKAA2	1.225217681	0.247905587	0.485656698
ENSG00000162430.17	SELENON	1.73093509	0.002247337	0.044463302
ENSG00000162552.15	WNT4	0.98905212	0.950061381	0.977672417
ENSG00000162572.21	SCNN1D	0.842109394	0.330481337	0.569273292
ENSG00000162772.17	ATF3	0.94851616	0.76373338	0.88174447
ENSG00000163083.6	INHBB	1.318167843	0.119323131	0.323525357
ENSG00000163093.12	BBS5	0.973012305	0.876318675	0.942669026
ENSG00000163191.6	S100A11	1.514764234	0.019191774	0.120397857
ENSG00000163297.17	ANTXR2	1.030851363	0.862615148	0.93519933
ENSG00000163328.14	GPR155	1.283129681	0.161131194	0.383153675
ENSG00000163344.6	PMVK	1.018863681	0.915551915	0.961924717
ENSG00000163382.12	NAXE	1.706312262	0.002825992	0.048256303
ENSG00000163631.17	ALB	0.817177444	0.253132291	0.49092474
ENSG00000163818.17	LZTFL1	1.334781382	0.101782354	0.296092054
ENSG00000163956.13	LRPAP1	1.26398516	0.183652561	0.410729909
ENSG00000164111.15	ANXA5	2.177603141	1.88E-05	0.009287753
ENSG00000164211.13	STARD4	1.689842828	0.003225722	0.051090297
ENSG00000164294.14	GPX8	1.246298958	0.2159642	0.44963474
ENSG00000164687.11	FABP5	1.744107654	0.001881312	0.040971138
ENSG00000164690.8	SHH	0.830135345	0.290822279	0.530031582
ENSG00000164850.15	GPER1	1.039680757	0.824757549	0.915007657
ENSG00000164938.14	TP53INP1	0.973083351	0.87715272	0.942991427
ENSG00000165029.17	ABCA1	1.198031073	0.304391788	0.543707404
ENSG00000165030.4	NFIL3	0.975019936	0.885503253	0.947101061
ENSG00000165097.16	KDM1B	1.297031842	0.143278066	0.358740064
ENSG00000165474.8	GJB2	0.850207594	0.357997758	0.595453107
ENSG00000165533.19	TTC8	1.327810914	0.10714803	0.304770115
ENSG00000165637.13	VDAC2	1.553883124	0.013281372	0.100538796
ENSG00000165953.10	SERPINA12	1.41974935	0.047414131	0.196644787
ENSG00000166035.11	LIPC	0.760601296	0.121977992	0.327573834
ENSG00000166090.8	IL25	NA	NA	NA
ENSG00000166148.4	AVPR1A	0.944196937	0.744120296	0.870520359
ENSG00000166311.10	SMPD1	1.249579764	0.212037751	0.444985032
ENSG00000166394.15	CYB5R2	1.373397317	0.073736069	0.248631237
ENSG00000166828.3	SCNN1G	NA	NA	NA
ENSG00000167114.13	SLC27A4	1.421043973	0.048972884	0.199735122
ENSG00000167165.19	UGT1A6	1.236053036	0.229462137	0.464982337
ENSG00000167306.20	MYO5B	0.974148868	0.881663243	0.945319021
ENSG00000167508.12	MVD	1.558484067	0.012158551	0.09627971
ENSG00000167780.12	SOAT2	1.004184696	0.981033128	0.990953646
ENSG00000167910.4	CYP7A1	0.651713125	0.016225208	0.11052249
ENSG00000167972.14	ABCA3	0.895325444	0.530981696	0.734099822
ENSG00000168000.14	BSCL2	0.879307345	0.4652085	0.686205276
ENSG00000168036.18	CTNNB1	1.261429198	0.187394906	0.415110269
ENSG00000168209.5	DDIT4	1.195348753	0.310247743	0.5496699
ENSG00000168306.13	ACOX2	0.770887588	0.14003312	0.353900158
ENSG00000168309.18	FAM107A	0.717500646	0.060584755	0.223011872
ENSG00000168447.11	SCNN1B	1.28979674	0.151075504	0.369265363
ENSG00000168646.13	AXIN2	1.299424729	0.137128395	0.350190226
ENSG00000168675.19	LDLRAD4	0.997905178	0.990558183	0.995465528
ENSG00000168811.7	IL12A	1.366130743	0.077701373	0.255947453
ENSG00000169136.12	ATF5	0.896706783	0.535674345	0.73768655
ENSG00000169174.11	PCSK9	1.459789309	0.033768846	0.163151581
ENSG00000169218.14	RSPO1	NA	NA	NA
ENSG00000169508.7	GPR183	1.072209979	0.691794173	0.839656919
ENSG00000169692.13	AGPAT2	1.293695572	0.148717512	0.366113409

ENSG00000169710.9	FASN	1.371307365	0.07373207	0.248631237
ENSG00000169919.17	GUSB	1.148182756	0.431861557	0.659408734
ENSG00000169951.10	ZNF764	1.329859264	0.106520293	0.303782589
ENSG00000170017.12	ALCAM	1.481387458	0.027364173	0.145444365
ENSG00000170345.10	FOS	1.081269582	0.657666758	0.818970311
ENSG00000170522.10	ELOVL6	0.997384575	0.988156749	0.994179856
ENSG00000170835.16	CEL	0.917003444	0.62437879	0.797650703
ENSG00000170876.8	TMEM43	1.67337065	0.003782493	0.054872497
ENSG00000171234.14	UGT2B7	0.841417863	0.327391921	0.566165612
ENSG00000171720.10	HDAC3	1.443170983	0.039232957	0.176367128
ENSG00000171867.17	PRNP	1.581039215	0.010049308	0.087002118
ENSG00000172005.11	MAL	0.773209792	0.146455391	0.362987314
ENSG00000172345.14	STARD5	0.571918929	0.002050796	0.042740317
ENSG00000172350.10	ABCG4	1.062643455	0.729572931	0.862161752
ENSG00000172458.4	IL17D	1.09998277	0.587804088	0.772047689
ENSG00000172497.9	ACOT12	0.646059507	0.013471887	0.101214069
ENSG00000172817.4	CYP7B1	0.815587852	0.246913758	0.484629522
ENSG00000172893.16	DHCR7	1.230781919	0.238717364	0.475502505
ENSG00000173402.12	DAG1	1.172815483	0.36675887	0.602894011
ENSG00000173409.14	ARV1	1.254712768	0.199690909	0.429871403
ENSG00000173432.12	SAA1	0.942707378	0.737340051	0.866460982
ENSG00000173610.12	UGT2A1	NA	NA	NA
ENSG00000174007.9	CEP19	1.537414938	0.015532393	0.108534301
ENSG00000174483.20	BBS1	1.443009637	0.038921896	0.175631891
ENSG00000174576.10	NPAS4	NA	NA	NA
ENSG00000174697.5	LEP	NA	NA	NA
ENSG00000175336.10	APOF	0.79187374	0.189391432	0.417486262
ENSG00000175445.17	LPL	1.528823176	0.017922003	0.116286819
ENSG00000175535.6	PNLIP	NA	NA	NA
ENSG00000176387.7	HSD11B2	1.116381646	0.532026599	0.734951372
ENSG00000176986.16	SEC24C	1.09818381	0.59408431	0.777201926
ENSG00000177030.17	DEAF1	1.691406985	0.00321404	0.051028774
ENSG00000177058.12	SLC38A9	1.101179776	0.583583872	0.76885265
ENSG00000177200.17	CHD9	1.123138076	0.510337079	0.719211143
ENSG00000177465.4	ACOT4	1.050919931	0.777899894	0.889204849
ENSG00000177469.13	CAVIN1	0.920286794	0.639115881	0.807433
ENSG00000177565.18	TBL1XR1	1.720712154	0.002554275	0.046782282
ENSG00000177731.16	FLII	1.133923214	0.475794641	0.693854081
ENSG00000178075.20	GRAMD1C	0.690272851	0.036327114	0.16934303
ENSG00000178445.10	GLDC	0.962524091	0.828024636	0.916491266
ENSG00000179142.2	CYP11B2	NA	NA	NA
ENSG00000179455.10	MKRN3	1.349670692	0.089608963	0.277147973
ENSG00000179914.5	ITLN1	0.873754903	0.444568556	0.669691676
ENSG00000179941.9	BBS10	1.442366586	0.038081458	0.173634004
ENSG00000180432.6	CYP8B1	0.625066976	0.008261554	0.079233971
ENSG00000180616.9	SSTR2	1.243441077	0.219908556	0.453657161
ENSG00000180900.20	SCRIB	1.422923209	0.047663928	0.196890092
ENSG00000181004.10	BBS12	1.340887245	0.097237987	0.289296223
ENSG00000181092.10	ADIPOQ	NA	NA	NA
ENSG00000181634.8	TNFSF15	1.125090287	0.503150801	0.71396405
ENSG00000182156.10	ENPP7	1.134743209	0.472628349	0.691570238
ENSG00000182533.7	CAV3	NA	NA	NA
ENSG00000182718.18	ANXA2	1.430769423	0.04356322	0.187332228
ENSG00000182827.9	ACBD3	1.30468408	0.133137519	0.344751545
ENSG00000182858.14	ALG12	1.413735909	0.050008202	0.201901834
ENSG00000182866.17	LCK	0.716879737	0.060031133	0.222017399

ENSG00000183921.7	SDR42E2	0.810587097	0.233590372	0.46985372
ENSG00000184009.12	ACTG1	1.576675736	0.010416866	0.089137292
ENSG00000184227.8	ACOT1	0.741862716	0.090502483	0.278596218
ENSG00000184730.11	APOBR	1.264461944	0.184868046	0.412154341
ENSG00000184792.16	OSBP2	1.48046402	0.026124892	0.141970182
ENSG00000184831.14	APOO	1.461921904	0.03236988	0.159279711
ENSG00000184860.10	SDR42E1	0.927799386	0.670460281	0.82594702
ENSG00000185000.12	DGAT1	1.224429523	0.25177662	0.48942618
ENSG00000185420.19	SMYD3	1.521183215	0.017567494	0.115033105
ENSG00000185559.16	DLK1	0.663835277	0.020901899	0.125680677
ENSG00000185591.10	SP1	1.347101187	0.091946404	0.280775364
ENSG00000185615.16	PDIA2	1.129108022	0.494271756	0.707446541
ENSG00000185630.19	PBX1	0.959338103	0.814770964	0.909888537
ENSG00000185650.10	ZFP36L1	1.416926122	0.051852505	0.205722894
ENSG00000185651.15	UBE2L3	1.757051784	0.001623061	0.038881637
ENSG00000185811.19	IKZF1	0.78282099	0.164665068	0.387626639
ENSG00000185813.11	PCYT2	0.779877354	0.159455127	0.380787813
ENSG00000185823.5	NPAP1	NA	NA	NA
ENSG00000185920.16	PTCH1	1.05605278	0.756825587	0.877694935
ENSG00000186104.11	CYP2R1	1.402783682	0.055226793	0.212637746
ENSG00000186350.12	RXRA	0.926617806	0.667524193	0.824583243
ENSG00000186367.7	MINAR2	NA	NA	NA
ENSG00000186480.13	INSIG1	0.755263834	0.116435471	0.319148257
ENSG00000186951.16	PPARA	1.026493219	0.88312075	0.945913769
ENSG00000187134.14	AKR1C1	1.404800943	0.059004821	0.219849475
ENSG00000188042.8	ARL4C	1.274555465	0.170317575	0.394985141
ENSG00000188257.12	PLA2G2A	1.034275298	0.848239491	0.927848883
ENSG00000188313.13	PLSCR1	1.060193835	0.739633991	0.867988561
ENSG00000188603.22	CLN3	1.800650683	0.001072765	0.032813288
ENSG00000188784.4	PLA2G2E	NA	NA	NA
ENSG00000188786.10	MTF1	1.254971761	0.198011234	0.427991385
ENSG00000188846.14	RPL14	1.490085405	0.024485963	0.136779824
ENSG00000189058.9	APOD	0.868079626	0.422406189	0.651097127
ENSG00000196139.14	AKR1C3	1.629755504	0.006041461	0.067607416
ENSG00000196352.16	CD55	1.405115855	0.056331815	0.214593615
ENSG00000196407.12	THEM5	1.029261653	0.869964281	0.9392547
ENSG00000196498.13	NCOR2	1.070132649	0.699914983	0.844612069
ENSG00000196557.13	CACNA1H	0.821130529	0.262375054	0.501745232
ENSG00000196620.10	UGT2B15	0.838997729	0.317898548	0.556991178
ENSG00000197043.14	ANXA6	1.08156955	0.655455881	0.817620879
ENSG00000197386.12	HTT	1.384723319	0.064758883	0.231452727
ENSG00000197658.9	SLC22A24	0.81881367	0.259062295	0.497857187
ENSG00000197728.11	RPS26	1.039736579	0.824398667	0.914909476
ENSG00000197746.14	PSAP	1.471894715	0.028936952	0.149601258
ENSG00000197785.14	ATAD3A	1.717717102	0.002443291	0.04605841
ENSG00000197888.2	UGT2B17	0.886052229	0.492175089	0.706425221
ENSG00000197977.4	ELOVL2	1.302857245	0.140014001	0.353900158
ENSG00000198099.9	ADH4	0.524447272	0.000387799	0.022722346
ENSG00000198189.11	HSD17B11	0.933882563	0.696943678	0.842854147
ENSG00000198286.10	CARD11	0.918301142	0.627810629	0.800044473
ENSG00000198502.6	HLA-DRB5	1.014626169	0.934311722	0.970287754
ENSG00000198610.11	AKR1C4	0.936849177	0.710426628	0.851241538
ENSG00000198646.14	NCOA6	1.384702875	0.065225169	0.232311757
ENSG00000198670.12	LPA	0.625686719	0.008303854	0.079304548
ENSG00000198707.17	CEP290	1.317513109	0.119177447	0.323333861
ENSG00000198793.13	MTOR	1.29013745	0.147981876	0.365287878

ENSG00000198840.2	MT-ND3	0.952769997	0.783211503	0.892927928
ENSG00000198848.13	CES1	0.931557424	0.687295145	0.83648411
ENSG00000198911.12	SREBF2	1.084388353	0.645263623	0.81152603
ENSG00000199075.1	MIR26A1	NA	NA	NA
ENSG00000199082.1	MIR342	NA	NA	NA
ENSG00000199085.3	MIR148A	NA	NA	NA
ENSG00000199158.1	MIR96	NA	NA	NA
ENSG00000201831.1	SNORD115-1	NA	NA	NA
ENSG00000203857.10	HSD3B1	0.860338123	0.392735131	0.626141897
ENSG00000203859.10	HSD3B2	0.870284817	0.429664731	0.657751754
ENSG00000204228.4	HSD17B8	0.748345707	0.100381017	0.293856921
ENSG00000204231.11	RXRB	1.202069564	0.298026209	0.536764436
ENSG00000204264.12	PSMB8	1.191422974	0.319066766	0.558062761
ENSG00000204444.11	APOM	1.025590144	0.885678083	0.947191281
ENSG00000204842.18	ATXN2	1.447583731	0.03621368	0.169076286
ENSG00000205669.4	ACOT6	0.897087362	0.536469866	0.738181227
ENSG00000205808.7	PLPP6	0.650583986	0.015659809	0.108727641
ENSG00000207063.1	SNORD116-1	NA	NA	NA
ENSG00000207604.3	MIR206	NA	NA	NA
ENSG00000207654.4	MIR128-1	NA	NA	NA
ENSG00000207808.1	MIR27A	NA	NA	NA
ENSG00000207839.1	MIR33B	NA	NA	NA
ENSG00000207864.3	MIR27B	NA	NA	NA
ENSG00000207927.1	MIR302A	NA	NA	NA
ENSG00000207932.1	MIR33A	NA	NA	NA
ENSG00000207933.3	MIR9-1	NA	NA	NA
ENSG00000207962.1	MIR30C1	NA	NA	NA
ENSG00000207983.1	MIR613	NA	NA	NA
ENSG00000207990.3	MIR182	NA	NA	NA
ENSG00000208023.3	MIR185	NA	NA	NA
ENSG00000211452.11	DIO1	0.913509539	0.607748254	0.78679606
ENSG00000211582.1	MIR758	NA	NA	NA
ENSG00000212102.1	MIR301B	NA	NA	NA
ENSG00000213366.13	GSTM2	0.960577395	0.819130308	0.912054024
ENSG00000213398.8	LCAT	0.507501368	0.000181733	0.017445072
ENSG00000213585.11	VDAC1	1.645067742	0.005091409	0.062733013
ENSG00000213658.12	LAT	1.111261319	0.54984574	0.747880825
ENSG00000213759.10	UGT2B11	1.157853159	0.406556135	0.638292779
ENSG00000214413.9	BBIP1	1.219573041	0.259640793	0.498414075
ENSG00000214548.18	MEG3	1.077794662	0.670985651	0.826145864
ENSG00000218510.8	LINC00339	1.303973198	0.136287534	0.349101395
ENSG00000221263.1	MIR548P	NA	NA	NA
ENSG00000221983.8	UBA52	1.567140836	0.011542159	0.093970636
ENSG00000231852.9	CYP21A2	1.357341602	0.08414805	0.267892241
ENSG00000231991.4	ANXA2P2	1.59603867	0.008961869	0.082432143
ENSG00000234906.11	APOC2	0.798260927	0.201442625	0.431983202
ENSG00000240224.1	UGT1A5	1.71343527	0.003167619	0.050588458
ENSG00000240583.13	AQP1	0.860331596	0.392304004	0.625864533
ENSG00000241119.2	UGT1A9	0.903100176	0.565511044	0.757859151
ENSG00000241635.8	UGT1A1	1.067481513	0.711108282	0.851615989
ENSG00000242110.8	AMACR	0.814185133	0.243484472	0.480585787
ENSG00000242366.3	UGT1A8	NA	NA	NA
ENSG00000242515.5	UGT1A10	1.353124941	0.086557994	0.272234798
ENSG00000243135.6	UGT1A3	0.697025871	0.042430678	0.184573109
ENSG00000244045.13	TMEM199	1.867804948	0.000485211	0.024325408
ENSG00000244122.2	UGT1A7	1.104879676	0.572300165	0.761472867

ENSG00000244474.6	UGT1A4	0.65934538	0.019554417	0.121293081
ENSG00000250067.12	YJEFN3	1.430657144	0.042328565	0.184333033
ENSG00000254087.8	LYN	1.001133682	0.99489017	0.997358697
ENSG00000254585.5	MAGEL2	1.036530657	0.838378146	0.921994573
ENSG00000254656.3	RTL1	NA	NA	NA
ENSG00000259905.7	PWRN1	0.997437437	0.988366379	0.994249233
ENSG00000261701.7	HPR	0.838204571	0.315327688	0.554403222
ENSG00000265787.2	CYP4F35P	NA	NA	NA
ENSG00000267467.4	APOC4	0.840183329	0.323997994	0.562796824
ENSG00000269404.7	SPIB	1.1963597	0.310701877	0.550015726
ENSG00000271886.1	MIR98	NA	NA	NA
ENSG00000272398.6	CD24	1.259681864	0.190080532	0.417978171
ENSG00000276365.1	MIR145	NA	NA	NA
ENSG00000277494.2	GPIHBP1	0.635471303	0.0105792	0.089894184
ENSG00000277893.2	SRD5A2	0.669522318	0.023959889	0.135264692
ENSG00000278195.2	SSTR3	1.034855095	0.84536697	0.926331116
ENSG00000278535.5	DHRS11	1.058385603	0.74693275	0.872200151
ENSG00000278540.5	ACACA	1.639926151	0.005356992	0.064052015
ENSG00000279050.1	PWAR1	NA	NA	NA
ENSG00000283733.1	MIR146A	NA	NA	NA
ENSG00000283819.1	MIR144	NA	NA	NA
ENSG00000283871.1	MIR130B	NA	NA	NA
ENSG00000284375.1	MIR19B1	NA	NA	NA
ENSG00000284536.1	MIR17	NA	NA	NA

Supplementary Table S7. Univariate and multivariate analyses of factors associated with overall survival in SYSUCC cohort 1.

Variables	Univariate			Multivariate		
	HR	95%CI	*p*	HR	95%CI	*p*
Age, years (median, range)	1.005	0.662-1.527	0.98			
Gender (male/female)	1.314	0.730-2.465	0.344			
HBsAg (negative/positive)	1.258	0.608-2.601	0.536			
AFP, ng/mL (≤/> 400)	1.75	1.154-2.654	0.008	1.683	1.104-2.565	0.015
ALT, U/L (>/≤ 40)	1.453	0.959-2.203	0.078			
TBIL, µmol/L (>/≤ 17.1)	0.903	0.567-1.439	0.668			
Tumor size, cm (≤/> 5)	1.95	1.213-3.136	0.006			NA
Tumor number (multiple/single)	1.747	1.100-2.776	0.018			NA
TNM stage (III-IV/I-II)	2.546	1.670-3.881	0.00001	2.585	1.685-3.965	0.00001
Differentiation (III-IV/I-II)	1.214	0.787-1.872	0.381			
SQLE expression (high/low)	1.609	1.053-2.459	0.028	1.863	1.214-2.860	0.004

Cox proportional hazards regression models were used. Variables associated with overall survival by univariate analysis were adopted as covariates in multivariate analysis and entered into the equation by the forward selection based on likelihood ratio test. NA, not adopted. a Bold indicate significance of *P* value ($P < 0.05$)
